# Electrode Treatments for Redox Flow Batteries: Translating Our Understanding from Vanadium to Aqueous‐Organic

**DOI:** 10.1002/advs.202307209

**Published:** 2023-11-16

**Authors:** Harsh Agarwal, Esha Roy, Nirala Singh, Peter A.A. Klusener, Ryan M. Stephens, Qin Tracy Zhou

**Affiliations:** ^1^ Department of Chemical Engineering and Catalysis Science and Technology Institute University of Michigan Ann Arbor Ann Arbor MI 48109‐2136 USA; ^2^ Shell International Exploration and Production Inc. 3333 Highway 6 South Houston TX 77082 USA; ^3^ Shell Global Solutions International B.V. Energy Transition Campus Amsterdam Grasweg 31 Amsterdam 1031 HW The Netherlands

**Keywords:** aqueous‐organic redox flow batteries, carbon felts, electrocatalysts, electrode treatments, flow batteries, quinones, vanadium

## Abstract

Redox flow batteries (RFBs) are a promising technology for long‐duration energy storage; but they suffer from inefficiencies in part due to the overvoltages at the electrode surface. In this work, more than 70 electrode treatments are reviewed that are previously shown to reduce the overvoltages and improve performance for vanadium RFBs (VRFBs), the most commercialized RFB technology. However, identifying treatments that improve performance the most and whether they are industrially implementable is challenging. This study attempts to address this challenge by comparing treatments under similar operating conditions and accounting for the treatment process complexity. The different treatments are compared at laboratory and industrial scale based on criteria for VRFB performance, treatment stability, economic feasibility, and ease of industrial implementation. Thermal, plasma, electrochemical oxidation, CO_2_ treatments, as well as Bi, Ag, and Cu catalysts loaded on electrodes are identified as the most promising for adoption in large scale VRFBs. The similarity in electrode treatments for aqueous‐organic RFBs (AORFBs) and VRFBs is also identified. The need of standardization in RFBs testing along with fundamental studies to understand charge transfer reactions in redox active species used in RFBs moving forward is emphasized.

## Graphical abstract

Electrodes are often treated chemically to mitigate the voltage losses in redox flow batteries (RFBs) and improve RFBs performance. Here, electrode treatments are compared for vanadium‐based RFBs under similar operating conditions to identify promising treatments for industrial implementation. Treatments used for aqueous‐organic RFBs are the same as VRFBs and an outlook for future research is provided.

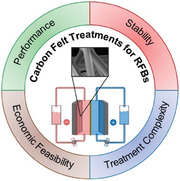

## 1 Introduction to Redox Flow Batteries and Vanadium Redox Flow Batteries

The world is currently undergoing a transition from fossil fuels to renewable energy sources due to environmental concerns. Renewables like solar and wind are expected to supply 50% of the total electricity demand in United States by 2050.^[^ ^1^ ^]^ However, the intermittent nature of solar and wind necessitates the development of energy storage technsologies. Pumped hydroelectric constitutes ∼96% of the total installed energy storage, but additional capacity is restricted due to geographical limitations.^[^ ^2^ ^]^ As a result, there has been an increased interest in development of alternative technologies like electrochemical energy storage.

**Figure 1:**
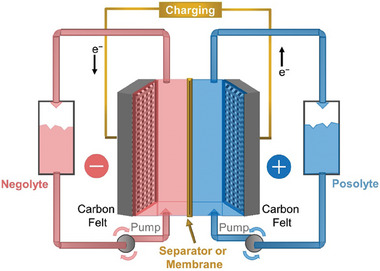
Schematic of a Redox Flow Battery. Carbon felts are commonly used as the negative and positive electrodes. The schematic is not drawn to scale.

Redox flow batteries (RFBs) are one of the promising electrochemical energy storage technologies and have received a widespread interest because of their ability to decouple energy and power. A RFB consists of an electrochemical cell and two tanks which contain electrolytes with redox active species dissolved in them. These electrolytes with redox active species are circulated from the tanks to the electrode surfaces in the electrochemical cell using pumps (**Figure** **1**). The electrode surfaces transfer electrons to/from the active species to store or release energy. The energy and power of an RFB are decoupled: the energy of an RFB is controlled by the concentration of active species and volume of the electrolyte in the tanks, while the power is controlled by the total surface area of the electrodes.^[^ ^2^ ^]^ The two electrode surfaces are separated by an ion‐conducting membrane or separator that allows ions in the electrolyte to pass through, maintaining charge balance. Carbon felts (CF) are the most commonly used electrodes in RFBs due to their high conductivity and high surface area. The redox reactions on CFs often suffer from poor kinetics and reversibility,^[^ ^3^ ^]^ therefore, CFs are frequently treated or loaded with electrocatalysts to catalyze the charge transfer kinetics. However, due to difference in operating conditions and the type of carbon electrodes used in these studies, comparing the improvement in performance and understanding the commercial viability of these treatments is difficult.

Several other types of carbon materials have also been used as electrodes in RFBs. Carbon cloths,^[^ ^4^, ^5^, ^6^, ^7^ ^]^ papers,^[^ ^7^, ^8^, ^9^, ^10^, ^11^ ^]^ foams,^[^ ^12^, ^13^ ^]^ and 3D printed materials^[^ ^14^, ^15^, ^16^ ^]^ are designed to create macro (or nano) pores and improve wettability to enhance ion and mass transport, enhance treatment functionalization, and reduce resistive and pressure drop losses. Since the physicochemical and transport properties of these electrode materials are different and the number of studies are fewer than those of CFs, we limit ourselves to electrode treatments on CFs in this review.

RFBs are classified into aqueous and non‐aqueous based on the nature of the electrolyte. Among aqueous‐based RFBs, inorganic active species spanning the entire *3d* transition metal‐ion range (Ti to Zn) and organic active species like quinones and organometallic complexes dissolved in water have been demonstrated.^[^ ^17^, ^18^ ^]^ Non‐aqueous based RFBs have active species dissolved in organic solvents like dimethyl sulfoxide or acetonitrile instead of water, providing an added advantage of expanded electrochemical window of operation. Compared to aqueous‐based RFBs, non‐aqueous RFBs suffer from high cost, low conductivity, low chemical stability, and low solubility of active species. The low solubility and low stability of active species in non‐aqueous solvents limit the ability of non‐aqueous RFBs to achieve high capacities with long operation times (> 20 years, > 10 000 cycles) desirable for RFBs.^[^ ^2^, ^18^ ^]^ Thus, the treatments of electrodes for aqueous‐based RFBs form the focus of this review.

The Vanadium Redox Flow Battery (VRFB) is the most mature commercial aqueous‐based RFB technology.^[^ ^19^ ^]^ VRFB technology (VO_2_^+^/VO^2+^ // V^2+^/V^3+^) has the advantage of using the same active element (V) on both sides of the battery eliminating the issue of cross‐contamination and extending the lifetime of the battery. Sulfuric acid (H_2_SO_4_) is the most commonly used acid in preparing the electrolyte for VRFBs because of its low cost, high conductivity, and ability to dissociate high concentrations of vanadium salts. Electrolytes prepared using hydrochloric acid (HCl) and mixed acids (HCl and H_2_SO_4_) have been shown to improve performance for VRFBs; however, the evolution of Cl_2_ during battery operation is a safety hazard limiting the widespread adoption.^[^ ^20^, ^21^, ^22^, ^23^, ^24^, ^25^ ^]^ The reactions occurring in VRFBs during discharge are shown below:

(1) $$V^{2+}⇆V^{3+}+e^{-}E^{o}=-0.255\;V\;\mathrm{versus}\;\mathrm{SHE}$$

(2) $${\mathrm{VO}}_{2}^{+}+2H^{+}+e^{-}⇆{\mathrm{VO}}^{2+}+H_{2}{O\;E}^{o}=1.0\;V\;\mathrm{versus}\;\mathrm{SHE}$$

Here E^o^ is the standard redox potential for the respective reactions and SHE is standard hydrogen electrode. The standard cell potential provided by VRFB is 1.255 V, but higher operating voltages can be obtained due to the highly acidic environment.

The V^2+^/V^3+^ and VO_2_^+^/VO^2+^ reactions on CFs at the negative and positive electrodes suffer from slow kinetics and poor electrochemical reversibility, which reduces the voltage efficiency (VE) and subsequently the energy‐efficiency (EE). Eliminating the kinetic overvoltages is expected to increase the round‐trip EE from ∼77 to 86%.^[^ ^26^ ^]^ Therefore, CFs are often treated by the application of heat, plasma, oxidation or loaded with electrocatalysts to improve the charge transfer kinetics.

A wide variety of electrode treatments and electrocatalysts have shown improved VRFB performance and kinetics of vanadium redox couples in the laboratory scale; however, there is a lack of comparison or systematic understanding of which treatment or electrocatalyst has the most impact on VRFB performance and can be scaled up industrially. Several review papers have recently been published that focus on different electrode treatments and electrocatalysts for VRFBs. These reviews provide a fundamental perspective on the influence of treatments on physical and chemical properties of electrodes and the subsequent effect on charge transfer mechanism and reaction kinetics.^[^ ^3^, ^27^, ^28^, ^29^, ^30^, ^31^, ^32^, ^33^, ^34^, ^35^, ^36^, ^37^, ^38^, ^39^, ^40^ ^]^ Most of these studies highlight the need of comparing different treatments in similar conditions and identifying scalable cost‐effective treatments.^[^ ^27^, ^28^, ^33^, ^34^, ^36^, ^37^, ^39^ ^]^ The difficulty of comparing the performance of VRFBs and kinetics of vanadium redox couples for a variety of treatments and electrocatalysts arises because: i) different operating conditions and ii) different sources of CF which affect the performance of VRFB with untreated CFs. Additionally, these studies do not include cost analysis of the treatments. Our review attempts to address this gap by comparing the performance, stability, and economic feasibility of all the treatments under identical conditions and providing an industrial perspective on the costs associated with scale up of certain treatments.

In this review, we first discuss the different voltage losses in RFBs, elaborate on the challenges in comparing different treatments and electrocatalysts, and provide insights into how mechanistic understanding can be used to identify promising treatments. We address the challenges outlined above by extracting and comparing the performance and stability data of VRFBs with different treatments and electrocatalysts on CFs operating under similar conditions from literature over the last 40 years at laboratory and industrial scale. We evaluate the performance using the EE at various operating current densities and stability based on the change in EE per cycle. We determine the affordable capital cost (ACC), defined as the maximum capital cost that can be invested in a treatment without an increase in the overall capital cost of VRFB with untreated electrodes, to identify which treatments can be economically feasible. We identify the treatments that can be implemented easily in industry based on the treatment complexity. We conduct a preliminary capital cost analysis for these high performing, stable, and economically feasible treatments that are easy to implement industrially by identifying the similarities in production of carbon fibers and CFs. We identify the electrode treatments that improve the performance of aqueous‐organic RFBs (AORFBs) are the same as VRFBs, which can potentially arise due to comparable charge transfer mechanism of organic and vanadium redox couples. Finally, we emphasize the need of standardization of RFB testing procedures across literature for fast industrial implementation as we move towards a society powered by renewable energy.

Our analysis shows that thermal, plasma, electrochemical oxidation, CO_2_ treatments, and Bi, Ag, and Cu catalysts loaded on electrodes are easy to implement industrially and satisfy the performance, stability and economic feasibility criteria. A preliminary capital cost analysis for these shortlisted treatments shows that these treatments cost < 40 $ m^−2^, making them attractive for scaling‐up industrially. The qualitative similarity of the treatments used for improving performance of VRFBs and AORFBs can be used to improve on the sluggish kinetics of stable organic molecules discovered in future for use in AORFBs and focus on treatments that can be scaled up industrially.

## 2 Electrode Treatments: Need, Challenges, and Influence of Charge Transfer Mechanism

### 2.1 Voltage Losses in Redox Flow Batteries

The slow charge transfer kinetics, cell resistances, and concentration gradient of active species between the electrode and bulk electrolyte lead to voltage losses in RFBs. The thermodynamic voltage that can be obtained from a battery is the difference in the redox potential of the reactions occurring at the two electrodes. However, due to the inefficiencies in RFBs, the charging of the battery requires more voltage than the thermodynamic voltage. Similarly, the battery delivers less voltage than the thermodynamic voltage during discharging. This excess voltage required for charging or lost during discharging of the battery is called the overvoltage (η). The overvoltage will generally increase with increasing current density. The total overvoltage has contributions from kinetic, ohmic, and mass transfer overvoltage as discussed below:^[^ ^41^ ^]^

1. *Kinetic or activation overvoltage* (η_kinetic_): Overvoltage that arises from slow charge transfer kinetics of reactions occurring at the electrode surface. η_kinetic_ can be reduced by electrode treatments or using electrocatalysts that enhance charge transfer kinetics. That is, some treatments and electrocatalysts will allow you to operate at the same current density, but lower η_kinetic_. The current density or the charge transfer reaction rate increases exponentially with increase in η_kinetic_ based on various models for charge transfer kinetics.
2. *Ohmic overvoltage* (η_ohmic_): Overvoltage that arises from resistances throughout the cell, including resistance from solution, membrane, and electrical connections. The conductivity and hydrophilicity of the electrode can be improved through various treatments to reduce η_ohmic_. η_ohmic_ is linearly related to current density, through Ohm's law (i.e., η_ohmic_ =  *i*R, where *i* is the operating current density and R is the cell resistance multiplied by the electrode area).
3. *Mass transfer or concentration overvoltage* (η_MT_): Overvoltage that arises from the gradient in concentration of active species between the electrode surface and the electrolyte bulk. These gradients arise when the charge transfer rate is faster than the transport rate to replenish the active species via diffusion at the electrode surface. η_MT_ can be reduced by using active species with high diffusivities, increasing the flow rates of the electrolytes, and using less porous electrodes.

**Figure 2:**
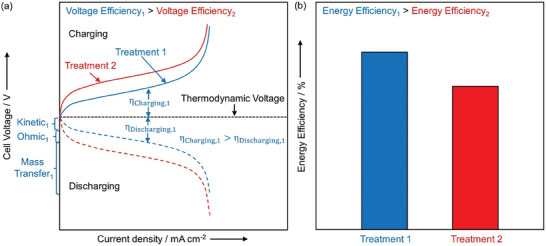
Illustrative plots of a) Cell voltage versus current density for two different electrode treatments (Treatment 1 and 2) in both charging and discharging mode. The different overvoltage contributions for Treatment 1 are shown. b) Comparing energy efficiency for the two treatments in part (a) at a particular current density, assuming coulombic efficiency remains unchanged.

**Figure** **2**a shows the cell voltage as a function of current density during charging and discharging of a RFB with electrodes prepared using two treatments. At low current densities, the charge transfer rate of reactions occurring at the electrode surface is much slower compared to the diffusion of active species to reach the electrode surface. Resultantly, there is no concentration gradient of active species between the electrode surface and bulk electrolyte and η_MT_ is negligible at low current densities. The contributions of η_ohmic_ are also small at low current densities due to minor *iR* drop for a fixed cell resistance. Thus, η_kinetic_ dominates the overall voltage losses at low current densities. η_kinetic_ is larger with slower charge transfer because more driving force is required for charge transfer reaction to occur at desired rates for achieving the same operating current density. These low current densities at which η_kinetic_ dominates are often called kinetic current densities. Treatment 1 has a lower η_kinetic_ compared to Treatment 2 as shown in Figure 2a. At moderate operating current densities, the *iR* drop due to resistances in the cell contributes along with the η_kinetic_, while η_MT_ is still negligible due to the absence of active species concentration gradients. The shape of the cell voltage versus current density appears linear in the region of moderately operating current densities because the additional increase in current density from the kinetic current densities is mostly dominated by η_ohmic_. At extremely high operating current densities, the charge transfer rate is much faster (because of the exponential dependence of charge transfer rate on overpotential) than the diffusion of active species to reach the electrode surface which gives rise to a concentration gradient. Consequently, the current drawn out of the battery is limited by the active species reaching the electrode surface for charge transfer giving rise to η_MT_. These extremely high operating current densities where η_MT_ limits the battery performance is called limiting current densities. As depicted in Figure 2a, Treatment 1 also has a lower η_ohmic_ and η_MT_ compared to Treatment 2.

The voltage losses in the RFB reduce the round‐trip EE of the battery. The EE of the battery is defined as the product of VE and coulombic efficiency (CE). VE is defined as the ratio of the discharging to the charging voltage. The voltage losses decrease the discharging voltage and increase the charging voltage, thereby reducing the VE. CE is defined as the ratio of output charge to the input charge of the battery. CE is reduced due to cross‐over of active species and enhancement of side reactions with larger voltage losses. A treatment would improve the performance by reducing the overvoltage contributions to operate at a fixed current density. Treatment 1 has lower total overvoltages than Treatment 2 at any fixed current density as shown in Figure 2a, leading to higher VE. Assuming the CE remains unchanged for both the treatments, the overall round‐trip EE of the RFB is higher in Treatment 1 compared to Treatment 2 (Figure 2b).

### 2.2 Challenges in Comparing Different Treatments

A plethora of treatments have been tested for RFBs to reduce the overvoltages. Generally, these treatments are shown to enhance the charge transfer kinetics of the individual redox couples used in RFBs in the half‐cell configuration and then tested in full‐cell to capture the improvement in overall RFB performance. However, comparing the charge transfer kinetics in half‐cell and performance in the full‐cell configuration across the literature is not straightforward.

Half‐cell kinetic data and full‐cell performance data collected under different electrochemical conditions cannot be used to compare different treatments. Kinetic data of redox couples are generally captured in half‐cell by evaluating the anodic and cathodic peak separation (Δ *E_p_* = *E_pa_*  − *E_pc_*) or charge transfer resistance (*R_ct_*) which provides an estimate of how fast the electron transfer occurs at the electrode surface. The ratio of anodic and cathodic peak currents $\left(\frac{i_{pa}}{i_{pc}}\right)$ provides an estimate of the kinetic reversibility of the reaction. For a completely reversible reaction, $\Delta \;E_{p}=\left(\frac{59}{n}\right)\;mV$ at 25 °C, where *n* is the number of electrons involved in the reaction and $\frac{i_{pa}}{i_{pc}}\approx 1$.^[^ ^41^, ^42^ ^]^ Cyclic voltammetry (CV) at a fixed scan rate is used to capture Δ*E_p_* and $\frac{i_{pa}}{i_{pc}}$ and electrochemical impedance spectroscopy (EIS) at a fixed potential is used to evaluate *R_ct_*. However, Δ*E_p_* and $\frac{i_{pa}}{i_{pc}}$ are dependent on scan rate and concentration of active species and *R_ct_* is dependent on the choice of potential. Due to the variation in the choice of scan rate and concentration of active species for CVs and potential for EIS across different studies in literature, CVs and EIS can only be used to determine qualitatively whether the treatment improves the kinetics of a redox couple or not and thereby limiting their scope. An alternative is to evaluate the rate constant (*k*) for charge transfer in redox couple that can be compared for different treatments across literature. Δ*E_p_* evaluated at different scan rates from CVs can be used to estimate *k* for quasireversible reactions using the method of Nicholson.^[^ ^41^ ^]^ *R_ct_* at zero overvoltage can be used to evaluate exchange current density that is directly related to *k* using Butler‐Volmer equation.^[^ ^26^, ^41^ ^]^ However, there have been studies reporting wide variations in *k* for a fixed redox couple across different laboratories,^[^ ^43^ ^]^ emphasizing the need to develop standardizing procedures for half‐cell measurements to evaluate *k* across the scientific community. Full‐cell performance of RFBs is usually captured by evaluating the CEs and VEs (or EEs) of the RFB at a wide variety of operating geometric current densities. RFBs are often cycled multiple times by charging and discharging between fixed voltages and their CEs, VEs, and EEs are reported to capture their stability. However, the operating current densities and cycles of operation for different treatments vary across studies, making them difficult to compare due to the changing overvoltage contributions with current density. Additionally, a large proportion of the studies do not report the performance metrics of RFBs with untreated CFs as electrodes. The variation in performance of RFBs with untreated CFs for CFs purchased from different vendors adds to the complexity. Thus, there is an urgent need to standardize the RFB testing protocol that provides guidance to researchers on the conditions under which battery needs to be tested for fair comparison with studies in literature. This standardization will help in utilizing the findings more effectively by the broader scientific community.

The development of novel electrode treatments and electrocatalysts has been an active area of research over the past four decades. Thus, there is a treasure‐trove of performance data for RFBs with different treatments available in literature. We use the available performance data to compare different treatments by (i) interpolating/extrapolating performance parameters to a fixed current density, (ii) comparing the performance improvements across treatments relative to untreated CFs under the same other conditions (e.g., membrane, electrolyte), and (iii) comparing at a fixed number of cycles. We choose VRFBs in this study because VRFBs are the most extensively studied RFBs in the available literature.

### 2.3 Mechanistic Understanding Explains Why Treatments are Effective

Fundamental studies to decipher charge transfer mechanism are essential to understand why certain treatments are effective while others are not. The mechanistic understanding of the charge transfer processes can be used to identify properties that control charge transfer and develop treatments that lower the overvoltages.

Charge transfer at the electrode surface can occur with or without the formation of chemical bonds between the reactant and the electrode surface. The reactant molecule can directly approach the double layer region of the electrode surface and the electron transfer can occur by tunnelling without the formation of a chemical bond, resulting in an outer‐sphere charge transfer. The rates of outer‐sphere charge transfer are predominantly controlled by the electrolyte properties, rather than the electrode. Alternatively, the reactant can chemically bind (directly or indirectly) to the electrode surface forming an adsorbed intermediate while undergoing the electron transfer. This form of electron transfer that involves formation of an adsorbed intermediate is called an inner‐sphere charge transfer. The rate of an inner‐sphere charge transfer depends on the stability of the adsorbed intermediate formed in the rate determining step (RDS) and the energy of transition states.^[^ ^41^ ^]^

The rates of inner‐sphere charge transfer reactions vary by several orders of magnitude depending on the electrode, while the effects of the electrode are minor (a factor of 3–8 different) for outer‐sphere charge transfer reactions.^[^ ^41^, ^44^, ^45^, ^46^, ^47^, ^48^ ^]^ The small effect of the electrode on outer‐sphere charge transfer reactions arises due to the weak interaction of the reactant with the electrode. Electrode surface treatments can lead to minor effects in rate of outer‐sphere reactions since the surface treatments often alter the hydrophilicity of the electrode which in turn can affect the double layer structure.^[^ ^41^, ^49^, ^50^ ^]^ On the contrary, the large effect of electrodes on rates observed for inner‐sphere charge transfer reactions arises because the electrode controls the energy of the adsorbed intermediate, and hence the activation energy and the apparent frequency factor. The activation energy is related to the energy of the transition state formed in the RDS, while the frequency factor is related to the number of active sites for the reaction.^[^ ^17^ ^]^ The surface treatments can improve the inner‐sphere charge transfer rate by reducing the kinetic overvoltage in two possible ways:

1. Provide an alternate reaction pathway with a lower activation energy.
2. Provide more active sites or electrode surface area and increase the apparent frequency factor.

**Figure 3:**
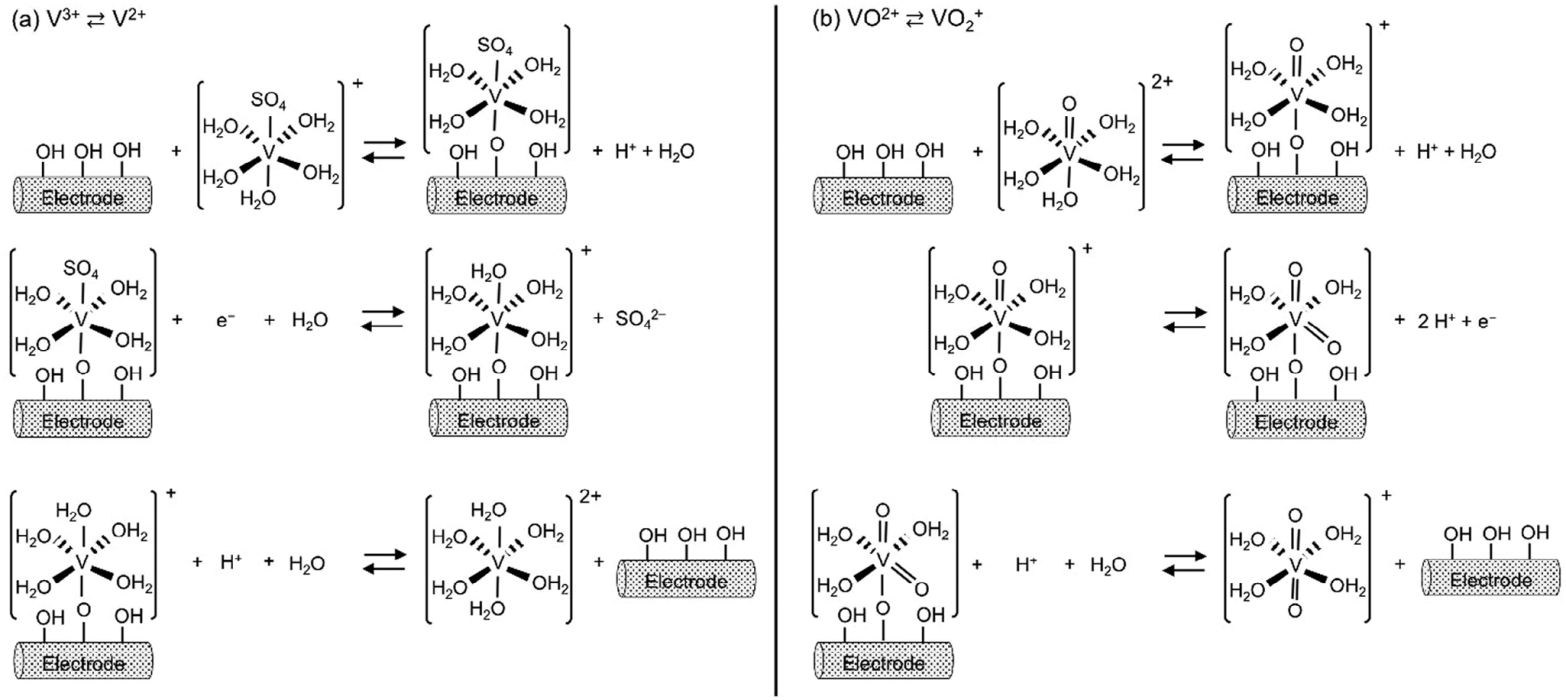
Proposed charge transfer mechanisms for redox couples in vanadium redox flow batteries during charging in sulfuric acid on carbon electrodes considering solvation of vanadium ions, (a) V^3+^/V^2+^ and b) VO^2+^/VO_2_^+^. The V^3+^/V^2+^ mechanism is based on the findings of references^[^ ^17^, ^26^ ^]^ and VO^2+^/VO_2_^+^ is based on references.^[^ ^53^, ^56^, ^57^, ^58^, ^59^ ^]^ The starting complex structures are based on findings of references.^[^ ^25^, ^60^, ^61^, ^62^, ^63^, ^64^, ^65^, ^66^, ^67^ ^]^ The exact charge of the adsorbed complex is unknown and so a best approximation is given, consistent with charge balance.

The surface treatments introduce functional groups like hydroxyl (*OH) on the electrode surface which are hypothesized to be the active sites for V^2+^/V^3+^ and VO_2_^+^/VO^2+^ redox couples in H_2_SO_4_ for VRFBs. There are conflicting studies in literature that describe V^2+^/V^3+^ and VO_2_^+^/VO^2+^ as inner‐ and outer‐sphere reactions.^[^ ^3^, ^10^, ^51^, ^52^ ^]^ The proposed inner‐sphere charge transfer mechanisms on carbon electrodes in sulfuric acid involve the formation of an oxygen bridged adsorbed intermediate for both V^2+^/V^3+^ and VO_2_^+^/VO^2+^ reactions as shown in **Figure** **3**. The solvation of vanadium ions by the electrolyte influences the structure of the vanadium species reaching the electrode surface and forming the adsorbed intermediate. Altering the functional groups on the surface changes the structure and stability of adsorbed intermediate, affecting the overall rate of the charge transfer process.^[^ ^3^, ^26^, ^53^, ^54^, ^55^ ^]^

*OH on the electrode surface is proposed to act as a bridge for electron transfer in the V^2+^/V^3+^ redox couple. The V^3+^ ions in aqueous H_2_SO_4_ are complexed with sulfate or bisulfate (SO_4_^2−^/HSO_4_^−^) anions in solution due to their substitutionally labile nature forming [V(H_2_O)_5_SO_4_]^+^ (or [V(H_2_O)_5_HSO_4_]^2+^ in case of bisulfate). V^2+^ exists as [V(H_2_O)_6_]^2+^ due to its substitutionally inert nature.^[^ ^26^, ^60^, ^61^ ^]^ We depict [V(H_2_O)_5_SO_4_]^+^ as the reactant in the charge transfer mechanism shown in Figure 3a because of the use of VOSO_4_ salt for preparing electrolytes in academic literature. The charge transfer process can be described as follows: i) [V(H_2_O)_5_SO_4_]^+^ reaches the electrode surface and interacts with the adsorbed *OH forming an adsorbed bridged intermediate, *[O─V(H_2_O)_5_SO_4_]; ii) This *[O─V(H_2_O)_5_SO_4_] bridged intermediate undergoes an electron transfer and loses SO_4_^2−^ to form adsorbed *[O─V(H_2_O)_5_]^+^; iii) The adsorbed *[O─V(H_2_O)_5_]^+^ complex desorbs, taking a water molecule from the solvent and forming [V(H_2_O)_6_]^2+^, leaving the electrode surface regenerated. The loss of SO_4_^2−^ anion from the coordination sphere of V^2+^ can also occur directly in solution, instead of when V^2+^ is adsorbed at the electrode surface.^[^ ^26^, ^60^, ^61^ ^]^

The VO^2+^/VO_2_^+^ reaction on the positive half of VRFB is also proposed to involve the formation of an adsorbed intermediate with *OH as the bridge (Figure 3b). V^4+^ exists as [VO(H_2_O)_5_]^2+^,^[^ ^60^, ^61^, ^62^ ^]^ and V^5+^ exists as a mixture of [OVO(H_2_O)_4_]^+^ and [OVO(H_2_O)_3_]^+^ in aqueous H_2_SO_4_.^[^ ^25^, ^60^, ^61^, ^63^, ^64^, ^65^, ^66^, ^67^ ^]^ We propose a reaction mechanism assuming the dominant product is [OVO(H_2_O)_4_]^+^. The [VO(H_2_O)_5_]^2+^ moiety undergoes an ion exchange with the protons on the functional groups of the electrode and forms an adsorbed intermediate *[O─VO(H_2_O)_4_]^+^. The postulated intermediate has an overall +1 charge due to the H^+^ formed during the adsorption process. This adsorbed intermediate undergoes an electron transfer to form adsorbed V^5+^ complex *[O─VO_2_(H_2_O)_3_]. This adsorbed V^5+^ complex desorbs from the surface and takes a water from the solvent to form [OVO(H_2_O)_4_]^+^.^[^ ^53^, ^56^, ^57^, ^58^, ^59^ ^]^

Even though these mechanisms are often able to describe the experimental observations for the vanadium redox couples used in VRFBs, more targeted work to understand the effect of specific functional groups on the electrode surface is needed to better understand these reactions and the effect of the treatments.

## 3 Identifying the Most Effective Electrode Treatments and Electrocatalysts for VRFBs

CFs used as electrodes in VRFBs are often treated or loaded with electrocatalysts to improve the overall battery performance by reducing the overvoltages. More than 50 treatments and 20 electrocatalysts for VRFBs have been reported to improve RFB performance; however, there is no comparison of which treatment or electrocatalyst provides the maximum improvement. We attempt to compare these treatments and electrocatalysts under similar operating conditions based on their performance, stability, and economic feasibility.

Various CF treatments have shown to improve the kinetics of vanadium redox couples on the positive and negative halves and improvement in overall VRFB performance. Here, we classify the treatments in various categories for CF treatments, verify which half of the battery is improved kinetically (superscript “*” = positive half (VO_2_^+^/VO^2+^), superscript “^” = negative half (V^2+^/V^3+^), and superscript “#” = both positive and negative half), and whether both full‐ and half‐cell studies are conducted (superscript “a” = only full‐cell and no half‐cell studies, superscript “b” = only half‐cell and no full‐cell studies, no superscript = both half‐ and full‐cell studies) that show the improvement in performance and kinetics respectively.

1. Acid/Base Treatments: H_2_SO_4_,^#^,^[^ ^68^ ^]^ HNO_3_,^#^,^[^ ^68^ ^]^ Aqua Regia,^#^,^[^ ^54^ ^]^ KOH*^a^*.^[^ ^6^ ^]^
2. Thermal and Gas Treatment: Air,^#^,^[^ ^56^, ^69^, ^70^, ^71^, ^72^, ^73^, ^74^, ^75^ ^]^ O_3_,^#^,^[^ ^72^ ^]^ CO_2_,^*^,^[^ ^76^ ^]^ Ar*^a^*,^[^ ^77^ ^]^
3. Plasma Treatment: N_2_,^#^,^[^ ^78^, ^79^ ^]^ O_2_,^#^,^[^ ^70^, ^71^, ^78^, ^80^ ^]^ Ar^#^,^[^ ^78^ ^]^
4. Electrochemical Oxidation: Oxidative potential,^#^,^[^ ^57^, ^81^ ^]^ Square wave pulse^#^,^[^ ^53^ ^]^
5. Microwave^*^,^[^ ^82^ ^]^ and Gamma Ray*^a^*,^[^ ^70^ ^]^ Treatment
6. Corona Discharge,^#^,^[^ ^83^ ^]^ H_2_O_2_,^#^,^[^ ^80^, ^83^, ^84^, ^85^ ^]^ HF^*^,^[^ ^84^ ^]^
7. Doped with Elements: N,^#^,^[^ ^86^, ^87^, ^88^, ^89^, ^90^, ^91^, ^92^, ^93^, ^94^, ^95^, ^96^, ^97^ ^]^ B,^#^,^[^ ^73^ ^]^ O,^#^,^[^ ^73^, ^92^ ^]^ S,^#^,^[^ ^93^, ^95^, ^98^ ^]^ P,^#^,^[^ ^77^, ^89^, ^99^ ^]^ and Halogens (Cl, Br, I)^#^,^[^ ^100^ ^]^
8. Porous Electrodes by Chemical Reaction: NiO/Ni,^#^,^[^ ^101^ ^]^ K_2_FeO_4_^^^, ^[^ ^102^ ^]^
9. Carbon‐based Electrocatalysts: Graphite oxide nanoplatelets,^#^,^[^ ^100^, ^103^ ^]^ Graphite‐ carbon nanotubes (CNTs),^#^ Single‐walled CNTs (SWCNTs),^#^,^[^ ^104^ ^]^ Multi‐walled CNTs (MWCNTs),^#^,^[^ ^94^, ^95^, ^96^, ^98^, ^99^, ^105^, ^106^, ^107^, ^108^ ^]^ Carbon dots,^#^,^[^ ^109^ ^]^ Carbon nanofiber/CNT composite,^#^,^[^ ^110^ ^]^ Graphite/Graphite oxide,^#^,^[^ ^111^ ^]^ Carbon black,^#^,^[^ ^88^ ^]^ Carbon Nano‐^#^ and Micro‐spheres,^*^,^[^ ^89^, ^97^ ^]^ Graphite oxide based graphene^*^,^[^ ^111^, ^112^ ^]^

Similar to the CF treatments, several metal and metal oxide based electrocatalysts are loaded on CFs using techniques like wet impregnation, electrodeposition, hydrothermal method and have shown to improve the charge transfer kinetics and performance of VRFBs. Here we summarize all the metals and metal oxide based electrocatalysts tested for VRFBs:

1. Metals: Bi,^#^,^[^ ^113^, ^114^, ^115^, ^116^, ^117^ ^]^ Sn,^#^,^[^ ^118^ ^]^ Sb,^#^,^[^ ^119^ ^]^ Ag,^^^,^[^ ^120^ ^]^ Cu,^^^,^[^ ^121^ ^]^ RuSe,^*^,^[^ ^122^ ^]^ Prussian Blue,^*^,^[^ ^123^ ^]^ Pt,^#,^ *^b^*,^[^ ^124^ ^]^ CuPt_3_,^#,^ *^b^*,^[^ ^125^ ^]^ Ir,^#,^ *^b^*,^[^ ^124^ ^]^ Mn,^#,^ *^b^*,^[^ ^124^ ^]^ Te,^#,^ *^b^*,^[^ ^124^ ^]^ In,^#,^ *^b^*,^[^ ^124^ ^]^ Pd,^#,^ *^b^*,^[^ ^124^ ^]^ Au^#,^ *^b^*,^[^ ^124^ ^]^
2. Metal oxides and others: Nb_2_O_5_,^#^,^[^ ^126^ ^]^ ZrO_2_,^#^,^[^ ^58^ ^]^ Mn_3_O_4_,^#^,^[^ ^127^ ^]^ WO_3_,^#^,^[^ ^128^ ^]^ SnO_2_,^#^,^[^ ^129^ ^]^ CeO_2_,^#^,^[^ ^59^, ^130^ ^]^ NiO,^#^,^[^ ^131^, ^132^ ^]^ Co_3_O_4_,^#^,^[^ ^133^ ^]^ Nd_2_O_3_,^#^,^[^ ^134^ ^]^ MoO_3_,^#^,^[^ ^135^ ^]^ KMnO_4_,^#^,^[^ ^136^ ^]^ TiO_2_,^^^,^[^ ^137^, ^138^ ^]^ H: TiO_2_,^^^,^[^ ^139^ ^]^ IrO_2_,^*^,^[^ ^140^ ^]^ Cr_2_O_3_,^*^,^[^ ^141^ ^]^ CoO,^*^,^[^ ^132^ ^]^ NiCoO_2_,^*^,^[^ ^132^ ^]^ Ta_2_O_5_,^*^,^[^ ^142^ ^]^ PbO_2_,^*^,^[^ ^143^ ^]^ RuO_2_ *^a^*,^[^ ^144^ ^]^

Several other metal nitrides and carbides (TiN, TiC, etc.)^[^ ^145^, ^146^, ^147^, ^148^ ^]^ have also improved the performance of VRFBs, but we limit our analysis to metal and metal oxide electrocatalysts in this review.

### 3.1 Methodology for Comparing Treatments

For comparing across different treatments in VRFBs, we extracted the following information from the literature published:

- Treatment of positive and negative electrode.
- CF source.
- Membrane.
- Size of electrode used in cells.
- Concentration of vanadium ions and electrolyte.
- Charging and discharging current densities.
- Upper and lower limit on voltage during charging and discharging.
- Number of cycles of operation.
- CE, VE, and EE at different current densities.
- Stability of EE.
- Initial and final capacity and capacity fade rate.
- Additives, if any.
- Details of treatment.

The above listed parameters for CF treatments and metal and metal oxide electrocatalysts are compiled together in Tables S1 and S2 (Supporting Information) respectively. We observed that the 6 mm thick polyacrylonitrile (PAN) based CFs are the most common grade of CFs used in VRFBs. Nafion‐based membranes (112, 115, and 117) are typically used and the area of the electrodes ranges from 4–25 cm^2^. The total vanadium ion concentration ranges from 1.3–1.7 M, while the acid is 2.5–3.5 M H_2_SO_4_. The upper limit on charging generally ranges from 1.5–1.7 V, while the lower limit on discharging ranges from 0.8–1.0 V. The stability is evaluated for only those treatments that involves operation and reports EE for at least 50 cycles. We observe that the initial and final capacities are reported for very few treatments, due to which we could not use the capacity fade rate in our analysis of treatments. The complete conditions and the process followed for each of these treatments are available in Tables S3 and S4 (Supporting Information).

Besides the electrode treatment, the membrane, cell design, and charging and discharging voltage limits influence VRFBs performance. However, as we focus herein on the electrode treatments and there is not enough data available if changes in parameters such as membrane and charging/discharging potentials are also considered, we neglect those other parameters. Even though these parameters are not considered in this study, we include them in Tables S1 and S2 (Supporting Information) so that readers can refer to complete experimental conditions when considering treatments. We further only consider VRFBs tested in H_2_SO_4_ to eliminate the impact of other anions in the electrolyte. The presence of chloride and other anions has been shown to impact the kinetics and overall performance of VRFBs.^[^ ^17^, ^20^, ^24^, ^26^ ^]^

We compare different treatments for VRFBs based on three criteria: (a) Performance as seen by EE at different current densities, (b) Stability as seen by a change in EE with increased cycling, and (c) Economic feasibility as seen by the capital investment feasible for treatment. To capture the effect of the electrode treatment, we use the performance of a laboratory scale VRFB with untreated (UT) CFs as a reference case, where other components (e.g., membrane, cell geometry) are held constant. We also consider a scaled‐up VRFB (200 kW, 400 kWh) with untreated (UT/S) CFs reported recently in the reference case because VRFB with UT/S CFs allows to capture the effect of cell size and evaluate whether the improvements in performance in laboratory scale can be translated to VRFBs implemented industrially.^[^ ^149^ ^]^ We discuss each of these metrics in detail in the Sections 3.1.1─3.1.3 below, taking the treatment with ID *Thermal‐2* as an example.

#### 3.1.1 Evaluating Performance

We use EEs at the same current densities to compare the effect of various treatments on the performance of VRFBs. VE, CE, and overall EE are dependent on the operating current density (Figure 2a). The overvoltages reduce VE and if the overvoltages are sufficiently high side reactions such as hydrogen and oxygen evolution can lower CE, thereby further reducing the overall EE. Hence the current density must be kept the same while comparing the efficiencies for various treatments. We observe that the CEs are almost unaffected with treatment for VRFBs (Figure S1, Supporting Information), indicating that the EEs can be used for direct comparison for performance and changes in EE are a direct result of the changes in VE. Since the performance of VRFBs at the laboratory scale might not be the same as for a scaled−up VRFB, we compare absolute EE for VRFBs with treated (T) and UT/S CFs at the same current densities to capture the effect of performance on cell size. Additionally, the CFs, membranes, and other battery components described in literature are purchased from different vendors and are of different grades, which can potentially have a significant impact on performance. Thus, to make fair comparison across treatments and capture the effect of the electrode treatment, only the relative changes in EE between VRFBs with T and UT CFs using battery components of the same grade and vendor are used for comparison.

**Figure 4:**
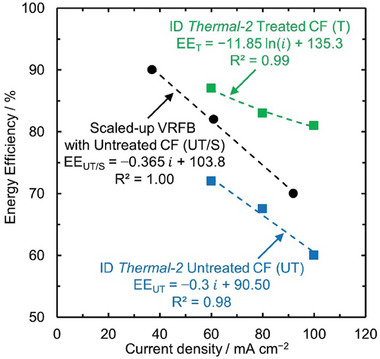
Energy efficiency versus current density (*i*) relationships of VRFB with treatment ID *Thermal‐2* with treated (T) and untreated CFs (UT) along with scaled‐up VRFB (200 kW/ 400 kWh) with untreated CFs (UT/S). Trendlines fitting the data are shown by dotted lines. R^2^ is the coefficient of determination. The current densities and energy efficiencies of VRFB with treatment ID *Thermal‐2* with T and UT CFs is extracted from reference^[^ ^69^ ^]^ and scaled‐up VRFB (200 kW/ 400 kWh) with UT/S CFs is extracted from reference.^[^ ^149^ ^]^

To use the performance data available at different current densities for different treatments, we developed mathematical relations between the current densities and EE for VRFBs with T and UT CFs to evaluate performance at the same current densities for all treatments. The mathematical relations are unique to each VRFB with T and UT CFs corresponding to each treatment ID. Since all the experimental conditions and battery component sources are the same for VRFB with T and UT CFs for each treatment ID, these mathematical relations capture the effect of electrode treatments for each treatment ID. To identify the nature of these mathematical relations, we evaluated the dimensionless Wagner number (*W_a_*), which is the ratio of kinetic to solution resistance. For the majority of the treatments considered, *W_a_* ∼ 1, indicating that the full‐cell measurements in studies were conducted under ohmic or kinetic regime. Consequently, these mathematical relations are chosen to be either linear (based on Ohm's law) or exponential (based on Butler‐Volmer kinetics),^[^ ^150^, ^151^ ^]^ depending on the function that gives the best fit to the experimental data based on the value of the coefficient of determination (R^2^). **Figure** **4** shows the mathematical relations for VRFB with ID *Thermal‐2* (using both treated and untreated CFs) and VRFB with UT/S CFs. We evaluate the EE at three current densities for all treatments that are selected based on the most commonly used current densities in literature and compare them to the absolute EE of VRFB with T CFs and to VRFB with UT/S CFs. We also look at the relative change in EE of VRFB with T CFs compared to UT CFs at these three selected current densities.

For a treatment to satisfy our performance criteria, the following standards must be met:

- Relative change in EE for VRFB with T and UT CFs should be greater than a nominal value of 5% for at least one of the three current densities chosen for comparison.
- Absolute EE for VRFB with T CFs should be more than the EE of VRFB with UT/S CFs operating at same current density. This should be true for at least one of the three current densities chosen for comparison.

A treatment's performance is marked as GREEN if both (a) and (b) standards are satisfied and RED if none of the standards are satisfied. If either of the standards is satisfied and information pertaining to the other is unavailable, the performance is marked as GRAY suggesting more information is needed to evaluate the performance of the treatment.

**Figure 5:**
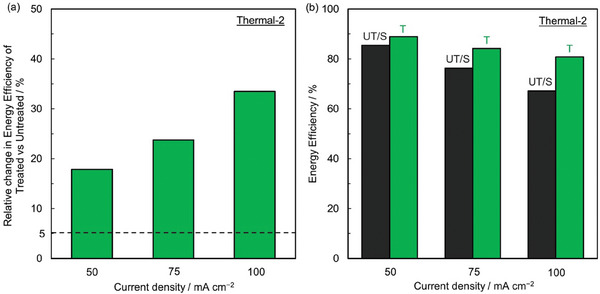
a) Relative change in EE of VRFB using treated (T) CFs with treatment ID *Thermal‐2* with respect to untreated (UT) CFs at operating current densities of 50, 75, and 100 mA cm^−2^. The dotted line shows the nominal 5% change required to satisfy the criteria. b) Absolute energy efficiency (EE) of VRFB with CFs with treatment ID *Thermal‐2* and scaled‐up VRFB (200 kW/ 400 kWh) with untreated CFs (UT/S) at different operating current densities of 50, 75, and 100 mA cm^−2^. The raw current densities and energy efficiencies used to obtain performance parameters at selected current densities of 50, 75, and 100 mA cm^−2^ of VRFB with treatment ID *Thermal‐2* with T and UT CFs is taken from reference^[^ ^69^ ^]^ and scaled‐up VRFB (200 kW/ 400 kWh) with UT/S CFs is taken from reference.^[^ ^149^ ^]^

We take the example of treatment with ID *Thermal‐2* below and evaluate whether *Thermal‐2* satisfies the performance criteria (**Figure** **5**). The EE of VRFBs with T CFs using *Thermal‐2* treatment is 88.97, 84.17, and 80.76 at 50, 75, and 100 mA cm^−2^, respectively. EEs of VRFB with UT CFs is 75.50, 68.00, and 60.50% and VRFB with UT/S CFs is 85.44, 76.32, and 67.19% at 50, 75, and 100 mA cm^−2^, respectively evaluated using the mathematical relations developed in Figure 4. Therefore, the relative change in EE between VRFBs with T and UT CFs is 17.84, 23.78, and 33.48% at 50, 75, and 100 mA cm^−2^, respectively which is greater than 5% at all evaluated current densities as shown in Figure 5a. The absolute EEs of VRFB with T CFs are greater than EEs of VRFB with UT/S CFs at all three current densities used for comparison as shown in Figure 5b. Since both of our performance criteria (a) and (b) are satisfied, the ID *Thermal‐2* is marked as GREEN for performance.

#### 3.1.2 Evaluating Stability of Electrodes after Treatment

RFBs are desired to operate for long durations (> 20 years, > 10 000 cycles).^[^ ^152^ ^]^ If the EE reduces significantly over a short duration of operation, the battery will not store and deliver energy efficiently. The reduction in EE with operation can arise due to the electrode degradation, electrolyte imbalance, or membrane decay. Consequently, there will be a need for electrode replacement, electrolyte rebalancing, or membrane regeneration, which will introduce additional capital and operational costs.

The stability yielded by a treatment is associated with how the performance of the RFB changes over the lifetime of operation. We evaluate the EE degradation factor (% cycle^−1^) which captures how the EE evolves with the number of operating cycles. We assume that electrode degradation is the primary cause of EE degradation. We only consider treatments for which performance is evaluated for at least 50 cycles of operation. According to the Department of Energy (DOE), the EE degradation factor is < 0.0012% cycle^−1^ for a VRFB with UT/S CFs.^[^ ^153^, ^154^ ^]^ Thus, EE degradation factor for VRFB with treated CFs should be < 0.0012% cycle^−1^ to compete with VRFB with UT/S CFs and satisfy the stability criteria.

The stability of a treatment is marked as GREEN or RED if the criteria is satisfied or not satisfied respectively. A treatment is marked as GRAY if the performance data for at least 50 cycles is unavailable to conduct stability analysis. For treatment with ID *Thermal‐2*, the EE changes from 83.0 to 82.9% over 60 cycles for an operating current density of 80 mA cm^−2^. Thus, EE degradation factor is 0.0017% cycle^−1^ which exceeds the DOE limit of 0.0012% cycle^−1^. Resultantly, *Thermal‐2* treatment does not satisfy the stability criteria, and hence is marked as RED.

#### 3.1.3 Economic Feasibility of Electrode Treatment

Even though the treatment might improve performance for the entire lifetime of the VRFB, an estimate of the maximum allowed electrode treatment cost is essential. We evaluate a parameter Affordable Capital Cost (ACC) which is defined as the maximum amount of capital investment ($ m^−2^ of electrode surface) that can be done in a treatment without causing an increase in the overall capital expenditure (CAPEX) of VRFB with UT CFs. A treatment with a higher ACC is more economical.

We develop a techno‐economic model to evaluate the total CAPEX. Here we consider the base case of a VRFB delivering 1.5 MW of power for 8 h discharge duration with capacity utilization factor of 80% and operating at EE of 67.3%. These choices of total power, discharge duration, capacity utilization factor, and EE are in alignment with the DOE targets for long‐duration energy storage technologies.^[^ ^153^, ^154^ ^]^ The total CAPEX has contributions from battery stack, balance of plant hardware and electrical system, electrolyte, and utilities. We do not consider the cost associated with balance of plant electrical system, electrolyte, and utilities to evaluate the total CAPEX since these portions of CAPEX are unchanged for an RFB with fixed energy capacity and do not affect the calculated ACC. More details of the techno‐economic model are available in Tables S5 and S6 (Supporting Information).

**Figure 6:**
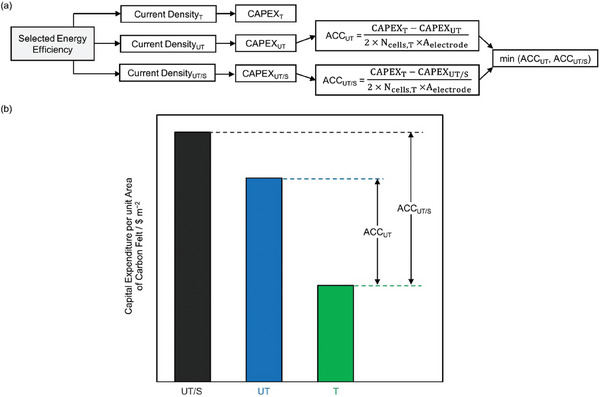
a) Flowchart showing the steps followed to evaluate the affordable capital cost (ACC). Here N_Cells, T_ is the total number of cells needed to deliver the rated power for VRFB with treated CFs and A_electrode_ is the area of electrode used on each side of the battery. b) Example plot showing the capital expenditure per unit area of electrode for VRFBs with UT/S, UT, and T CFs used to evaluate ACC_UT/S_ and ACC_UT_ at a selected energy efficiency.

We evaluate the ACC at three different EEs (67.3, 73, and 78%) to span over the range of operating current densities. ACC is calculated by following the steps shown in **Figure** **6**a. For a selected EE, the operating current densities for VRFB with T, UT, and UT/S CFs are calculated from the EE versus current density relationships developed for evaluating performance in Section 3.1.1. Due to the change in operating current density for VRFBs with treated and untreated CFs to deliver the rated power, a different number of stacks are required for each case. Resultantly, the CAPEX contributions from battery stack and balance of plant hardware component are different for both cases. ACC is calculated by considering the reduction in CAPEX per unit area of CFs required in VRFB with treated CFs at laboratory scale compared to VRFB with untreated CFs at both laboratory and industrial scale (ACC_UT_ and ACC_UT/S_) as shown in Figure 6b.

For a given treatment, if the value of ACC, i.e., min(ACC_UT_, ACC_UT/S_) is greater than 300 $ m^−2^ (ACC_min_) for at least one of the operating EEs, then we consider the treatment to have satisfied the economic feasibility criteria. The selection of min(ACC_UT_, ACC_UT/S_) to calculate ACC allows to account for the worst among the laboratory and scaled‐up VRFB. For instance, in Figure 6b, ACC is equal to ACC_UT_ since ACC_UT_ is lower compared to ACC_UT/S._ The ACC_min_ is chosen to be 1.5 times the cost of commercially available thermally treated CF (Figure S10 and Section S3, Supporting Information). The cost of thermally treated CF is used to identify ACC_min_ because thermal treatment involves a single step and is already implemented at the industrial scale for various operations in fuel cell and biomass conversion.^[^ ^155^ ^]^ Additionally, thermal treatment is one of the first treatments proposed for CFs for RFBs, due to which several companies (Cera Materials, SGL Carbon, etc.)^[^ ^156^, ^157^ ^]^ have started selling thermally treated CFs.

**Figure 7:**
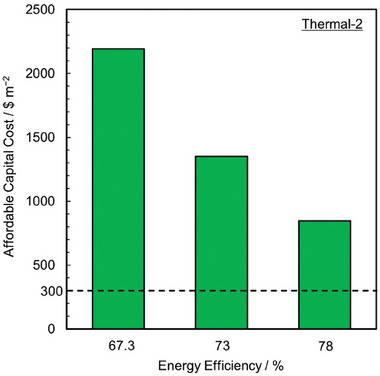
Affordable capital cost for VRFB with ID *Thermal‐2* at energy efficiencies of 67.3, 73, and 78%. The dotted line indicates the minimum value of ACC required for a treatment to satisfy the criteria. The ACC for VRFB with ID *Thermal‐2* at selected energy efficiencies of 67.3, 73, and 78% utilizes the current densities and energy efficiencies relationships developed using performance data obtained for VRFB with treatment ID *Thermal‐2* with T and UT CFs from reference^[^ ^69^ ^]^ and scaled‐up VRFB (200 kW/ 400 kWh) with UT/S CFs from reference.^[^ ^149^ ^]^

A treatment's economic feasibility is marked as GREEN if the treatment satisfies the economic feasibility criteria for at least one of the operating EEs and as RED if the treatment does not satisfy the economic feasibility criteria for all three operating EEs. The treatment's economic feasibility is marked as GRAY if insufficient information is available to evaluate ACC. For treatment with ID *Thermal‐2*, the current densities for VRFBs with T CFs are 311, 194, and 128 mA cm^−2^ and UT CFs are 77, 59, and 42 mA cm^−2^ for EEs of 67.3, 73, and 78%, respectively. Similarly, VRFB with UT/S CFs operates at 100, 84, and 71 mA cm^−2^ for the 67.3, 73, and 78% EE, respectively. These current densities are evaluated from the mathematical relations between current densities and EE developed in Section 3.1.1. Using the methodology described above in Figure 6, ACC_UT_ and ACC_UT/S_ are evaluated and min(ACC_UT_, ACC_UT/S_) is shown in **Figure** **7**. Clearly, min(ACC_UT_, ACC_UT/S_) > 300 $ m^−2^ at all operating EEs, making treatment with ID *Thermal‐2* economically feasible and is therefore marked as GREEN.

### 3.2 Comparison Among Different Carbon Felt Treatments and Metal and Metal Oxide Electrocatalysts

**Figure 8:**
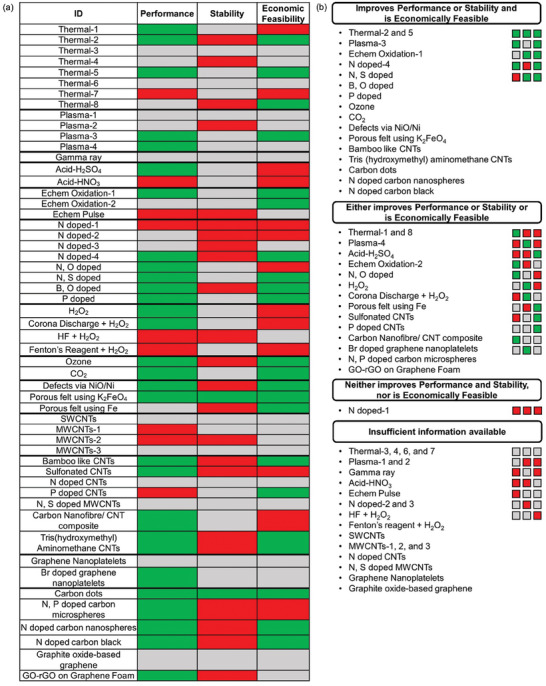
a) Carbon felt treatments that satisfy (GREEN) or do not satisfy (RED) the performance, stability, and economic feasibility criteria. Inadequate information for a treatment is shown by GRAY color. See Table S1 (Supporting Information) for ID information and text for criteria and b) Carbon felts treatments sorted in various categories based on whether they satisfy the criteria for performance, stability, and economic feasibility. The allowed color code combinations for a treatment to belong to a particular category is shown by colored squares on the right of each category.

**Figure 9:**
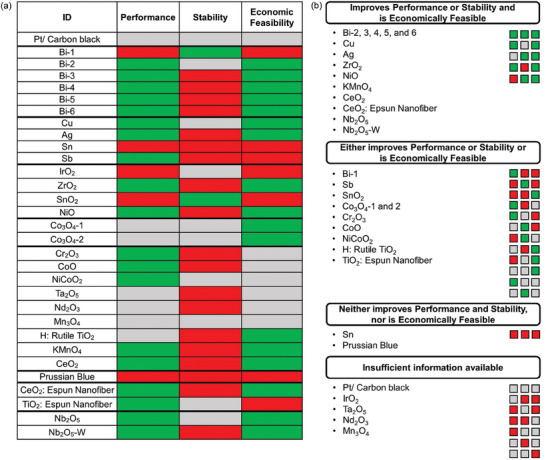
a) Metal and metal oxide electrocatalysts that satisfy (GREEN) or do not satisfy (RED) the performance, stability, and economic feasibility criteria. Inadequate information is shown by GRAY color. See Table S2 (Supporting Information) for ID information and text for criteria and b) Metal and metal oxide electrocatalysts sorted in various categories based on whether they satisfy the criteria for performance, stability, and economic feasibility. The allowed color code combinations for a treatment to belong to a particular category is shown by colored squares on the right of each category.

Using the same methodology as described in Section 3.1 for treatment with ID *Thermal‐2*, we mark each of the treatments as GREEN, RED, or GRAY for performance, stability, and economic feasibility (**Figures** **8**a and **9**a). The absolute EE and relative change in EE at three different current densities for CF treatments and metal and metal oxide electrocatalysts is shown in Figures S2−S5 (Supporting Information). Figures S2−S5 (Supporting Information) can also be used to compare performance of treatments at a selected current density. EE degradation factor is shown in Figures S6 and S7 (Supporting Information) and can be used to compare stability of all treatments. Many of the treatments do not report EE data for at least 50 cycles and so we cannot conduct stability analysis for these treatments (denoted by GRAY under stability in Figures 8a and 9a). This lack of stability information indicates the need for performance data over a greater number of cycles in future research. ACC at different operating EEs is shown in Figures S8 and S9 (Supporting Information) for CF treatments and metal and metal oxide electrocatalysts respectively. Figures S8 and S9 (Supporting Information) can also be used to compare the economic feasibility of all treatments at a selected energy efficiency.

We classify the treatments in four different categories based on whether they satisfy the performance, stability, and economic feasibility criteria (Figures 8b and 9b):

- Improves performance or stability and is economically feasible.
- Either improves performance or stability or is economically feasible.
- Neither improves performance and stability, nor is economically feasible.
- Insufficient information available.

Several CF treatments improve VRFBs performance or stability and are economically feasible and therefore are promising for industrial implementation as shown in Figure 8. These promising treatments generally belong to above‐defined categories of thermal and gas, plasma, doping with elements, porous electrodes by chemical reaction, and carbon electrocatalysts based on micro (or nano) spheres. Carbon electrocatalysts based on nanoplatelets, composites, doped carbon nanotubes (CNTs) either just satisfy the performance or stability criteria or are just economically feasible. There is not enough information available to comment on most CF treatments using single and multi‐walled CNTs. Treatments with ID *Porous felt using K_2_FeO_4_* and *Carbon dots* are the only CF treatments that satisfy the stability criteria indicating the need for increased focus on improving the long‐term stability of electrodes after treatments.

Several metals, doped metal oxides, and electrospun nanofibers show great promise because of their good performance, stability, and economic feasibility as shown in Figure 9. All metals excluding *Sn* and *Sb*, several metal oxides like *ZrO_2_*, *NiO*, doped metal oxides like *Nb_2_O_5_‐W*, and electrospun nanofibers satisfy the performance or stability criteria and are economically feasible. A large proportion of metal oxides have insufficient data, or either satisfy only one of the performance, stability, or economically feasibility criteria. Similar to CF treatments, most of the metal and metal oxide electrocatalysts do not satisfy the stability criteria.

## 4 Complexity of Treatments for Industrial Implementation

**Figure 10:**
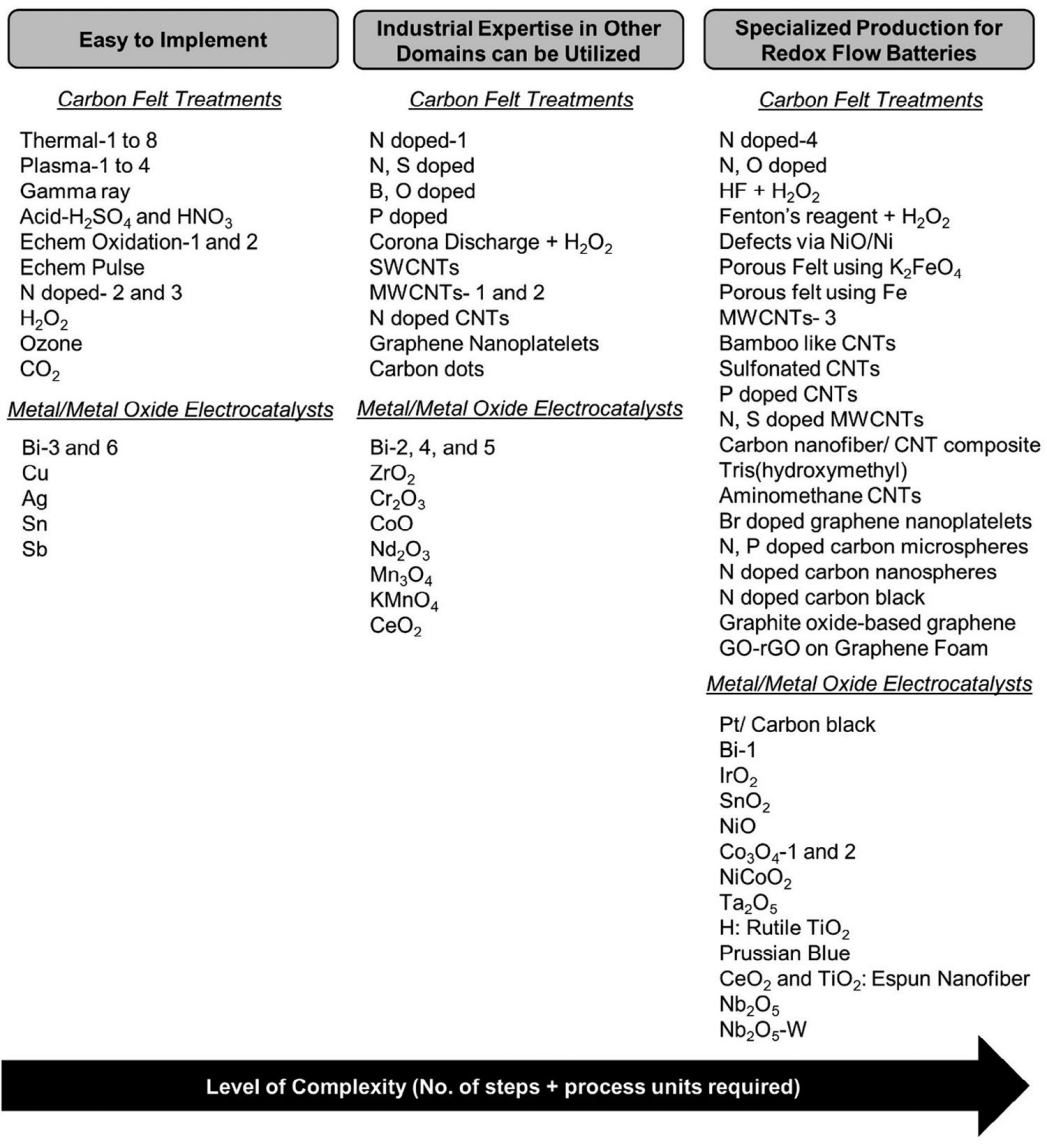
Carbon felt treatments and metal and metal oxide electrocatalysts grouped based on their level of complexity. The treatment complexity is the number of steps in the process and process units required to implement at the industrial scale. The total number and details of steps and process units needed for each treatment are available in Tables S3 and S4 (Supporting Information).

In this Section, we further categorize the treatments discussed in Section 3 on the basis of treatment complexity. The treatment complexity arises based on the need of unique process units or requiring multiple steps with quality checks. Based on the details of each treatment available in literature and compiled in Tables S3 and S4 (Supporting Information), we estimate the number of steps and process units needed for each treatment. We use the sum of the number of process units and steps ($N_{Process\;units\;\&\;steps}$) calculated for each treatment in Tables S3 and S4 (Supporting Information) to indicate the treatment complexity and divide the treatments into three broad categories as shown in **Figure** **10**:

1. Easy to implement: These treatments can be implemented industrially based on the current know‐how and industrial setup. Treatments with $N_{Process\;units\;\&\;steps}$ ≤ 6 are assigned to this category.
2. Industrial expertise in other domains can be utilized: These treatments can utilize the know‐how of similar processes used for various applications in industry. Treatments with 7 ≤ $N_{Process\;units\;\&\;steps}$ ≤ 10 are assigned to this category.
3. Specialized production for RFBs: These treatments have not been used commercially till now in any industry to our knowledge and will need specialized production for RFBs. Treatments with $N_{Process\;units\;\&\;steps}$ > 10 are assigned to this category.

Treatments that are easy to implement industrially (Figure 10) are the ones that involve keeping the electrodes in furnaces at high temperature (*Thermal‐1 to 8*, *N doped‐2 and 3*, *Ozone*, *CO_2_*), equipment providing high energy beams (*Plasma‐1 to 4*, *Gamma ray*), treatment in acids (*Acid‐ H_2_SO_4_ and HNO_3_*, *Echem Oxidation‐1 and 2*, *Echem Pulse*, and *H_2_O_2_*), or adding electrocatalysts to the electrolyte directly (*Bi‐3 and 6*, *Cu*, *Ag*, *Sn*, *Sb*). Several of these easy to implement treatments are currently used in preparation of carbon fibers. The capital costs associated with these processes in the carbon fiber industry can be extended to get an estimate of cost of treatments in CFs, as discussed in Section 5.

Several treatments of CFs can be scaled up by utilizing the expertise of similar processes used for different applications in industry, as described below:

- Ozone gas is used to destroy germs in water and implemented commercially in water purification industry.^[^ ^158^, ^159^ ^]^
- Doping of CF used in RFBs is generally done by hydrothermal method (Tables S3 and S4, Supporting Information). Hydrothermal method is also used commercially in the preparation of zeolites and value‐added chemicals from biomass,^[^ ^160^, ^161^, ^162^, ^163^ ^]^ whose experience can be utilized for scaling up the production of doped CFs.
- CNTs are used in supercapacitors due to their unique material properties.^[^ ^164^ ^]^ The supercapacitor industry can help in setting up the supply chain network of CNTs for RFBs. Additionally, doping of CNTs is done by hydrothermal method similar to CFs,^[^ ^165^, ^166^ ^]^ allowing them to draw from the experiences of the zeolite and biomass industry discussed above.^[^ ^160^, ^161^, ^162^, ^163^ ^]^
- Impregnation and dip coating is often used to load catalysts on electrodes for fuel cells.^[^ ^167^, ^168^ ^]^ The industries set up to do these processes for electrodes for fuel cells can easily extend these to CFs if incentives are provided.

A lot of focus in recent years has been on the development of unique carbon based electrocatalysts. These include the preparation of carbon nanospheres, carbon microspheres, CNTs with specific structures, carbon composites, carbon foams, and carbon felts with defects. The preparation of carbon based electrocatalysts is complex, time‐consuming, and require the implementation of new supply chains that can provide these materials with high quality for specialized production for RFBs as shown in Figure 10.

## 5 Cost of Easy‐to‐Implement Treatments That Satisfy Performance, Stability, and Economic Feasibility Criteria

Using the findings from Sections 3 and 4, we identify that the treatments with ID *Thermal‐2* and *5*, *Plasma‐3*, *Echem Oxidation‐1*, *CO_2_*, *Ozone*, *Bi‐3* and *6*, *Cu*, and *Ag* are easy to implement and satisfy the criteria of performance or stability and economic feasibility. We estimate the cost of these treatments (except *Ozone*) by utilizing their similarity with the processes used in preparation of CFs which is discussed below.

**Figure 11:**
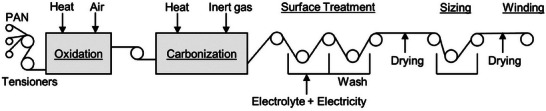
Different stages of making carbon fibers from PAN precursors. Reproduced with permission.^[^ ^169^ ^]^ Copyright 2017, The American Society of Mechanical Engineers.

PAN precursor is the most common raw material used for making carbon fibers. The basic process of obtaining carbon fiber from PAN precursors involves five major stages (**Figure** **11**):^[^ ^169^ ^]^

1. Oxidation: Conducted at 200–300 °C for 1.5–2 h in the presence of air.^[^ ^169^, ^170^ ^]^ In the oxidation step, the linear molecules of PAN‐based polymer precursors are converted into cyclic structures. These cyclic structures have oxygen incorporated in them making them stable to withstand high temperatures encountered in carbonization/graphitization stage.
2. Carbonization or Graphitization: Conducted in inert atmosphere (mostly N_2_) at 800–1000 °C for 15–20 min.^[^ ^169^, ^170^ ^]^ In the carbonization step, due to the high temperatures, non‐carbon materials are removed, which increases the carbon content of the precursor.
3. Surface Treatment: Involves electrochemical oxidation in 0.5–1 M H_2_SO_4_ solution passing 139 mAh g^−1^ (or 500 C g^−1^) of charge.^[^ ^171^ ^]^ The purpose of the surface treatment step is to enhance the adhesion between the matrix resins and carbon fiber to form a reinforced composite.
4. Sizing: A coating is applied to protect and lubricate the fibers in the sizing step.
5. Winding: Involves winding of prepared carbon fibers to prepare rolls.

**Figure 12:**
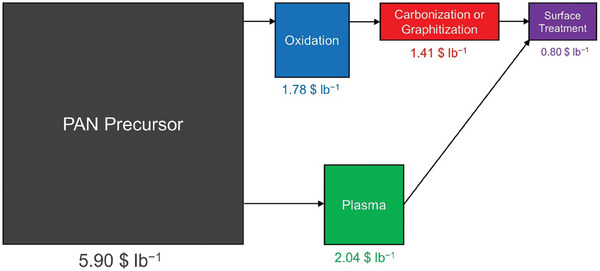
Cost associated with each stage for production of carbon fibers, assuming 1500 ton year^−1^ production capacity. The area of the squares or rectangles and the text size for each of the steps is proportional to their relative costs. The details regarding assumptions are available in references.^[^ ^170^, ^172^, ^173^, ^174^ ^]^

For a 1500 ton year^−1^ carbon fiber production from PAN precursor, researchers at Oak Ridge National Lab reported in 2013 the estimated cost associated with each of the stages, as shown in **Figure** **12**.^[^ ^172^, ^173^, ^174^ ^]^ This cost includes all the CAPEX and operating expenditure (OPEX) contributions over the lifetime of the equipment.^[^ ^170^ ^]^ Additionally, there have been efforts to replace the oxidation and carbonization/graphitization stages with a single plasma treatment stage for 20–24 min.^[^ ^174^ ^]^ The cost of plasma treatment is estimated to be lower compared to both oxidation and carbonization/graphitization stages combined as shown in Figure 12.

### 5.1 Carbon Felts

**Figure 13:**
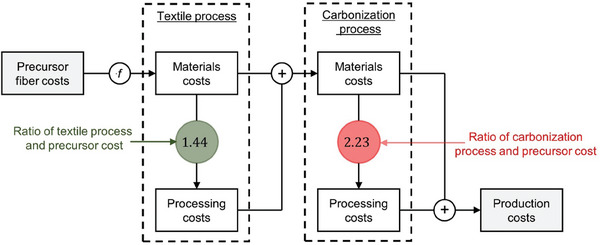
Preparation of carbon felts from PAN precursors. The ratio of cost of textile and carbonization process relative to cost of precursor is shown in green and red colored circles, respectively. Here *f* denotes the cost of the PAN precursor material. Reproduced with permission.^[^ ^175^ ^]^ Copyright 2017, Elsevier.

PAN, used in carbon fibers, is also the precursor for making CFs. Raw CF is produced in a laying and needle punching process, a textile craft for producing felts without the use of water.^[^ ^175^ ^]^ This is followed by the carbonization process similar to the one used in carbon fiber preparation (Figure 11). The yield of the final CF from PAN precursor is ∼50% due to the loss of non‐carbon materials in the carbonization process.^[^ ^175^ ^]^ A previous work in 2017 estimated the cost of textile processing and carbonization process step, relative to the cost of the precursor, shown in **Figure** **13**.^[^ ^175^ ^]^

To estimate the cost of CF, we use the cost of PAN precursor material as 5.9 $ lb^−1^ in 2013,^[^ ^170^ ^]^ and scale it up with a lumpsum inflation of 2%. The textile processing and carbonization process cost is estimated using the ratio of the process costs with the precursor shown in Figure 13. Adding the textile processing and carbonization costs, along with using 500 g m^−2^ as the area weight ratio for a 6 mm thick PAN CF typically used for RFBs and 50% yield of CFs from carbon fibers,^[^ ^175^ ^]^ we estimate the *cost of CF to be ≈67 $ m^−2^*. This cost estimate is close to 77 $ m^−2^, commonly used in literature based on quotes obtained from various vendors.^[^ ^149^ ^]^ More details of the cost calculation of CF are available in Section S3 (Supporting Information).

### 5.2 Thermal, Plasma, Electrochemical Oxidation, and CO_2_ Treatments

We utilize the similarity in the process for making carbon fibers and CFs to estimate costs associated with thermal, plasma, electrochemical oxidation, and CO_2_ treatments. Assuming the production capacity for treated CFs is the same as carbon fibers (i.e., 1500 ton year^−1^), we can obtain a preliminary estimate of cost of these treatments for CFs. A lumpsum inflation of 2% for each year to estimate costs in 2022, a yield of 50% from carbon fiber to CF, and area/weight ratio of 500 g m^−2^ is used while making these estimates.

- *Thermal Treatment*.

VRFB with ID *Thermal‐2* used CFs treated in air at 750 °C for 5 min, while *Thermal‐5* used CFs treated in air at 500 °C for 5 h (Table S3, Supporting Information).^[^ ^69^, ^72^ ^]^ The oxidation stage in carbon fiber preparation occurs between 200–300 °C for 1.5–2 h.^[^ ^169^, ^170^ ^]^ We scale the cost of thermal treatment by the ratio of duty ( = time × temperature) needed for each of these processes (Section S3, Supporting Information). The cost of ID *Thermal‐2* is estimated to be ≈0.9 $ m^−2^, while *Thermal‐5* is estimated to be ≈35.6 $ m^−2^. This cost of *Thermal‐5* is very close to the difference (≈35 $ m^−2^) between the thermally treated and untreated CF sold by SGL carbon (Section S3, Supporting Information).

- *Plasma Treatment*.

CFs used in VRFB with ID *Plasma‐3* is treated for 10 min (Table S3, Supporting Information),^[^ ^78^ ^]^ while carbon fibers are treated in plasma ≈20–24 min.^[^ ^174^ ^]^ We exclude the additional energy cost by scaling the cost down based on the time of treatment (Section S3, Supporting Information). We estimate the *Plasma‐3* treatment to cost ≈2.4 $ m^−2^.

- *Electrochemical Oxidation*.

CFs used in VRFBs with ID *Echem Oxidation‐*1 are electrochemically oxidized in 1 m H_2_SO_4_ passing ≈2050 C g^−1^ of charge (Table S3, Supporting Information),^[^ ^57^ ^]^ contrary to carbon fibers where 500 C g^−1^ is passed in similar H_2_SO_4_ concentration.^[^ ^171^ ^]^ A recent study provided an estimate of the split of total cost into consumables and energy cost for the electrochemical oxidation for carbon fibers.^[^ ^169^ ^]^ The consumable cost is associated with the cost of H_2_SO_4_ required for the treatment and the energy costs account for the electricity costs passing the desired amount of charge. Since the consumable costs remain the same for both CF and carbon fiber treatment, we scaled the electricity cost by the ratio of the charge passed to estimate the cost of electrochemical oxidation of CFs (Section S3, Supporting Information). The cost of electrochemical oxidation is estimated to be ≈8.4 $ m^−2^.

- *CO_2_ Treatment*

CFs used in VRFBs with ID *CO_2_* is conducted at 1000 °C for 0.5 h (Table S3, Supporting Information).^[^ ^76^ ^]^ Assuming that the same furnace used for thermal oxidation (in air) can work for CO_2_ treatment, we can estimate the cost of CO_2_ treatment by accounting the change in duty and cost of gas (Section S3, Supporting Information). The duty also accounts for change in the heat capacity between air and CO_2_. The cost of CO_2_ treatment is estimated to be ≈10 $ m^−2^.

### 5.3 Bi, Ag, and Cu Metal Electrocatalysts

Metal salts are often directly dissolved into the electrolytes in VRFBs (Table S4, Supporting Information). During charging of the battery, the metal cations are reduced, and metals are deposited on the surface of CFs. These deposited metals act as electrocatalyst for charge transfer at the electrode surface leading to an improved performance. Since there are no additional process units and just one extra step required to load these electrocatalysts on CFs, the only capital costs associated are the cost of the metal salts that are added to the electrolyte to achieve the desired loading (kg m^−2^ of electrode). The costs of the metal electrocatalysts for VRFBs is estimated using the following process:

1. Optimized amount of metal salts (g) needed for a fixed electrode size is obtained from literature. Assuming 100% CE, optimized metal loading on the electrode (kg m^−2^) is estimated.
2. Upper limit on cost of metal salts purchased in bulk is used to estimate the cost associated with electrocatalyst loading ($ m^−2^).

**Figure 14:**
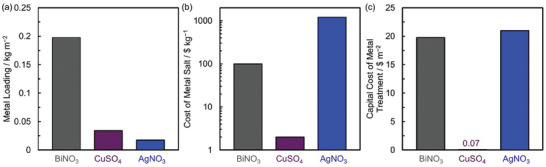
a) Metal loading and b) Cost of metal salt used for various metal electrocatalysts in RFBs, and c) Total capital cost associated with loading these metal electrocatalysts per unit area of carbon felts. The metal loading for Bi, Cu, and Ag is obtained from references,^[^ ^176^ ^]^,^[^ ^121^ ^]^ and^[^ ^120^ ^]^ respectively. The costs of the metal salts have been obtained from vendors that can supply these in bulk for meeting industrial requirements.

The cost of introducing Cu metal in electrolyte is lower than Bi and Ag. **Figure** **14**a shows the metal loading obtained from the literature for treatment IDs *Bi‐3*, *Cu*, and *Ag*.^[^ ^117^, ^120^, ^121^ ^]^ Bi requires a higher metal loading in comparison to Cu and Ag. The cost of metal salts is shown in Figure 14b. CuSO_4_ is the cheapest, followed by BiNO_3_ and AgNO_3_. Resultantly, due to the low metal loading of Cu and low cost of CuSO_4_, Cu requires the least capital cost per unit area of the CF (0.07 $ m^−2^). The high costs of AgNO_3_ lead to the overall cost of Ag being comparable to Bi despite the low metal loading (Figure 14c).

**Figure 15:**
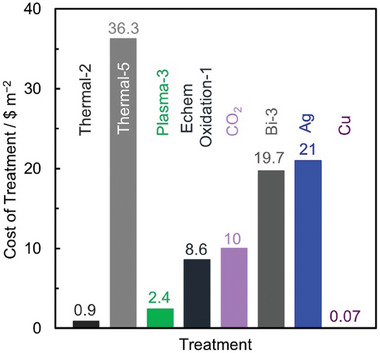
Total capital cost associated with treatments for VRFBs that improve performance or stability, is economically feasible, and are easy to implement.

The estimated total capital costs ($ m^−2^) associated with each of the treatments that are easy to implement and satisfy the criteria of performance, stability and economic feasibility is shown in **Figure** **15**. Clearly, the capital costs for each of these treatments are << ACC_min_ (300 $ m^−2^), indicating that if these treatments for CFs are carried out at the scale of carbon fiber production, these treatments can become very attractive to implement at the industrial scale. The capital costs of treatments follow the order: *Cu* < *Thermal‐2* < *Plasma‐3* < *Echem Oxidation‐1* < *CO_2_* < *Bi‐3* < *Ag* < *Thermal‐5*. This indicates that thermal treatment at lower temperature for longer duration is costlier (*Thermal‐2* vs *Thermal‐5*). Additionally, Cu is the cheapest metal electrocatalyst followed by Bi and Ag. *Plasma‐3*, *Echem Oxidation‐1*, and *CO_2_* treatments are also cost effective and can be implemented industrially.

VRFB with thermally treated and Bi deposited CFs as electrodes are shown to operate at highest current densities of 1000 mA cm^−2^ with peak power of 2.78 W cm^−2^, the maximum reported till date. At current density of 600 mA cm^−2^ operating over 20000 cycles, the EE of this VRFB is reported to be ≈80%.^[^ ^177^ ^]^ From this recent study in which Bi is combined with thermal treatment and our analysis of different treatments conducted in this work, we estimate that the benefits in the performance, stability, and economic feasibility are likely to be improved for a combination of Cu electrocatalyst with thermally, plasma, CO_2_ treated or electrochemically oxidized CF. However, there is no study to the best of our knowledge that has looked at the performance of VRFBs for these combinations of treatments.

## 6 Similarity in Electrode Treatments of AORFBs and VRFBs

**Figure 16:**
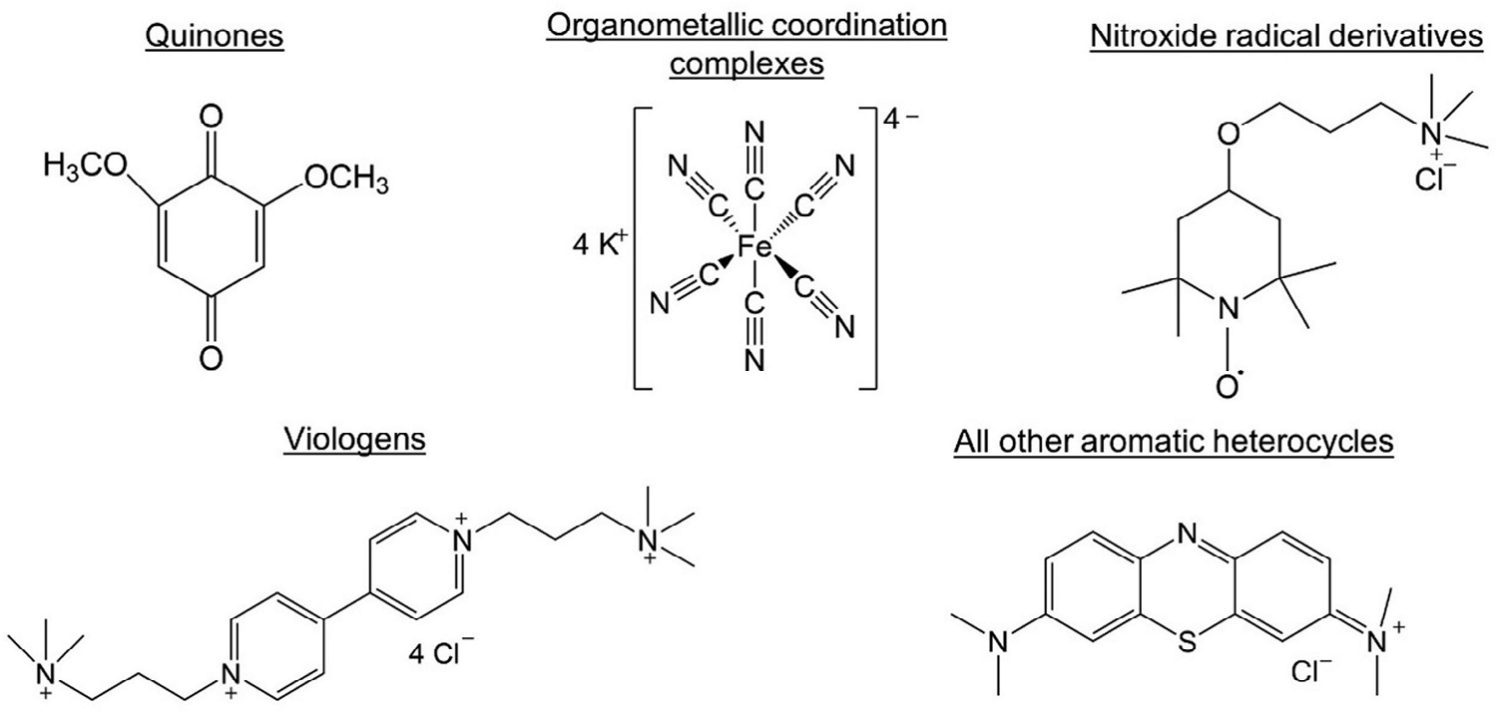
Classes of organic molecules used in aqueous‐organic redox flow batteries based on their structure. A representative molecule for each class is shown, DMBQ for quinones, K_4_Fe(CN)_6_ for organometallic coordination complexes, TMAP‐TEMPO for nitroxide radical derivatives, BTMAP‐Vi for viologens, and MB for all other aromatic heterocycles. Here DMBQ = 2,6‐dimethoxybenzoquinone, K_4_[Fe(CN)_6_] = Potassium hexacyanoferrate, TMAP‐TEMPO = 4‐[3‐(trimethylammonio)propoxy]−2,2,6,6‐tetramethylpiperidin‐1‐oxyl, BTMAP‐Vi = bis(3‐trimethylammonio)propyl viologen tetrachloride, and MB = Methylene Blue.

The high cost of vanadium, instability in the vanadium supply chain, and relatively low energy density of VRFBs have led to the search for alternative RFB chemistries.^[^ ^178^ ^]^ Aqueous‐organic RFBs (AORFBs) have received attention recently due to the use of potentially cheap organic molecules as active species in aqueous solutions. The structure of these organic molecules can be tailored to improve their solubility and undergo multiple charge transfers to increase the battery capacity. Depending on the structure of the redox active species, AORFBs can operate over the entire pH range, contrary to VRFBs which operate only under acidic environments.^[^ ^18^ ^]^ The active species used in AORFBs are classified in five broad classes based on their molecular structure (**Figure** **16**). Unfortunately, most of these organic molecules degrade irreversibly slowly lowering the battery efficiency. Therefore, research over the last decade has been conducted to understand these degradation mechanisms.^[^ ^179^, ^180^ ^]^

AORFBs are generally operated in non‐symmetric condition. Quinones and organometallic coordination complexes are the most popular class of organic molecules in the negative and positive half of AORFBs, respectively (**Table** **1**). CFs and carbon papers are the most commonly used electrodes where organic molecules lose or gain electrons to store and release energy. The use of different derivatives of organic molecules within a class affects the crossover of active species, degradation of molecule, and charge discharge mechanisms, making the comparisons across different studies in literature more challenging. Additionally, the use of CFs and carbon papers from different vendors as electrodes also affects the performance similar to VRFBs. Consequently, the effects of treatments in AORFBs cannot be compared in the same way based on performance, stability, and economic feasibility as conducted for VRFBs in this study. Further, there is not enough data in the AORFB literature to compare performance across treatments. Therefore, in this study, we focus on identifying the electrode treatments employed in AORFBs and their similarities with VRFBs, that can be used to direct future research in this field.

**Table 1:** Treatments for electrodes used in AORFBs tested in half‐ or full‐cell configurations using different organic molecules. “NT” indicates no molecule has been tested for that class to our knowledge.

Treatment	Quinones	Organometallic Coordination	Nitroxide Radical Derivatives	Viologens	All other aromatics
**Full Cell**					
Thermal^a)^,^[^ ^181^, ^182^, ^183^, ^184^, ^185^, ^186^, ^187^, ^188^, ^189^, ^190^, ^191^, ^192^, ^193^ ^]^	AQS,^b^ ^)^ AQDS,^b^ ^)^ JN,^b^ ^)^ NN,^b^ ^)^ ARS,^b^ ^)^ DHAQ,^b^ ^)^ DHAQDS, DHAQDMS, DBEAQ, DPPEAQ, DHBQ, Bislawsone, TMHQ	K_4_[Fe(CN)_6_], BTMAP‐Fc	NT	MV, BTMAP‐Vi	NT
Thermal + KOH Etching^a)^,^[^ ^194^ ^]^	HCNQ	K_4_[Fe(CN)_6_], BTMAP‐Fc	NT	NT	NT
Acid Treatment (Sulfuric + Nitric acid)^a)^,^[^ ^187^, ^188^, ^193^, ^195^, ^196^, ^197^ ^]^	AQDS,^b^ ^)^ AQS, AR	NT	NT	NT	NT
Acid Treatment (Sulfuric acid)^a)^,^[^ ^188^, ^198^ ^]^	AQDS, DHDMBS, AR	K_4_[Fe(CN)_6_]	NT	NT	NT
Plasma^a)^,^[^ ^199^, ^200^ ^]^	DBEAQ,^b^ ^)^ AQDS	K_4_[Fe(CN)_6_]^b^ ^)^	NT	NT	Tiron^b^
N, O‐doping by chemical etching^a)^,^[^ ^201^ ^]^	DHAQ	NT	NT	NT	NT
Carbon black^a)^,^[^ ^202^, ^203^ ^]^	AQDS,^b^ ^)^ BQDS,^b^ ^)^ AQS, DHBQDS	NT	NT	NT	NT
MWCNTs‐COOH^a)^,^[^ ^204^, ^205^ ^]^	NT	K_4_[Fe(CN)_6_]^b^ ^)^	NT		alloxazine‐COOH^b^ ^)^
Reduced Graphene Oxide^a)^,^[^ ^206^ ^]^	NT	NT	4‐HO‐ TEMPO^b^ ^)^	MV^b^ ^)^	NT
N‐doped 3D reduced graphene oxide^a)^,^[^ ^8^ ^]^	NT	NT	4‐HO‐ TEMPO^b^ ^)^	MV^b^ ^)^	NT
Ti_4_O_7_/KB (Ketjenblack)^[^ ^207^ ^]^	FQH_2_ ^b^ ^)^	NT	NT	NT	NT
LaSrO_x_^[^ ^208^ ^]^		NT	4‐HO‐ TEMPO^b^ ^)^	MV^b^ ^)^	NT
Conducting polymers (PEDOT: PSS and PEDOT: Tos)^[^ ^209^ ^]^	ARS^b^ ^)^	NT	NT	NT	Tiron^b^ ^)^
**Half Cell**					
High Edge Density Pyrolitic Graphite^a)^,^[^ ^210^ ^]^	FQH_2_,^b^ ^)^ CQH_2_,^b^ ^)^ BQDS,^b^ ^)^ ARS,^b^ ^)^ frog quinone,^b^ ^)^ crab quinone^b^ ^)^	NT	NT	NT	NT
N doping^a)^,^[^ ^187^, ^188^ ^]^	AQS,^b^ ^)^ AQDS,^b^ ^)^ AR^b^ ^)^	NT	NT	NT	NT
Graphite Oxide (Electrochemical Oxidation)^a)^,^[^ ^211^ ^]^	Catechol,^b^ ^)^ HQ,^b^ ^)^ DCHQ^b^ ^)^	NT	NT	NT	NT
MWCNTs^a)^,^[^ ^212^ ^]^	HQ,^b^ ^)^ Catechol^b^ ^)^	NT	NT	NT	NT
La_2_O^[^ ^208^ ^]^	NT	NT	4‐HO‐ TEMPO^b^ ^)^	MV^b^ ^)^	NT
SrO^[^ ^208^ ^]^	NT	NT	4‐HO‐ TEMPO^b^ ^)^	MV^b^ ^)^	NT
Pyridine^[^ ^213^ ^]^	Catechol,^b^ ^)^ HQ^b^ ^)^	NT	NT	NT	NT
Fe/Co based^a)^,^[^ ^214^ ^]^	Quinone derivatives^b^ ^)^	NT	NT	NT	NT
Pt, Au, Hg*^[^ ^215^ ^]^ * tested in non‐polar solvent	BQ^b^ ^)^	NT	NT	NT	NT

^a)^ Also done for Vanadium Redox Flow Battery;

^b)^ Half cell measurements conducted to show improvement;

Quinones: AQS = Anthraquinone‐2‐sulfonic acid, AQDS = Anthraquinone‐2,7‐disulfonic aid, JN = Juglone, NN = Naphthazarin, AR = Alizarin red, ARS = Alizarin red S, DHAQ = Dihydroxyanthraquinone, DHAQDS = Dihydroxyanthraquinone disulfonic acid, DHAQDMS = 1,4‐dihydroxyanthraquinone‐2,3‐dimethyl sulfonic acid, DBEAQ = 4, 4′‐((9,10‐anthraquinone‐2,6‐diyl)dioxy)dibutyrate, DPPEAQ = (((9,10‐dioxo‐9,10‐dihydroanthracene‐2,6‐diyl) bis(oxy))bis(propane‐3,1‐diyl))bis(phosphonic acid), DHBQ = 2,5‐dihydroxy‐1,4‐benzoquinone, TMHQ = tetramorpholinohydroquinone, HCNQ = Hydroxy‐carboxy naphthoquinone, DHDMBS = 3,6‐dihydroxy‐2,4‐dimethylbenzenesulfonic acid, BQDS = 4,5‐dihydroxybenzene‐1,3‐disulfonic acid, DHBQDS = 1,2‐dihydrobenzoquinone‐ 3,5‐disulfonic acid, FQH2 = 2,3,5,6‐tetrakis((dimethylamino)methyl)hydroquinone, CQH2 = 3,6‐bis(morpholinomethyl)‐ cyclohexa‐3,5‐diene‐1,2‐diol, HQ = Hydroquinone, DCHQ = 2,3‐dicyanohydroquinone, BQ = Benzoquinone; Organometallic Coordination: K_4_[Fe(CN)_6_] = Potassium hexacyanoferrate; Nitroxide Radical Derivatives: TEMPO = 2,2,6,6‐tetramethylpiperidin‐1‐oxyl; Viologens: BTMAP‐Fc = bis((3‐trimethylammonio)propyl)ferrocene, and MV = Methyl viologen.

The performance of organic‐redox couples on treated electrodes are measured in the same way as for VRFBs.^[^ ^41^ ^]^ CVs and EIS are used to compare the peak to peak separation and kinetic reversibility of the redox couple with and without treated electrodes. Several of these treated electrodes are also tested in full‐cell for performance; however, the redox active species used in both compartments are different for different treatments. In Table 1, we summarize all the treatments done for electrodes in AORFBs based on their class and whether the kinetics and performance are improved in the half‐ or full‐cell configurations respectively.

Several treatments shown to improve performance in AORFBs are the same as used in VRFBs. Thermal, Acid/Base, Plasma, Electrochemical Oxidation, Doped with Elements, Carbon‐based electrocatalysts, and Metals (Table 1) are shown to improve charge transfer in organic redox couples similar to vanadium redox couples used in RFBs. The improvement in performance of organic redox couples by similar treatments as VRFBs on carbon electrodes suggests a similarity in the charge transfer mechanism in organic and vanadium redox couples. However, more fundamental work is needed to clearly understand the charge transfer mechanism of organic molecules and capture the effect of specific functional groups and molecular structure on the performance of AORFBs.

Even though the translation of the knowledge from VRFBs to AORFBs is mostly qualitative, this could be useful considering the enormous design space of organic molecules. State‐of‐the‐art AORFBs suffer from degradation of organic molecules rather than sluggish kinetics.^[^ ^18^, ^179^ ^]^ However, considering the vast number of organic molecules that can be synthesized by tailoring the side chains and functional groups, there is a possibility that some of those organic species are stable but have sluggish kinetics. Having an electrode treatment technique to enhance the kinetics independent of the choice of organic molecule could expand the space of molecules usable for AORFBs.

## 7 Conclusions and Future Recommendations

Redox Flow Batteries (RFBs) hold enormous potential for storing energy from intermittent renewables for long durations and releasing energy based on demand. We compare > 70 electrode treatments reported in the literature to improve the performance of VRFBs and make them more competitive for energy storage. The comparison of VRFB performance at laboratory and industrial scale allowed us to identify that performance enhancements from certain treatments at laboratory scale cannot be translated to scaled up VRFBs. We highlight the need of long cycling tests (> 50 cycles) to comment on the stability of electrode treatments. Based on our analysis, we show that the Thermal, Plasma, Electrochemical Oxidation, CO_2_ treatments, and Bi, Ag, and Cu metal electrocatalysts for VRFBs hold the maximum potential for industrial implementation. All these identified promising treatments involve ≤ 6 steps and process units and have capital costs of <40 $ m^−2^ making them highly attractive for industrial scale‐up.

Aqueous‐organic RFBs (AORFBs) have gained a wide interest in recent times due to the potentially low cost of organic redox active molecules compared to vanadium. We observe that the most effective electrode treatments for VRFBs also improve the performance of AORFBs, which suggests a possible similarity in the charge transfer mechanisms of organic and vanadium redox couples. We emphasize the need for more controlled studies to investigate the effect of surface functional groups on charge transfer in organic redox couples in aqueous medium.

As the RFB community moves forward with the continued exploration of old and identification of new chemistries, we make the following recommendations so that the studies conducted in laboratory scale for RFBs can be directly used by researchers around the globe for direct comparison enabling widespread implementation of RFBs:

- Standardization conditions for RFB performance testing: The RFB performance must be reported at a fixed current density with minimum number of cycles. This current density and cycle combination must be considered as universal for testing performance across different redox couples. Additionally, the redox couple on the positive electrode for AORFBs should be kept the same across literature if the negative redox couple is changed (and vice‐versa). We recommend current density of 100 mA cm^−2^ with at least 100 cycles, due to the widespread use of this combination in studies conducted for VRFBs. The standardized testing for solid‐state batteries and solar photovoltaics is now essential to publish in several academic journals.^[^ ^216^, ^217^ ^]^ A recent article was published where established researchers across the globe came forward to recommend experimental protocols in AORFBs.^[^ ^218^ ^]^
- Standardizing conditions for measuring redox couple kinetics: CVs should be conducted at a consistent scan rate and EIS should be conducted at zero overvoltage for fair comparison. Since Δ*E_p_* is dependent on scan rate and *R_ct_* is dependent on voltage, it becomes essential that scan rates for CVs and voltage for EIS are kept consistent between studies. We recommend a scan rate of 100 mV s^−1^ for CVs as 100 mV s^−1^ is the most widely used scan rate in VRFB literature. Multiple scan rates, including 100 mV s^−1^ would be ideal. Additionally, we recommend zero overvoltage for EIS because the *R_ct_* only represents kinetics of charge transfer for redox couples at zero overvoltage.^[^ ^41^ ^]^
- Use of carbon felts with similar properties: CFs differ in performance from vendor to vendor due to the difference in synthesizing process leading to different physical properties. Standardizing and reporting the physical properties of CFs like porosity, conductivity, thickness, and wettability can enable fair comparison of studies across literature.
- Fundamental studies to understand charge transfer: Most of the treatments currently employed in RFBs are based on trial and error approach. Studies focusing on fundamentals of charge transfer can help identify novel treatments that will certainly improve the performance of a redox couple. For example, the potential similarity in the mechanism of organic and vanadium redox couples provides us the opportunity to apply the treasure‐trove of data of treatments in VRFBs to AORFBs.
- Scaling up electrode treatment processes at the industrial scale: Several of the treatments proposed for VRFBs involves numerous tedious steps with requirement of special process units to be implemented in the industrial setting. We recommend researchers to include a section in the publications summarizing their thoughts regarding scaling‐up of the treatment. For example, as we discussed in the review, doping of metals done using hydrothermal method can use the experience of the zeolite industry in scaling up. This will help knowledge from other scientific communities contribute significantly to RFB‘s success.
- Reporting the performance of full‐scale RFBs: Most of the work done currently is under laboratory scale which may not transform to industrial scale RFB. There are very few studies that report the performance of RFBs at hundreds of kW or MW scale. If companies and start‐ups share the performance data for scaled up batteries, that would allow the guidance of future research.

Tremendous progress has been made over the last two decades to improve the various aspects of RFBs; however, a more structured approach with standardization of testing procedures will help in accelerating this process, making RFBs more competitive for energy storage.

## Conflict of Interest

The authors declare no conflict of interest.

## Supporting information

Supporting Information
