# Supplementary material for: Electrode Treatments for Redox Flow Batteries: Translating Our Understanding from Vanadium to Aqueous‐Organic
Source: Adv Sci (Weinh). 2023 Nov 16;11(1):2307209. doi: 10.1002/advs.202307209 (PMC10767411; doi:10.1002/advs.202307209)
Supplement: Supplementary file 1 — Supporting Information [file ADVS-11-2307209-s001.pdf]

ADVANCED SCIENCE
Open Access

Supporting Information

for *Adv. Sci.*, DOI 10.1002/advs.202307209

Electrode Treatments for Redox Flow Batteries: Translating Our Understanding from Vanadium to Aqueous-Organic

Harsh Agarwal, Esha Roy, Nirala Singh, Peter A.A. Klusener, Ryan M. Stephens* and Qin Tracy Zhou*

SUPPORTING INFORMATION

Electrode Treatments for Redox Flow Batteries: Translating our Understanding from Vanadium to Aqueous-organic

Harsh Agarwal,[a,b] Esha Roy,[c] Nirala Singh,[a] Peter A.A. Klusener,[c] Ryan M. Stephens,[b] Qin (Tracy) Zhou[b]

[a]*Department of Chemical Engineering and Catalysis Science and Technology Institute, University of Michigan, Ann Arbor, Michigan 48109-2136, United States*
[b]*Shell International Exploration and Production Inc., 3333 Highway 6 South, Houston, Texas 77082, United States*
[c]*Shell Global Solutions International B.V. Energy Transition Campus Amsterdam, Grasweg 31, 1031 HW Amsterdam, The Netherlands*

S1. Details of Experimental Conditions, Carbon Felt Treatments, and Metal and Metal Oxide Electrocatalysts

Table S1. Conditions under which VRFB with different carbon felts treatments have been tested. Negative half refers to V^{2+}/V^{3+} and positive half refers to VO_2^+/VO^{2+} redox couple. "NA" refers to the information not being reported in the reference, not necessarily that the item is not used. Operating current densities are based on geometric areas of carbon felt electrodes.

ID	Treatment on negative half (Yes/No)	Treatment on positive half (Yes/No)	Carbon felt source	Membrane	Electrode area (cm^2)	[V] (M)	[H_2SO_4] (M)	Charging/ discharging potentials (V)	Cycles	Operating current densities (mA/ cm^2)	Compared to untreated felt? (Yes/ No)	Ref.
Thermal-1	Yes	Yes	Fiber, Material Inc., USA (3 mm thick)	NA	NA	2	2.5-3.5	1.7/1.0	50	25, 40, and 60	Yes	[1]
Thermal-2	Yes	No	Sigracell GFD 4.6 EA, SGL Carbon GmbH, Mettingen, Germany	FAP 450, Fumasep	20	1.6	4	1.65/0.9	60	60, 80, and 100	Yes	[2]
Thermal-3	Yes	Yes	GF-3F, PAN-Nippon Chem	Nafion 117	33	2	2.5	1.7/0.8	5	40	Yes	[3]
Thermal-4	Yes	Yes	GFD 3A, SGL Carbon (3 mm thick)	VX20, Fumatech	25	1.6	2	1.8/0.7	100	80	No	[4]
Thermal-5	Yes	Yes	PAN, Nippon Carbon (3 mm thick)	Nafion 117	9	1.5	3	1.6/0.8	50	50, 75, 100, and 150	Yes	[5]
Thermal-6	Yes	Yes	SGL carbon	Nafion 117	10	1.8	1.6	1.6/1.0	100	50	Yes	[6]
Thermal-7	Yes	Yes	GF-3F PAN graphite felt, Nippon Carbon Inc. (3 mm thick)	Nafion 117	25	1.7	2.5	1.7/0.8	20	32, 40, 80, and 120	Yes	[7]
Thermal-8	Yes	Yes	GF, Gansu Haoshi Carbon Fiber Co. Ltd.(5 mm thick)	Nafion 115	25	1.5	2	1.65/0.8	50	50, 100, 150, 200, and 250	No	[8]
Plasma-1	Yes	Yes	GF-3F, PAN-Nippon Chem	Nafion 117	33	2	2.5	1.7/0.8	5	40	Yes	[3]
Plasma-2	Yes	Yes	GFD 3A, SGL (3 mm thick)	VX20, Fumatech	25	1.6	2	1.8/0.7	100	80	No	[4]
Plasma-3	Yes	Yes	Sigracell (4.6 mm thick)	Nafion 115	25	1	3	1.6/1.0	200	25,50,75, and 100	Yes	[9]
Plasma-4	Yes	Yes	C0S1011 Porous graphite felt, CeTech Taiwan (6 mm thick)	Nafion 117	10.24	1.5	2	1.7/0.7	3	40, and 80	Yes	[10]
Gamma ray	Yes	Yes	GF-3F, PAN-Nippon Chem	Nafion 117	33	2	2.5	1.7/0.8	5	40	Yes	[3]
Acid-H_2SO_4	Yes	Yes	FMI graphite felt (3 mm thick)	NA	NA	2	2.5-3.5	1.7/0.9	20	25, 40, and 60	Yes	[11]
Acid-HNO_3	Yes	Yes	FMI graphite felt (3 mm thick)	NA	NA	2	2.5-3.5	1.7/0.9		25, 40, and 60	Yes	[11]
Echem Oxidation-1	No	Yes	PAN GF, Shanghai Qijie Limited Co. (5 mm thick)	Nafion 117	25	2	3	1.5-1.6/ 0.8	2	40, 50, 60, 70, and 80	Yes	[12]
Echem Oxidation-2	Yes	Yes	PAN CF, Shanghai Energy Carbon Limited	PE-01, Hangzhou Qianqiu Water Treatment Co. China	216	1.6	3	1.8/0.8	20	20,30,40, and 50	No	[13]
Echem Pulse	Yes	Yes	Shenhe Carbon Fiber Materials Ltd. (6 mm thick)	Perfluorinated ion-exchange	9	1.2	3	1.7/0.7	50	30	Yes	[14]

ID	Treatment on negative half (Yes/No)	Treatment on positive half (Yes/No)	Carbon felt source	Membrane	Electrode area (cm^2)	[V] (M)	[H_2SO_4] (M)	Charging/ discharging potentials (V)	Cycles	Operating current densities (mA/ cm^2)	Compared to untreated felt? (Yes/ No)	Ref.
				membrane, Best Industrial & Trade Co. Ltd., China								
N doped-1	No	Yes	PAN graphite felts, Shenhe Carbon Fiber Materials (6 mm thick)	PE-101, Zhejiang Qianqiu Group Co Ltd, China	NA	1.5	3	1.7/0.7	40	20, 30, 40, 50, and 60	Yes	[15]
N doped-2	Yes	Yes	PAN graphite felt, Shenhe Carbon Fiber Material Ltd (5 mm thick)	Perfluorinated ion-exchange membrane, Best Industrial & Trade Co. Ltd., China	9	1.2	3	1.7/0.7	50	20, 30, 40, 50, 60, and 70	Yes, only at 20	[16]
N doped-3	Yes	Yes	PAN graphite felt	Nafion 117	25	1	3	1.9/0.8	30	20	No	[17]
N doped-4	Yes	No	GF-20-3, Nippon Carbon	Nafion 117	9	1.5	3	1.6/0.8	50	50, 75, 100, and 150	Yes	[18]
N, O doped	Yes	No	XF-30A, Toyobo, Japan (4.2 mm thick)	Nafion 115	9	1.6	4	1.6/0.8	20	40, 60, 80, and 100	Yes	[19]
N, S doped	Yes	Yes	PAN based Graphite felt, AvCarb	Nafion 212	15	1	4	1.7/0.8	10	50, 75, 100, 125, 150, 175, and 200	Yes, only at 50 and 75	[20]
B, O doped	Yes	Yes	SGL Carbon	Nafion 117	10	1.8	1.6	1.6/1.0	100	50, 80, 110, 140, and 170	Yes, only at 50	[6]
P doped	Yes	Yes	GF-3F PAN Graphite Felt, Nippon Carbon Inc. (3 mm thick)	Nafion 117	25	1.7	2.5	1.7/0.8	20	32, 40, 80, and 120	Yes	[7]
H_2O_2	Yes	Yes	GF-3F PAN Carbon Felt, Nippon Carbon Co. (3 mm thick)	Nafion 117	25	1.7	2.5	1.7/0.8	20	32, 72, and 146	Yes	[21]
Corona Discharge + H_2O_2	Yes	Yes	GF-3F PAN Carbon Felt, Nippon Carbon Co. (3 mm thick)	Nafion 117	25	1.7	2.5	1.7/0.8	20	32, 72, and 146	Yes	[21]
HF + H_2O_2	No	Yes	PAN graphite felt, Shenhe Carbon Fiber Material Co. China	Nepem-1110, Perfluorinated ion-exchange membrane, Best Industrial, China	9	1.2	3	1.7/0.7	50	50	Yes	[22]

ID	Treatment on negative half (Yes/No)	Treatment on positive half (Yes/No)	Carbon felt source	Membrane	Electrode area (cm^2)	[V] (M)	[H_2SO_4] (M)	Charging/ discharging potentials (V)	Cycles	Operating current densities (mA/ cm^2)	Compared to untreated felt? (Yes/ No)	Ref.
Fenton's Reagent + H_2O_2	No	Yes	PAN Carbon Felt, Shenhe Carbon Felt Inc. (6 mm thick)	Nepem-1110, Perfluorinated ion-exchange membrane, Best Industrial, China	30	1.5	3	1.7/0.7	30	20,40, and 60	Yes, only at 60	[23]
Ozone	Yes	Yes	PAN, Nippon Carbon (3 mm thick)	Nafion 117	9	1.5	3	1.6/0.8	50	50, 75, 100, and 150	Yes	[5]
CO_2	Yes	Yes	CeTech	Nafion 117	25	1.6	2.5	1.6/0.7	30	50,60,70, and 80	Yes	[24]
Defects vis NiO/Ni	Yes	Yes	GF-20-3F PAN graphite felt, Nippon carbon (3 mm thick)	Nafion 117	16	2	4	1.6/0.8	50	50, 75, 100, and 150	Yes, only ay 50, 75, and 100	[25]
Porous felt using K_2FeO_4	Yes	No	NA	Nafion 115	25	1.6	3	1.7/0.7	50	50, 80, 110, 140, 170, and 200	Yes, only at 50, 80, 110, 140, and 170	[26]
Porous felt using Fe	Yes	Yes	GF, Gansu Haoshi Carbon Fiber Co. Ltd. (5mm thick)	Nafion 115	25	1.5	2	1.65/0.8	50	50, 100, 150, 200, 250, and 300	No	[8]
SWCNTs	Yes	Yes	NA	Nafion 212	6.25	1.5	2	1.7/0.8	30	20	Yes	[27]
MWCNTs-1	No	Yes	NA	Nafion 212	6.25	1.5	2	1.7/0.7	NA	20, and 70	No	[28]
MWCNTs-2	Yes	Yes	PAN graphite felt, Shenhe Carbon Fiber Materials Co. Ltd. (4 mm thick)	Nafion 212	28	1.5	2	1.65/0.75	50	50	Yes	[29]
MWCNTs-3	No	Yes	PAN based GF, Shenhe Carbon Fiber Materials Co. Ltd. (3 mm thick)	Nafion 115	9	1.5	4	1.7/0.8	20	80	Yes	[30]
Bamboo like CNTs	Yes	Yes	CeTech	Nafion 117	25	1.5	4	1.7/0.7	70	40, 50, 60, 70, and 80	Yes	[31]
Sulfonated CNTs	Yes	No	NA	Nepem-1110, Proton Exchange Membrane, Best Industrial & Trade Co., China	9	1.6	3	1.7/0.7	50	30, 40, 60, 80, 100, and 120	Yes	[32]
N doped CNTs	Yes	Yes	NA	Nafion 117	5	1	2	1.7/0.8	50	10	Yes	[33]
P doped CNTs	Yes	Yes	XF30A PAN Graphite Felt, Toyobo Japan	Nafion 117	4	1.5	2	1.7/0.8	5	120, 160, and 200	Yes	[34]

ID	Treatment on negative half (Yes/No)	Treatment on positive half (Yes/No)	Carbon felt source	Membrane	Electrode area (cm^2)	[V] (M)	[H_2SO_4] (M)	Charging/ discharging potentials (V)	Cycles	Operating current densities (mA/ cm^2)	Compared to untreated felt? (Yes/ No)	Ref.
N, S doped MWCNTs	No	Yes	PAN Graphite felt, Shenhe Carbon Fiber Materials Co. Ltd (6 mm thick)	Nepem-1110, Best Industrial & Trade Co., China	9	1.6	3	1.7/0.7	40	50	Yes	[35]
Carbon Nanofiber/ CNT composite	Yes	Yes	CF-20-3 PAN, Nippon carbon	Nafion 117	5	2	3	1.6/0.8	30	40, 60, 80, and 100	Yes	[36]
Tris(hydroxymethyl) Aminomethane CNTs	Yes	Yes	GFD4,6, Sigracell PAN Graphite felt (4.6 mm thick)	Nafion 117	4	1.5	3	1.65/0.8	200	80, 120, 160, 200, and 250	Yes	[37]
Graphene Nanoplatelets	Yes	Yes	CF-20 Carbon Felt, Alfa Aesar (6 mm thick)	Nafion 117	2	1.5	4	1.7/0.8	4	40	Yes	[38]
Br doped graphene nanoplatelets	Yes	Yes	CF-20-3 PAN, Nippon Carbon	Nafion 117	5	3	3	1.65/0.8	50	50, 75, 100, and 150	Yes, only at 50	[39]
Carbon dots	Yes	Yes	PAN Graphite Felt, Gansu Haoshi Carbon Fiber Co. Ltd.	Nafion 212	25	1.5	2	1.65/0.8	100	50, 100, 150, 200, 250, 300, and 350	Yes, only at 50, 100, 150, 200, and 250	[40]
N, P doped carbon microspheres	No	Yes	NA	Perfluorinated ion-exchange membrane	9	1.6	3	1.7/0.7	25	50, 60, 70, 80, 90, and 100	Yes	[41]
N doped carbon nanospheres	Yes	Yes	Gansu Haoshi Carbon Fiber Co. Ltd (5 mm thick)	Nafion 115	25	1.5	2	1.65/0.8	300	50, 100, 150, 200, 250, and 300	Yes, only at 50, 100, 150, and 200	[42]
N doped carbon black	Yes	Yes	CF-200-3 PAN graphite felt, Nippon carbon	Nafion 117	5	2	3	1.65/0.8	50	50, 75, 100, and 150	Yes, only ay 50 and 75	[43]
Graphite oxide-based graphene	No	Yes	No graphite felt used	Nafion 212	1	0.5	1	1.5/1.2	30	10, 20, 30, 40, and 50	No	[44]
GO-rGO on Graphene Foam	Yes	Yes	CP, Sinopharm Chemical Reagent Co. Ltd.	Nafion 212	24	1.7	3	1.65/0.75	50	50	Yes	[45]

Table S2. Conditions under which VRFB with different metal and metal oxide electrocatalysts have been tested. Negative half refers to V^{2+}/V^{3+} and positive half refers to VO_2^+/VO^{2+} redox couple. "NA" refers to the information not being reported in the reference, not necessarily that the item is not used. Operating current densities are based on geometric areas of carbon felt electrodes.

ID	Treatment on negative half (Yes/No)	Treatment on positive half (Yes/No)	Carbon felt source	Membrane	Electrode area (cm^2)	[V] (M)	[H_2SO_4] (* HCl) (M)	Charging/ discharging potentials (V)	Cycles	Operating current densities (mA/ cm^2)	Compared to untreated felt? (Yes/ No)	Ref.
Pt/ Carbon black	No	Yes	GF060A01800, CeTech Co Ltd. (5 mm thick)	Nafion 117	25	1	1	1.6/0.8	NA	10	Yes	[46]
Bi-1	Yes	No	Gansu Haoshi Carbon Fiber Co. Ltd (5 mm thick)	Nafion 115	25	1.5	2	1.65/0.8	600	50, 100, 150, 200, 250, 300, 350, and 400	Yes	[47]
Bi-2	Yes	Yes	GFD 4.6 SGL group Germany, (4.6 mm thick)	Nafion 115	48	1.5	3	1.55/1.0	5	80, 100, 120, 140, and 160	Yes, only at 80, 100, 120, and 140	[48]
Bi-3	Yes	No	Dalian Longtian Tech, China (3 mm thick)	Nafion 212	4	1.5	3	1.7/0.8	100	60, 80, 100, 120, 140, 160, 180, and 200	Yes, only at 60, 80, 100, 120, 140, and 160	[49]
Bi-4	Yes	No	Dalian Longtian Tech, China (3 mm thick)	Nafion 212	4	1.5	3	1.7/0.8	100	60, 80, 100, 120, 140, 160, 180, and 200	Yes, only at 60, 80, 100, 120, 140, and 160	[49]
Bi-5	Yes	Yes	Yongdeng Industrial Felt	Nafion 115	48	1.5	3	1.55/1.0	300	80, 100, 120, 140, and 160	Yes	[50]
Bi-6	Yes	No	GFD5EA, SGL carbon company	Nafion 115	10	2	5*	1.6/0.8	50	50, 75, 100, and 150	Yes	[51]
Cu	Yes	Yes	GFA series, SGL Carbon (1.5 mm thick)	Nafion 212	4.7	1	3	1.7/0.9	50	100, 150, 200, and 300	Yes	[52]
Ag	Yes	No	Jingu Carbon Material Co. Ltd.	Nafion 212	12	1.6	3	1.55/1.0	500	80, 120, 160, and 200	Yes, only at 80, and 120	[53]
Sn	Yes	Yes	PAN based (4 mm thick)	Nafion 117	25	1.5	3	1.6/0.7	70	50, 75, 100, and 150	Yes	[54]
Sb	Yes	Yes	PAN based, Shenhe Carbon Fiber Materials	Perfluorinated ion exchange membrane, Best Industrial Co Ltd	30	1.2	3	1.7/0.7	53	60, 80, 100, and 120	Yes	[55]
IrO_2	No	Yes	PAN carbon felt, Shanghai Carbon works (5 mm positive half and 10 mm negative half)	Nafion 117	10	1	2	1.75/0.8	10	20, 30, 40, 50, and 60	No	[56]
ZrO_2	Yes	Yes	NA	Nafion 115	25	1.5	3	1.65/0.8	200	50, 100, 150, 200, and 250	Yes, only at 50, 100, 150, and 200	[57]

ID	Treatment on negative half (Yes/No)	Treatment on positive half (Yes/No)	Carbon felt source	Membrane	Electrode area (cm^2)	[V] (M)	[H_2SO_4] (* HCl) (M)	Charging/ discharging potentials (V)	Cycles	Operating current densities (mA/ cm^2)	Compared to untreated felt? (Yes/ No)	Ref.
SnO_2	Yes	Yes	XF-30A Toyobo	Nafion 117	25	1.5	3	1.6/0.7	50	50, 75, 100, and 150	Yes	[58]
NiO	Yes	Yes	GF-20-3F PAN, Nippon Carbon, (3 mm thick)	Nafion 117	16	2	3	1.7/0.8	300	50, 75, 100, 125, and 150	Yes	[59]
Co_3O_4-1	Yes	Yes	Dalian Longtian Technology Co. Ltd (5 mm thick)	Nafion 117	10	1.5	3	1.75/0.8	100	80, 160, 200, 240, and 300	No	[60]
Co_3O_4-2	Yes	Yes	Dalian Longtian Technology Co. Ltd (5 mm thick)	Nafion 117	10	1.5	3	1.75/0.8	100	80, 160, 200, and 240	No	[60]
Cr_2O_3	Yes	Yes	GF-20-3FE PAN, Nippon carbon (3 mm thick)	GN-114C, cationic, Nanjing General Energy New Material Tech Co. Ltd.	4	1	2.5	1.6/0.8	50	100 and 150	Yes	[61]
CoO	Yes	Yes	GF-20-3FE PAN, Nippon Carbon (3 mm thick)	GN-114C, cationic, Nanjing General Energy New Material Tech Co. Ltd.	4	1	2.5	1.6/0.8	50	75, 100, 125, and 150	Yes	[62]
$NiCoO_2$	Yes	Yes	GF-20-3FE PAN, Nippon Carbon (3 mm thick)	GN-114C, cationic, Nanjing General Energy New Material Tech Co. Ltd.	4	1	2.5	1.6/0.8	50	75, 100, 125, and 150	Yes	[62]
Ta_2O_5	Yes	Yes	PAN based felt	Nafion 117	25	1.6	2.5	1.6/0.7	100	80	Yes	[63]
Nd_2O_3	Yes	Yes	GFA 6EA, SGL Carbon	Nafion 117	10	1.6	2	1.7/0.8	50	100	No	[64]
Mn_3O_4	Yes	Yes	GF-3F PAN, Nippon carbon	NA	NA	2	2.5	1.7/0.8	30	40	Yes	[65]
H: Rutile TiO_2	Yes	No	Mersen SA, Spain (5 mm thick)	Nafion 117	4	1	3	1.8/0.8	10	12.5, 25, 37.5, 50, 75, 100, 125, and 150	Yes, only at 25, and 50	[66]
$KMnO_4$	Yes	Yes	GFA 6EA, Sigracell SGL Carbon (6 mm thick)	Nafion 212	4	1	3	1.65/0.8	550	100, 150, 200, 250, 300, and 350	Yes, only at 100, 150, 200, 250, and 300	[67]
CeO_2	Yes	Yes	PAN Graphite Felt, Gansu Haoshi Carbon Fiber (5 mm thick)	Nafion 115	25	2	2	1.65/0.8	100	50, 100, 150, 200, and 250	Yes, only at 50, 100, 150, and 200	[68]

ID	Treatment on negative half (Yes/No)	Treatment on positive half (Yes/No)	Carbon felt source	Membrane	Electrode area (cm^2)	[V] (M)	[H_2SO_4] (* HCl) (M)	Charging/ discharging potentials (V)	Cycles	Operating current densities (mA/ cm^2)	Compared to untreated felt? (Yes/ No)	Ref.
Prussian Blue	Yes	Yes	Jingu Carbon Material Co. Ltd.	Nafion 117	13.5	1.5	3	1.75/0.7	100	50, 80, 100, 150, 200, 250, 300, 320, 340, 360, and 380	Yes, all except at 380	[69]
CeO_2: Espun Nanofiber	Yes	Yes	Liao Yang JinGu Carbon Fibers Sci-Tech Co. Ltd.	Nafion 212	28	1.7	3	1.65/0.75	50	60, 70, 80, and 100	Yes	[70]
TiO_2: Espun Nanofiber	Yes	Yes	PAN felt	Ion exchange membrane	9	1.6	3	1.7/0.7	25	50, 60, 70, 80, and 90	Yes	[71]
Nb_2O_5	Yes	Yes	GFD5EA, SGL Carbon, Germany	Nafion 115	5	2	5*	1.6/0.8	50	50, 75, 100, and 150	Yes	[72]
Nb_2O_5-W	Yes	Yes	GFD5EA, SGL Carbon, Germany	Nafion 115	5	2	5*	1.6/0.8	50	50, 75, 100, and 150	Yes	[72]

Table S3. Conditions used for various carbon felt treatments in literature and corresponding unique IDs that are used to identify them in this work. Number of steps and process units (PU) needed to implement the process industrially are identified to evaluate complexity of treatment.

**Thermal**				
**ID**	**Atmosphere**	**Treatment Details**	**No. of Steps/PU/ Total**	**Ref.**
Thermal-1	Air	*Steps:* Treated in air at 400 °C for 30 h (optimized time and temperature)<br>*PU:* Furnace	1/1/2	[1]
Thermal-2	Air	*Steps:* 1) Washed with ethanol and deionized water, 2) Treated in air in a muffle furnace at 750 °C for 5 mins, using a ramp rate of 5 °C min^{-1} (optimized time and temperature)<br>*PU:* Washing vessel, Furnace	2/2/4	[2]
Thermal-3	Air	*Steps:* 1) Treated at 500 °C for 5 h in air at 10 °C min^{-1} (optimized temperature)<br>*PU:* Furnace	1/1/2	[3]
Thermal-4	Air	*Steps:* 1) Treatment at 500 °C in air in a muffle furnace at 5 °C min^{-1} (time not mentioned)<br>*PU:* Furnace	1/1/2	[4]
Thermal-5	Air	*Steps:* 1) Felt placed in a cylindrical quartz column (50 mm ID, 55 mm outer diameter), and air passed at 500 °C for 5 h<br>*PU:* Furnace	1/1/2	[5]
Thermal-6	Air	*Steps:* 1) Treated at 400 °C for 5 h in air, ramp rate 15 K min^{-1}<br>*PU:* Furnace	1/1/2	[6]
Thermal-7	Ar	*Steps:* 1) Treated at 800 °C for 2 h in Ar atmosphere<br>*PU:* Furnace	1/1/2	[7]
Thermal-8	Air	*Steps:* 1) Treated in air at 420 °C for 10 h<br>*PU:* Furnace	1/1/2	[8]
**Plasma**				
**ID**	**Atmosphere**	**Treatment Details**	**No. of Steps/PU/ Total**	**Ref.**

Plasma-1	O_2	*Steps:* 1) O_2 Plasma treatment for 2 mins (optimized time) in inductively coupled radio frequency plasma fabrication reactor (applied power 100W), with flow rate of O_2 as 5 sccm<br>*PU:* O_2 plasma reactor	1/1/2	[3]
Plasma-2	O_2	*Steps:* 1) O_2 plasma treatment for 4 mins at 20% maximum power (rated as 200 W) in a radio frequency 12.56 MHz plasma setup. Felts are loaded, followed by evacuating the chamber below 0.2 mbar pressure and subsequently filling with O_2 till 0.8 mbar pressure<br>*PU:* O_2 plasma reactor	1/1/2	[4]
Plasma-3	O_2+ N_2	*Steps:* 1) O_2:N_2 plasma (1:1 v/v) composition (inductively coupled radio frequency, 13.56 MHz) at a power of 100 MW and process pressure of 16 Pa (optimized) for 10 mins<br>*PU:* O_2 plasma reactor	1/1/2	[9]
Plasma-4	N_2	*Steps:* 1) The applied voltage and on/off duty cycle for atmospheric plasma jet treatment are 275 V and 7/33 µs respectively and the N_2 flow rate is 60 standard liters per min. The graphite felt is treated for 1 minute.<br>*PU:* O_2 plasma reactor	1/1/2	[10]
**Gamma Ray Irradiation**				
**ID**	**Atmosphere**	**Treatment Details**	**No. of Steps/PU/ Total**	**Ref.**
Gamma ray		*Steps:* 1) 150 kGy optimized dose for carbon felt at a rate of 10 kGy h^{-1} in a gamma irradiator with ^{60}Co source<br>*PU:* Gamma irradiator	1/1/2	[3]
**Acid**				
**ID**	**Chemical**	**Treatment Details**	**No. of Steps/PU/ Total**	**Ref.**
Acid- H_2SO_4	H_2SO_4	*Steps:* 1) Reflux in 98% H_2SO_4 for 5 h (optimized time and acid concentration based on cell resistance)<br>*PU:* Acid-resistant container	1/1/2	[11]
Acid-HNO_3	HNO_3	*Steps:* 1) Reflux in 70% HNO_3 for 5 h (optimized time and acid concentration based on cell resistance)<br>*PU:* Acid-resistant container	1/1/2	[11]
**Electrochemical Oxidation**				
**ID**	**Chemical**	**Treatment Details**	**No. of Steps/PU/ Total**	**Ref.**
Echem Oxidation-1	H_2SO_4	*Steps:* 1) Graphite felt was used as anode, and graphite plate was used as cathode. The felt was electro-oxidized at 100 mA cm^{-2} in 1 M H_2SO_4 passing charge of 560 mAh g^{-1}, 2) Graphite felt was then washed with deionized water, and 3) Dried at 70 °C for 48 h<br>*PU:* Potentiostat, Washing vessel, Oven	3/3/6	[12]
Echem Oxidation-2	H_2SO_4	*Steps:* 1) Graphite Felt was used as anode, and Ti plate as cathode, carried out in 1 M H_2SO_4. Potential between 5-15 V is applied to galvanically oxidize passing 3000 C g^{-1} mass of felt, 2) Felt was washed with deionized water, and 3) Dried in a vacuum oven at 120 °C for 5 h<br>*PU:* Potentiostat, Washing vessel, Oven	3/3/6	[13]
Echem Pulse	NaOH	*Steps:* 1) Graphite Felt was used as working, Saturated Calomel as reference, and Pt as counter electrode. Square wave potential pulse between −1.3 V to 0.6 V at a frequency of 5 Hz was applied for 1600 seconds (optimized) in 2 M NaOH<br>*PU:* Potentiostat, Washing vessel	1/2/3	[14]
**Doped**				
**ID**	**Atmosphere / Chemical / Method**	**Treatment Details**	**No. of Steps/PU/ Total**	**Ref.**
N doped-1	Ammoniated	*Steps:* 1) Graphite felt thermally treated in a sealed 50 mL Teflon-lined stainless steel autoclave containing 15 mL 25% ammonia solution at 180 °C for 15 h (optimized time), 2) Washed with deionized water until pH is neutral, 3) Dried in vacuum oven at 110 °C for 5 h<br>*PU:* Autoclave, Furnace, Washing vessel, pH meter, Oven	3/5/8	[15]
N doped-2	NH_3 atmosphere	*Steps:* 1) Tubular furnace purged with NH_3 atmosphere for 30 mins at 1 L min^{-1} to get rid of air and felts are placed and heated to 900 °C for 150 mins (at ramp rate of 5 °C min^{-1}) in NH_3 atmosphere (rate of 0.3 L min^{-1})	1/1/2	[16]

		PU: Furnace		
N doped-3	NH_3 + O_2 atmosphere	*Steps:* 1) Felts treated thermally under NH_3/O_2 (1:1) atmosphere at 500 °C for 24 h (optimized time and temperature)<br>*PU:* Furnace	1/1/2	[17]
N doped-4	Chemical Oxidative Polymerization using Ammonium persulfate	*Steps:* 1) Pyrrole monomer and ethanol were mixed thoroughly and graphite felt was stirred in this solution for 1 h, 2) 124 mg of ammonium persulfate (APS) was slowly added and stirred for 12 h at 40 °C, 3) 300 mg of cobalt nitrate was added to water and impregnated on carbon felt and dried at 80 °C for 4 h, 4) Resultant felt was pyrolyzed under Ar at 900 °C for 1 h, 5) Treated with 4 M sulfuric acid at 80 °C for 3 h to remove any Cobalt in felt, and 6) Washed thoroughly with water before use<br>*PU:* Temperature-controlled mixer, Tool for impregnation, Oven, Furnace, Acid-resistant vessel, Washer	6/6/12	[18]
N, O doped	RSF	*Steps:*<br>Preparation of RSF solution: 1) Cocoons from silkworms obtained from Uljin farm were boiled for 25 mins in 0.02 M Na_2CO_3, 2) Washed with deionized water, 3) Dried at room temperature for 3 days, 4) Dissolved in an aqueous solution of 9.3 M LiBr at 60 °C for 6 h, 5) Dialyzed in water using dialysis cassette for 2 days to get 8 wt% RSF solution<br>Coating on carbon felt: 1) Carbon felt was dip-coated in an aqueous 0.5 wt% solution of RSF (or glucose O source, or melamine N source), 2) Dried in a vacuum oven for 30 °C for 3 h, 3) Heated in tube furnace for 2 h at 400 °C (at 5 °C min^{-1}) in Ar (200 mL min^{-1})<br>*PU:* Boiler, Washer, Oven (2), Mixer, Dialysis cassette, Dip-coater, Furnace	8/8/16	[19]
N, S doped	Ammonium persulfate	*Steps:* 1) 0.25 M Ammonium persulfate is dissolved in 100 mL deionized water and 1 g of carbon felt was immersed in it, 2) The solution with felt was then placed in a 200 mL Teflon lined hydrothermal reactor heated in an air furnace at 180 °C for 12 h, 3) Rinsed with deionized water to remove residual salt from the surface<br>*PU:* Mixer, Autoclave, Furnace, Washer	3/4/7	[20]
B, O doped	Boric acid	*Steps:* 1) Thermally treated at 400 °C for 5 h in air (ramp rate 15 °C min^{-1}), 2) Oxygen-treated GF was immersed in ethanol solution (100 mL) of boric acid (0.1 and 1 M) at 298 K for 1 h with stirring, 3) Dried in oven at 80 °C for 4 h, 4) Thermally treated in N_2 for 1.5 h at 700 °C, 5) Washed with ethanol at 50 °C under sonication for 1 h and dried at ambient temperature<br>*PU:* Furnace (2), Mixer, Oven, Washer with sonication	5/5/10	[6]
P doped	Ammonium hexafluorophosphate	*Steps:* 1) Immersed in 2 M NH_4PF_6 solution in deionized water, 2) Dried in oven at 800 °C for 2 h under Ar atmosphere, 3) Washed with distilled water, and 4) Dried at room temperature for 12 h<br>*PU:* Immersion container, Oven, Washer, Drying container	4/4/8	[7]
**H_2O_2 based treatments**				
**ID**		**Treatment Details**	**No. of Steps/PU/ Total**	**Ref.**
H_2O_2		*Steps:* 1) Sonication of the felt in H_2O_2 (30%) for 1 h at 100 W, 2) Dried under vacuum at 80 °C for 12 h<br>*PU:* Sonicator resistant to H_2O_2, Oven	2/2/4	[21]
Corona Discharge + H_2O_2		*Steps:* 1) Carbon felt pre-treated using a HK-2000 corona discharge instrument with one 15 mm dielectric barrier between the electrode and carbon felt, 2) Each side exposed twice for 15 seconds at ambient temperature and under a discharge current of 4 A, 3) Sonication of the prepared felt in H_2O_2 (30%) for 1 h at 100W, 4) Dried under vacuum at 80 °C for 12 h<br>*PU:* Corona discharge instrument, Sonicator resistant to H_2O_2, Oven	4/3/7	[21]
HF + H_2O_2		*Steps:* 1) Soaked in HF (12 wt%) for 36 h, 2) Washed with deionized water until neutralized, 3) Dried under vacuum at 80 °C for 12 h, 4) Kept in a sealed Teflon-lined stainless steel autoclave containing H_2O_2 with the concentration of 5 wt% at 180 °C for 10 h, 5) Rinsed with DI water, 6) Dried under vacuum at 80 °C for 12 h<br>*PU:* Vessel for HF, Washer (2), pH meter, Oven (2), Autoclave, Furnace	6/8/14	[22]
Fenton's Reagent + H_2O_2		*Steps:* 1) Soaked in ethanol with ultrasonication for 0.5 h, 2) Rinsed with deionized water, 3) Dried in vacuum oven at 50 °C for 10 h, 4) Dipped in 20 mL of 0.02 M ferrous solutions (pH 3) for 1 h at room temperature, 5) 15 mL H_2O_2 (30%) was added to the beaker with felt and diluted to 400 mL with water and the felt was treated until no gas evolution was observed (0.5-2 h), 6) Treated felts immersed in 0.1 M H_2SO_4 under ultrasonication for 0.5 h to remove $Fe(OH)_3$, 7) Washed with water until neutral, and 8) Dried in oven at 50 °C for 10 h<br>*PU:* Sonicator (2), Washer (2), Oven (2), Mixer (2), pH meter	8/9/17	[23]
**Gas**				
**ID**		**Treatment Details**	**No. of Steps/PU/ Total**	**Ref.**

Ozone		*Steps:* 1) Felt placed in a cylindrical quartz column (55 mm outer diameter, 50 mm inner diameter) of an electric furnace and was heat treated. Exposed to ozone at 1 L min^{-1} (during heat treatment) generated from gaseous oxygen via a commercial ozone generator (2OG-030, Ozone Engineering), 2) Column purged with Ar to remove ozone molecules after treatment<br>*PU:* Ozone generator, Furnace	2/2/4	[5]
CO_2		*Steps:* Carbon felt treated in a tube furnace at 1000 °C for 30 mins at 20 °C min^{-1} and flow rate of 50 sccm<br>*PU:* Furnace	1/1/2	[24]
		**Porous Felts by Chemical Reaction**		
**ID**	**Chemical**	**Treatment Details**	**No. of Steps/PU/ Total**	**Ref.**
Defects via NiO/Ni	NiO/Ni	*Steps:* 1) Cleaned with acetone for 10 mins, 2) Precursor solution was prepared by dissolving Nickel nitrate hexahydrate ($Ni(NO_3)_2$. $6H_2O$) in acetone followed by ultrasonication for 5 mins, 3) Felt immersed in precursor solution (5 wt%) for 2 h, 4) Dried at 60 °C for 1.5 h in a vacuum oven to obtain a uniform layer of precursor on carbon felt, 5) Thermal treatment at 500 °C in Ar to form NiO nanoparticles, 6) Temperature increased to 600 °C for 30 mins to etch the surface of felt by NiO reduction in Ar gas, 7) Oxidation at 500 °C for 30 mins in air atmosphere to recover NiO etching source (these oxidation at 500 and 600 °C repeated multiple times to control pore size), 8) Felt dipped in 2 M HNO_3 followed by 3 M HCl to remove the metals from the surface, and 9) Thermally treated at 500 °C for 3 h in air<br>*PU:* Vessel for cleaning, Mixer, Ultrasonicator, Oven (min 2), Furnace (min 3), Acid-resistant containers (2)	9/10/19	[25]
Porous felt using K_2FeO_4	K_2FeO_4	*Steps:* 1) Scapium scaphigerum was soaked in deionized water for 3 h to make it fully expand, 2) Core and meridian of scapium scaphigerum was removed, 3) Pup was moved to a Teflon lined autoclave which was sealed and heated at 180 °C for 15 h, 4) Immersed in ethanol to remove organic matter, 5) Dried in oven at 80 °C for 8 h and 6) ground uniformly, 7) K_2FeO_4 and this grounded mixture were mixed at as mass ratio of 1:1, 8) Mixture was carbonized at 800 °C for 2 h in Ar atmosphere, 9) Sample washed with diluted HCl to remove K, Fe, and residual inorganic impurities, 10) Washed with deionized water, 11) Dried at 80 °C, 12) Mixture of 10 mg of prepared catalyst and 30 mL of DMG were mixed to create a suspension, 13) Felt was immersed in this suspension and dried at 80 °C for 4 h<br>*PU:* Soaking container, Tools for core and meridian removal, Autoclave, Furnace (2), Mixer (3), Oven (2), Washer (3)	13/13/26	[26]
Porous felt using Fe	Fe	*Steps:* 1) Thermally treated in air at 420 °C for 10 h, 2) FeOOH nanorods grown on thermally treated felt by hydrothermal method, 3) Annealed in N_2 gas at 900 °C at 5 °C min^{-1} to form Fe_3O_4 from FeOOH on thermally treated felt, 4) Fe_3O_4 nanoparticles were then dissolved by treating with concentrated HCl, and 5) Thermally activated in air at 420 °C for 10 h<br>*PU:* Furnace (4), Autoclave, Mixer	5/6/11	[8]
		**Carbon catalysts**		
**ID**	**Chemical**	**Treatment Details**	**No. of Steps/PU/ Total**	**Ref.**
SWCNTs		*Steps:* 1) Pristine SWCNTs was treated by refluxing in H_2SO_4 + HNO_3 solution (3:1) for 6 h at room temperature, 2) Felts are washed, 3) Dried at 60 °C before use, 4) SWCNT (2% relative to amount of carbon felt) was ultrasonically dispersed in 6mL DMF in which the carbon felt was immersed<br>*PU:* Mixer (2), Washer, Oven, Ultrasonicator	4/5/9	[27]
MWCNTs-1		*Steps:* 1) Felt was ultrasonicated for 20 mins in distilled water, 2) Dried for 4 h at 100 °C in oven, 3) Carboxyl MWCNTs was ultrasonically dispersed in dimethyl formate and then the purified felt was immersed in this solution, 4) Dried in the oven at 100 °C for 24 h<br>*PU:* Ultrasonicator (2), Oven (2), Mixer	4/5/9	[28]
MWCNTs-2		*Steps:* 1) Pristine felts were washed with distilled water by ultrasonication for 30 mins, 2) Dried at 100 °C for 2 h, 3) Carboxyl MWCNTs (1 mg mL^{-1} of Nafion) were added to 0.02 wt% Nafion binder under agitation and the felt was immersed in prepared solution under ultrasonicated for 10 mins, 4) Drying at 100 °C for 6 h<br>*PU:* Ultrasonicator (2), Oven (2), Mixer	4/5/9	[29]
MWCNTs-3		*Steps:* 1) Felts cleaned with 5% H_2O_2 solution at 40 °C for 30 mins, 2) Washed with deionized water, 3) Dried at 90 °C for 6 h, 4) 10g L^{-1} MWCNTs, 20g L^{-1} sucrose, and 3g L^{-1} of sodium dodecyl benzene sulfonate was prepared by ultrasonication, 5) Felt was dipped in this solution for 20 mins, 6) Dried in an oven at 90 °C for 6 h, 7) Heated at 900 °C for 20 mins in air with 1 L min^{-1} N_2 followed by cooling to room temperature<br>*PU:* Vessel resistant to H_2O_2, Washer, Oven (2), Ultrasonicator, Mixer, Furnace	7/7/14	[30]
Bamboo like CNTs		*Steps:*	8/8/16	[31]

		Treatment of graphite felt: 1) Immersed in a mixed solution of 60 mL sulfuric acid and 20 mL nitric acid for 10 mins, 2) Transferred to a Teflon-lined autoclave reactor and heated at 140 °C for 50 mins by hydrothermal method, 3) Dried at room temperature, 4) Felt introduced to a furnace and treated in O_2 at 450 °C.<br>Growing of Bamboo like CNTs by CVD reactor by a catalyst assisted method: 1) The treated felt was immersed in a sol-gel solution consisting of 2 g cobalt nitrate, 50 mL of isopropanol, and 2 mL of highly pure titanium tetra-n-butoxide, 2) Dried at 40 °C in vacuum oven, 3) Felt introduced to CVD reactor fed with H_2 at 30 sccm and then temperature was increased to 700 °C at 10 °C min^{-1}, 4) $H_2/C_2H_2/NH_3$ at flow rate of 30/40/80 sccm fed into CVD reactor for 1 h at 700 °C, causing the growth of bamboo like CNTs<br>*PU:* Mixer (2), Autoclave, Furnace (2), Oven (2), CVD reactor		
Sulfonated CNTs	Chlorosulfonic acid	*Steps:* 1) MWCNTs (Shenzhen Nanotech) were added to chlorosulfonic acid and uniformly mixed, 2) Mixture was transferred to three Teflon autoclaves and then hydrothermally treated for 10 h at 200 °C, 3) Washed with deionized water until neutralized, 4) Dried at 80 °C for 8 h, 5) 3 mg of this catalyst was mixed with 10 mL of DMF to form an ink which is sonicated for 3 h, 6) Felt was then immersed in the ink and dried at 100 °C for 4 h<br>*PU:* Mixer, Autoclaves, Furnace, Washer, pH meter, Oven (2), Sonicator	6/8/14	[32]
N doped CNTs	Ferrocene	*Steps:* 1) N-CNT/GF was synthesized using a single injection CVD system, using ferrocene (2.5 wt%) as a growth catalyst dissolved in ethylenediamine, 2) Synthesis carried out at 800 °C under inert gas flow and steady source solution injection in a horizontal quartz tube reactor (GF placed at center of tube), 3) Reactor chamber cooled to 400 °C and air flow for 1 h to burn off amorphous carbon species<br>*PU:* Mixer, CVD Reactor and associated tools, Furnace (2)	3/4/7	[33]
P doped CNTs	Phosphorylethanolamine	*Steps:* 1) 20 mg of carboxylic MWCNTs were mixed with 10 mL of phosphorylethanolamine solution which was prepared by mixing 500 mg pf phosphorylethanolamine with 10 mL deionized water and stirred for 24 h at room temperature, 2) Mixture centrifuged to remove unreacted solution, 3) Mixture dried at 60 °C for 12 h, 4) 20 mg of catalyst powder mixed with 7 mL of IPA followed by ultrasonication for 30 seconds, 5) Graphite felt submerged in the solution and shaken for 10 mins, 7) Each felt was mixed with 19.9 mL of IPA and 0.1 mL of Nafion and shook for 5 mins, 8) Catalyst coated carbon felt, and 9) Felt was then dried at 60 °C for 12 h<br>*PU:* Mixer (3), Centrifuge, Oven (2), Ultrasonicator, Coater	9/8/17	[34]
N, S doped MWCNTs	Thiourea	*Steps:* 1) MWCNTs and thiourea (4:1 molar ratio) were added to 30 mL of ethanol and stirred for 30 mins, 2) Heated under agitation at 50 °C to evaporate ethanol, 3) Sintered at 800 °C for 2 h under Ar atmosphere, 4) Treated graphite felts were washed with water, 5) Felts dried at 100 °C for 10 h, 6) Felts immersed in MWCNTs dispersed in dimethylformide with concentration of 0.4 mg mL^{-1}, and 7) Dried at 100 °C for 24 h<br>*PU:* Mixer, Heater, Furnace, Washer, Oven (2), Sonicator	7/7/14	[35]
Carbon Nanofiber/ CNT composite		*Steps:* 1) Felts washed by ultrasonication in acetone for 20 mins, 2) Dried at 100 °C for 4 h, 3) Felts immersed in nickel nitrate solution (1wt% $Ni(NO_3)_2$) dissolved in acetone (60 mL) and dried for 3 h at 100 °C, 4) Calcined in an inert atmosphere, 5) Reduced in a mixture of 10% H_2/rest Ar gas at 600 °C for 3 h and cooled to room temperature, 6) Heated in inert gas at 700 °C at 0.5 L min^{-1} flow rate, 7) Acetylene flowed (9.94% C_2H_2/balance Ar) for 10 mins and then cooled to room temperature, 8) Resultant CNF/CNT was refluxed in concentrated HCl for 2 h to remove metal impurities, 9) Washed with deionize water, and 10) Dried at 100 °C in air for 12 h<br>*PU:* Ultrasonicator, Oven (3), Mixer (2), Furnace (2), Washer, Mixer	10/10/20	[36]
Tris(hydroxymethyl) Aminomethane CNTs		*Steps:* 1) 0.61 g of tris (hydroxymethyl)aminomethane was mixed with 10 mL of deionized water at 60 °C, and 40 mg of carboxylic MWCNTs were added to this solution, 2) Stirred at 60 °C for 15 mins, 3) Centrifuged to remove the unreacted species (conducted 5 times), 4) Dried in an oven for 60 °C for 12 h, 5) 20 mg of catalyst was mixed in 7 mL of isopropyl alcohol and sonicated for 30 seconds for proper dispersion, 6) Graphite felt was dipped and stirred for 10 mins in the solution, 7) Graphite felt with ink was soaked into the mixed solution of 19.9 mL of IPA and 0.1 mL of Nafion 117 solution and stirred for 5 mins, 8) Catalyst coated graphite felt was dried at 60 °C for 12 h<br>*PU:* Mixer (3), Centrifuge, Oven (2), Sonicator, Catalyst coater	8/8/16	[37]
Graphene Nanoplatelets		*Steps:* 1) A 5% by weight suspension of hydrolyzed carbon nanoparticles was prepared using deionized water and alcohol-free media was employed to prepare the graphene nanoplatelets suspension, 2) The pH of the colloidal suspension was varied with a 0.5 M sulfuric acid solution to give the nanoplatelets sufficient negative surface charge (zeta potential∼−38 mV at a pH of 7), 3) The suspension was then sonicated for 30 min prior to multilayer assembly, 4) An automated dipping tool (Beckmann Biomek 2000) was used in the preparation of the larger number of layered assemblies. The polymer solution and the GNP suspension were continuously stirred during this operation and dipped felt was rinsed repeatedly. This sequence of steps yielded a single bilayer of nanoplatelets-polyelectrolyte deposition.<br>*PU:* Mixer, pH meter, Sonicator, Dipping tool, Washer	4/5/9	[38]
Br doped graphene nanoplatelets		*Steps:* 1) Pristine graphite (5 g) was placed in diluted halogen (Br_2) in a stainless steel container with stainless steel balls (500 g, 5 mm diameter), 2) The steel container was sealed and degassed after reducing pressure (0.05 mm Hg) to remove air and then fixed in a plenary ball-mill machine and agitated at 500 rpm for 48 h, 3) Prepared samples were extracted with acetone to remove unreactive materials, 4)	8/7/15	[39]

		Washed with 1 M HCl solution to remove metallic impurities, 5) Dark black powders of Br-Graphene nanoplatelets were obtained by freeze drying them at −120 °C for 48 h, 6) Catalysts ink was prepared by dissolving 20 mg of catalyst particles in a mixture of 100 µL of 5wt% Nafion and 900 µL of ethanol by ultrasonically blending for 20 mins, 7) 5 mg cm^{-2} of ink was coated onto a carbon felt, and 8) Carbon felt was then dried at 60 °C for 12 h<br>*PU:* Ball mill, Extractor, Washer, Freeze Dryer, Sonicator, Ink coater, Oven		
Carbon dots		*Steps:* Carbon dots/Graphite Felt were synthesized by solvothermal process: 1) 0.6 g of p-phenyldiamine was dispersed in 90 mL ethanol under sonication, 2) Felt was immersed in solution and the solution is then transferred to a 200 mL Teflon lined autoclaves, and the reaction was conducted at 180 °C for 9 h, followed by cooling down to room temperature, 3) Felt was taken out and washed with water, and 4) Dried at 60 °C for 12 h<br>*PU:* Sonicator, Mixer, Autoclave, Furnace, Washer, Oven	4/6/10	[40]
N, P doped carbon microspheres		*Steps:*<br>Synthesis of Carbon microspheres: 1) 0.5 mol L^{-1} glucose solution was filled in a Teflon tank where hydrothermal reaction occurred, 2) This system was placed in an oven at 180 °C for 24 h, 3) Carbon microspheres obtained by this hydrothermal treatment was washed with anhydrous alcohol, and 4) Dried at 80 °C<br>Doping of Carbon microspheres: 1) Carbonized at 800 °C for 2 h in Ar, 2) Mixed with NH_4Cl as N source and $(NH_4)_2HPO_4$ as N and P source by grinding, 3) Heated at 800 °C for 2 h in Ar to obtain N, P doped carbon microspheres<br>Deposition on Carbon Felt: 1) 10 mg of prepared microspheres was dispersed in 5 mL of N,N-dimethyl formamide by ultrasonication, and then 2) Coated on felt<br>*PU:* Mixer (2), Autoclave, Furnace (2), Oven (2), Washer, Ultrasonicator, Ink coater	9/10/19	[41]
N doped carbon nanospheres		*Steps:* 1) 200 mg of dopamine was dissolved in 100 mL of distilled water with stirring for 30 mins to form a solution at room temperature, 2) Felt was immersed in solution and hold in vacuum chamber for 3 h (for allowing it to penetrate), 3) 75 µL of tris buffer was added to initiate self-polymerization of dopamine, 4) Reaction was carried out for 8 h (optimized) at room temperature with continuous stirring, 5) Felt was washed with deionized water, 6) Dried with 50 °C for 12 h, 7) Carbonized in a tube furnace for 2 h at 900 °C (optimized).<br>*PU:* Mixer (2), Vacuum chamber, Reaction vessel, Washer, Oven, Furnace	7/7/14	[42]
N doped carbon black		*Steps:* 1) 0.1 g of zein powder was stirred in a solvent containing 3 mL ethanol and 3 mL deionized water for 10 mins to obtain a yellow solution, 2) This solution was blended with 0.3 g of carbon black particles (optimum ratio 1:3) to form a coating of zein particles on carbon black, 3) Evaporated at 60 °C to allow particles self-assemble on carbon black, 4) Powder was placed on a furnace for 3 h in Ar at 800 °C (optimized), 5) 20 mg of N doped carbon black particles were dissolved in 100 µL of 5wt% Nafion and 900 µL of ethanol followed by sonication for 20 mins, 6) 5 mg cm^{-2} ink was coated on carbon felt, and 7) Felt was dried at 60 °C for 12 h.<br>*PU:* Mixer (2), Blender, Oven (2), Furnace, Sonicator, Ink coated	7/8/15	[43]
Graphite oxide-based graphene		*Steps:* Prepared using modified Hummer's method requiring $NaNO_3$ and $KMnO_4$. 1) Concentrated H_2SO_4 (360 mL) was added to a mixture of synthetic graphite (7.5 g) and $NaNO_3$ (7.5 g), and the resulting mixture was cooled down using ice bath, 2) $KMnO_4$ (45 g) was added slowly in small doses to keep temperature below 20 °C, 3) Mixture was heated to 35 °C and stirred for 3 h, 4) 3% H_2O_2 (1.5 L) slowly added raising the temperature to 98 °C, 5) Stirred for 30 mins, 6) Centrifuged (3700 rpm for 30 min), 7) Decantation of mixture, 8) Washed with water and 9) Centrifuged again until pH was neutral, 10) Thermally treated at 1000 °C for 1 h under N_2 to obtain thermally prepared graphene oxide<br>*PU:* Mixer (3), Ice bath, Centrifuge (2), Decanter, Washer, pH meter, Furnace	10/10/20	[44]
GO-rGO on Graphene Foam		*Steps:* 1) Graphene was first grown on Ni foam by CVD to obtain NiF/graphene foam (GF), 2) Graphene Oxide (GO) was prepared by a modified chemical exfoliation method with natural graphite as starting material, 3) The NiF/GF was dispersed in acid GO aqueous dispersion (6 mg mL^{-1}, pH =3) for 24 h at 60 °C, 4) The obtained NiF/GF was filled with wet GO gels, 5) Reduced on a Zn foil surface for 3 mins, 6) Sample was then frozen in liquid nitrogen and 7) Frozen sample was freeze-dried for 24 h to obtain a gradient of oxygen functional groups, 8) Immersed in 2 M HCl for 24 h to remove NiF, 9) Repeated washing with purified water, and 10) Wet GO-RGO/GF material was further subjected to freeze drying for 24 h<br>*PU:* CVD Reactor, Sonicator, Potentiostat, Liquid nitrogen container, Freeze dryer (2), Mixer, Washer (2)	10/9/19	[45]

Table S4. Conditions used for preparation and testing of metal and metal-oxide electrocatalysts for VRFBs from literature and corresponding unique IDs that are used to identify them in this work. Number of steps and process units (PU) needed to implement the process industrially are identified to evaluate complexity of treatment.

Metals				
**ID**	**Deposition Method**	**Treatment Details**	**No. of Steps/PU/ Total**	**Ref.**
Pt/ Carbon black		_Steps:_ 1) 1 g of carbon black and 50 mL of deionized water was mixed under ultrasonication for 2 h, 2) 0.495 g $H_2PtCl_6.6H_2O$ (15 wt% Pt/C) was added to mixture, 3) 1 M NaOH was added until the pH reached 11, 4) 10 mL of reducing agent CH_3OH was added under constantly stirring under thermal reflux at 80 °C for 4 h, 5) The mixture was then filtered, washed with deionized water, 6) Dried at 80 °C under vacuum oven, 7) Ink prepared using this Pt/C, and 5 wt% Nafion binder and alcohol was sprayed on carbon felt. and 8) Dried at 65 °C for 6 h in vacuum oven<br>_PU:_ Sonicator, Mixer (2), pH meter, Filter, Washer, Oven (2), Spray Coater	8/9/17	[46]
Bi-1	Hydrothermal	_Steps:_ 1) Electrodes were thermally treated at 420 °C for 10 h, 2) 0.466 g Bi_2O_3 was added into the solution of 78 mL ethylene glycol and 22 mL ethanol and sonicated for 30 mins, 3) Solution with felt was kept in an autoclave at 200 °C for 6 h, 4) Washed several times with deionized, and 5) Dried at 60 °C in an oven<br>_PU:_ Furnace (2), Mixer, Sonicator, Autoclave, Washer (2), Oven	5/8/13	[47]
Bi-2	Impregnation	_Steps:_ 1) 48 cm^2 of felts was impregnated inro the mixed solution of 60 mg $BiCl_3$ dissolved in 20 mL 5% HCl and 5 mL ethanol with ultrasonic treatment for 0.5 h, 2) Dried at 100 °C for 8 h, 3) Heat treated at 600 °C for 2 h in a mixture of N_2 and H_2 (4:1 volume by volume ratio)<br>_PU:_ Mixer, Ultrasonicator, Oven, Furnace	3/4/7	[48]
Bi-3	Electrodeposit ion during charging	_Steps:_ 1) 0.005 M $BiNO_3$ added to the negative electrolyte<br>_PU:_ None	1/0/1	[49]
Bi-4	KOH Etching and electrodepositi on during charging	_Steps:_ 1) Felt immersed in 5 wt% KOH solution in a beaker (mass ratio of KOH/CF mixture is 1.25), 2) Vacuum dry at 70 °C for 24 h, 3) Calcined at 800 °C for 2 h in N_2 furnace, 4) Rinsed with 0.5 M HCl and water to remove potassium species, 5) 0.005 M $BiNO_3$ added to the negative electrolyte<br>_PU:_ Mixer (2), Oven, Furnace	5/4/9	[49]
Bi-5	Impregnation	_Steps:_ 1) 0.076 g of $BiCl_3$ dissolved in 20 mL 5wt% HCl and 5 mL ethanol, 2) Impregnated on 48cm^2 CF, 3) Dried at 100 °C for 8 h, and 4) Thermally treated at 600 °C for 2 h in a mixture of N_2:H_2 (5:3= v:v)<br>_PU:_ Mixer, Spray coater, Oven, Furnace	4/4/8	[50]
Bi-6	Electrodeposit ion during charging	_Steps:_ 1) 10 mM $BiCl_3$ added to the electrolyte<br>_PU:_ None	1/0/1	[51]
Cu	Electrodeposit ion during charging	_Steps:_ 1) 5 mM $CuSO_4$ added to the electrolyte<br>_PU:_ None	1/0/1	[52]
Ag	Electrodeposit ion during charging	_Steps:_ 1) 21 mg silver nitrate in 50 mL electrolyte (for 12 cm^2 felt area)<br>_PU:_ None	1/0/1	[53]
Sn	Electrodeposit ion during charging	_Steps:_ 1) 10 mM $SnCl_2$ added to the electrolyte<br>_PU:_ None	1/0/1	[54]
Sb	Electrodeposit ion during charging	_Steps:_ 1) 5 mM $SbCl_3$ added to the electrolyte that electrodeposits<br>_PU:_ None	1/0/1	[55]
**Metal oxides**				

ID	Deposition Method	Treatment Details	No. of Steps/PU/ Total	Ref.
IrO_2	Impregnation	*Steps:* 1) Thermal treatment at 400 °C for 30 h, 2) Immersed in 10 wt% ethanol solution of H_2IrCl_6 for 2 mins, 3) Dried in vacuum, 4) Thermally treated in air at 450 °C for 15 mins, and 5) Repeat steps 1-4 eight times<br>*PU:* Furnace (2), Mixer, Oven,	32/4/36	[56]
ZrO_2	Impregnation	*Steps:* 1) Thermally treated in air at 420 °C for 10 h, 2) Felt immersed in a precursor made by dissolving Zr $(NO_3)_4.5H_2O$ in water, 3) This is followed by addition of ammonia until pH was 8, 4) Dried at 70 °C for 12 h, 5) Heated at 500 °C for 5 h under N_2 flow<br>*PU:* Furnace (2), Mixer, pH meter, Oven	5/5/10	[57]
SnO_2	Hydrothermal	*Steps:* 1) 8 mM of $SnCl_4.4H_2O$ was added to 1:1 mixture of ethanol and water and stirred for 30 mins at room temperature, 2) 2 M NaOH was added drop by drop until pH of 12 was reached and stirred for 15 mins, 3) 25cm^2 felt was immersed and hydrothermal reaction at 150 °C for 4 h was conducted, 4) Washed with deionized water, 5) Dried overnight, 6) Thermally treated at 400 °C for 3 h in air<br>*PU:* Mixer (2), pH Meter, Autoclave, Furnace (2), Washer, Oven	6/8/14	[58]
NiO	Impregnation	*Steps:* 1) Felt cleaned by ultrasonication in acetone for 10 mins, 2) This is followed by drying at 60 °C for 1 h in vacuum oven, 3) Nickel nitrate was dissolved in acetone and ultrasonicated for 5 mins, 4) Felt was immersed in 5 wt% nickel solution for 2 h, 5) Dried at 60 °C in vacuum oven for 1.5 h, and 6) Thermally treated at 300 °C for 30 mins in Ar<br>*PU:* Ultrasonicator (2), Oven (2), Mixer, Furnace	6/6/12	[59]
Co_3O_4-1	Pulse	*Steps:* 1) Felt ultrasonically cleaned in ethanol and water, 2) Dried at 70 °C in oven, 3) Annealed at 450 °C in air for 3 h, 4) 0.05 M cobalt acetate solution was used and −1 V for 30 seconds after every 5 seconds interval was applied vs SCE (total time 600 seconds), 5) Washed with deionized water, 6) Dried at 70 °C for 6 h, and 7) Thermally treated at 300 °C for 1 h and at 400 °C for 2 h (calcination)<br>*PU:* Ultrasonicator, Oven (2), Furnace (2), Chemically resistant container, Potentiostat, Washer	7/8/15	[60]
Co_3O_4-2	Hydrothermal	*Steps:* 1) Felt ultrasonically cleaned in ethanol and water, 2) Dried at 70 °C in oven, 3) Annealed at 450 °C in air for 3 h, 4) Felt was placed in a Teflon-lined stainless steel autoclave containing 40 mL of 0.05 mol L^{-1} cobalt acetate tetrahydrate, and 5) Treated in a vacuum oven at 120 °C for 12 h<br>*PU:* Ultrasonicator, Oven (2), Furnace (2), Autoclave	5/6/11	[60]
Cr_2O_3	Impregnation	*Steps:* 1) 0.2 g of Chromium nitrate was dissolved in 25 mL absolute ethanol and felt was immersed in it for 2 mins followed by ultrasonication for 30 seconds, 2) Dried in oven, 3) Heat treated in air at 400 °C for 2 h, and 4) Heat treatment at 500 °C for 5 h in N_2 atmosphere to enhance adhesion between graphite felt and Cr atoms<br>*PU:* Mixer, Ultrasonicator, Oven, Furnace (2)	4/5/9	[61]
CoO	Impregnation	*Steps:* 1) Felt immersed in 25 mL absolute ethanol and 0.02 M metal ion solution for 2 mins followed by sonication for 30 seconds, 2) Heated at 400 °C for 2 h in air, and 3) Heated at 500 °C for 5 h in N_2<br>*PU:* Mixer, Ultrasonicator, Furnace (2)	3/4/7	[62]
$NiCoO_2$	Hydrothermal	*Steps:* 1) Pretreated in air at 150 °C for 3 h, 2) 0.02 M (Co:Ni = 2:1) metal ion (cobalt nitrate, nickel chloride) and 0.1 M urea are dissolved in 25 mL absolute ethanol:water mixture (4:1), 3) Hydrothermal treatment in an autoclave at 80 °C for 14 h, 4) Washed with deionized water, 5) Dried in oven, 6) heated at 400 °C for 2 h in air, and 7) Heated at 500 °C for 5 h in N_2 atmosphere<br>*PU:* Furnace (4), Mixer, Autoclave, Washer, Oven	7/8/15	[62]
Ta_2O_5	Hydrothermal	*Steps:* 1) For a 2.5 g of felt, 180 mg (0.75 wt%, optimized) of Ta_2O_5 was dissolved in 10 mL of 0.1 M HF (40%), and the pH was adjusted to 9 by adding 5 mL of 1 M ammonia solution (30%, NH_4OH) to form a white precipitate, 2) The precipitate was dissolved in 1 M ammonia solution and 1 M H_2O_2 (5:1 volume ratio), 3) Mixture was heated into an oil bath at 70 °C for 1 h, 4) Felt was immersed and hydrothermal reaction carried out at 240 °C for 12 h, 5) Washed with water and ethanol, and 6) Dried overnight at 60 °C in air<br>*PU:* Mixer (2), pH meter, Oil bath, Autoclave, Furnace, Washer (2), Oven	6/9/15	[63]
Nd_2O_3	Impregnation	*Steps:* 1) 20 wt% (by the weight of felt) Neodymium Nitrate was dissolved in 10 mL ethanol followed by addition of 3 mL of NH_4OH and felt was immersed, 2) Impregnated felt is placed in an oven and dried at 70 °C for 1 h, and 3) Felts were then annealed at 500 °C for 3 h under N_2 flow<br>*PU:* Mixer (2), Oven, Furnace	3/4/7	[64]
Mn_3O_4	Hydrothermal	*Steps:* 1) Graphite felt put into 2 M manganese acetate solution in a Teflon lined autoclave and heated to 200 °C for 12 h (hydrothermal reaction), 2) Washed with water, 3) Dried under vacuum for 12 h, and 4) Heat treated at 500 °C for 5 h in Ar<br>*PU:* Autoclave, Furnace (2), Washer, Oven	4/5/9	[65]

H: Rutile TiO_2	Hydrothermal	*Steps:* 1) 5 mL Titanium butoxide was added to 600 mL $HCl:H_2O$ solution (1:1 volume ratio) and felt was placed, 2) Hydrothermal reaction at 100 °C, 3) Drying at 60 °C overnight, 4) Annealed in O_2 atmosphere at 500 °C for 4 h, and 5) 5% H_2 in Ar atmosphere annealed at 500 °C for 4 h<br>*PU:* Mixer, Autoclave, Furnace (3), Oven	5/6/11	[66]
$KMnO_4$	Impregnation	*Steps:* 1) Felt soaked in 0.1 M $KMnO_4$ solution for 5 days, 2) Washed with diluted H_2SO_4 and H_2O_2 for 1 min, and 3) Dried in a vacuum oven All concentrations and times are optimized.<br>*PU:* Vessel, Washer (2), Oven	3/4/7	[67]
CeO_2	Impregnation	*Steps:* 1) Pretreated at 420 °C for 10 h in air, 2) 0.2 wt% (with respect to felt) Cerium nitrate solution was prepared, and pH of solution was adjusted to 9 by $NH_3.H_2O$ solution, and felt was immersed, 3) Thermal treatment at 70 °C for 12 h in air, 4) Calcined at 600 °C for 2 h in N_2<br>*PU:* Furnace (2), Mixer, pH meter, Oven	4/5/9	[68]
		**Chemical Reagent**		
**ID**	**Deposition Method**	**Treatment Details**	**No. of Steps/PU/ Total**	**Ref.**
Prussian Blue	Electrodeposition	*Steps:* 1) Felts were washed with 5 wt% HCl to remove impurities, 2) Washed with water and ethanol three times, 3) This was followed by drying at 50 °C for 12 h, 4) 2.5 mM $K_3Fe(CN)_6$ and 2.5 mM $FeCl_3$ was added to 0.5 M KCl solution and several drops of 5 wt% HCl was added to adjust the pH to 2, and 5) Graphite plate was used as counter electrode and 0.1 V (vs Hg/Hg_2SO_4) was applied for 500 seconds to electrodeposit on carbon felt (optimized)<br>*PU:* Washer (3), Oven, Mixer, pH meter, Reaction vessel, Potentiostat	9/8/17	[69]
		**Nanofibers**		
**ID**	**Deposition Method**	**Treatment Details**	**No. of Steps/PU/ Total**	**Ref.**
CeO_2: Espun Nanofiber	Electrospinning	*Steps:* 1) 12 wt% polyacrylonitrile (PAN) was dissolved in DMF and kept stirring at 80 °C for 6 h, 2) Cerium ammonium nitrate and PAN (mass ratio 1:10) were added to PAN/DMF solution and kept for stirring at room temperature for 10 h, 3) Electrospinning at positive direct voltage of 20 kV, 4) Electrospun fibers were preoxidized at 280 °C for 30 mins in air, and 5) Carbonization at 1000 °C in N_2 flow<br>*PU:* Mixer (2), Electrospinning equipment, Voltage supply, Furnace (2)	5/6/11	[70]
TiO_2: Espun Nanofiber	Electrospinning	*Steps:* 1) Polyacrylonitrile (PAN) fiber dissolved in DMF to form 10 mL of 10 wt% PAN/DMF solution followed by agitation for 10 mins, 2) 1 mL $Ti(OC_4H_9)$[TnBT] is added in this solution with stirring at 70 °C for 4 h, 3) Electrospinning at 10 kV positive voltage and 3.5 kV negative voltage, 4) Protoxidized fibers were treated at 270 °C for 1 h in air, and 5) Heating at 900 °C for 2 h in Ar atmosphere<br>*PU:* Mixer (2), Electrospinning equipment, Voltage supply, Furnace (2)	5/6/11	[71]
		**Metal Oxide (or doped) in HCl**		
**ID**	**Deposition Method**	**Treatment Details**	**No. of Steps/PU/ Total**	**Ref.**
Nb_2O_5	Hydrothermal	*Steps:* 1) 0.1 M ammonium niobium oxalate (and ammonium paratungstate) were mixed with water, the mole ratio of W:Nb was kept 1:10, 2) Solution placed for hydrothermal treatment with felt at 170 °C for 48 h, 3) Precursor powders were collected by vacuum filtration, 4) Washed thrice with deionized water, and 5) Dried and calcined at 500 °C in Ar flow for 2 h<br>*PU:* Mixer, Autoclave, Furnace, Filter, Washer, Furnace	5/6/11	[72]
Nb_2O_5-W			5/6/11	[72]

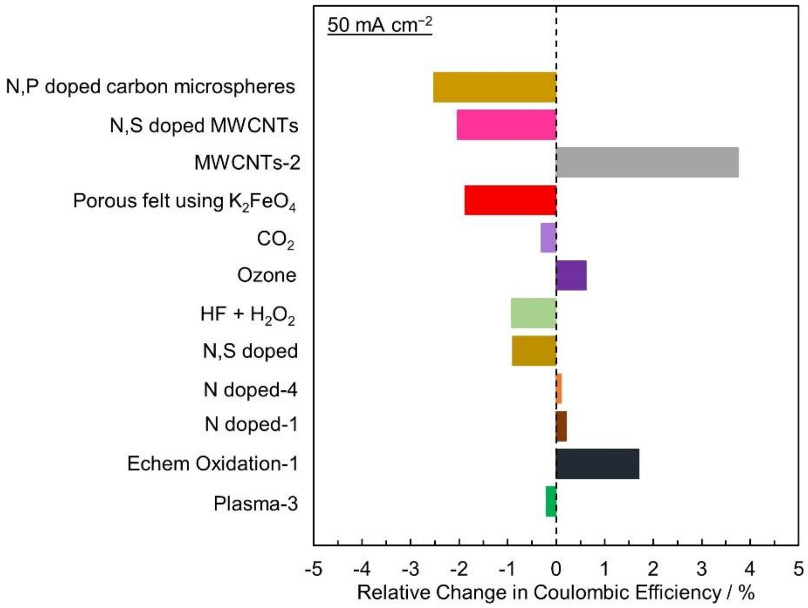

Figure S1. Relative change in Coulombic Efficiency for VRFBs with carbon felts with and without treatments at fixed current density of 50 mA cm^{-2}. Only the treatments for which Coulombic Efficiency at 50 mA cm^{-2} is reported are considered. The change in Coulombic Efficiency after treatments is < 5 % for VRFBs.

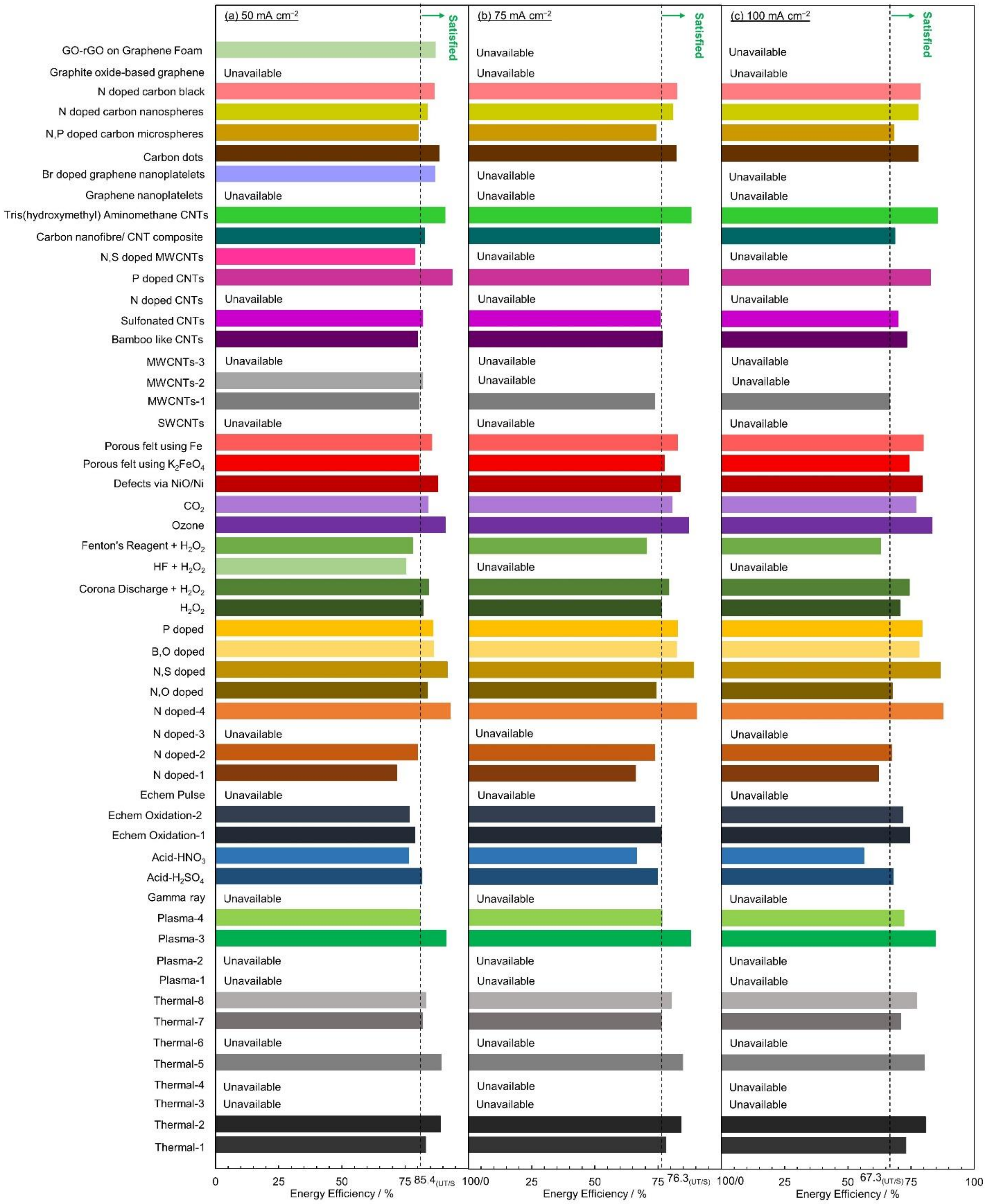

Figure S2. Energy Efficiency of laboratory scale VRFB with various carbon felt treatments at three different current densities. **(a)** 50, **(b)** 75, and **(c)** 100 mA cm^{-2}. The Energy Efficiency for a scaled up (200 kW/ 400 kWh) VRFB with untreated carbon felt (UT/S) as electrodes is shown by dotted line for each current density. If the Energy Efficiency of laboratory scale VRFB for at least one of the three current densities exceed Energy Efficiency of VRFB with UT/S,

first part of the performance criteria as discussed in the main text is satisfied. The green arrow highlights the region where energy efficiency should lie for each current density to satisfy the criteria.

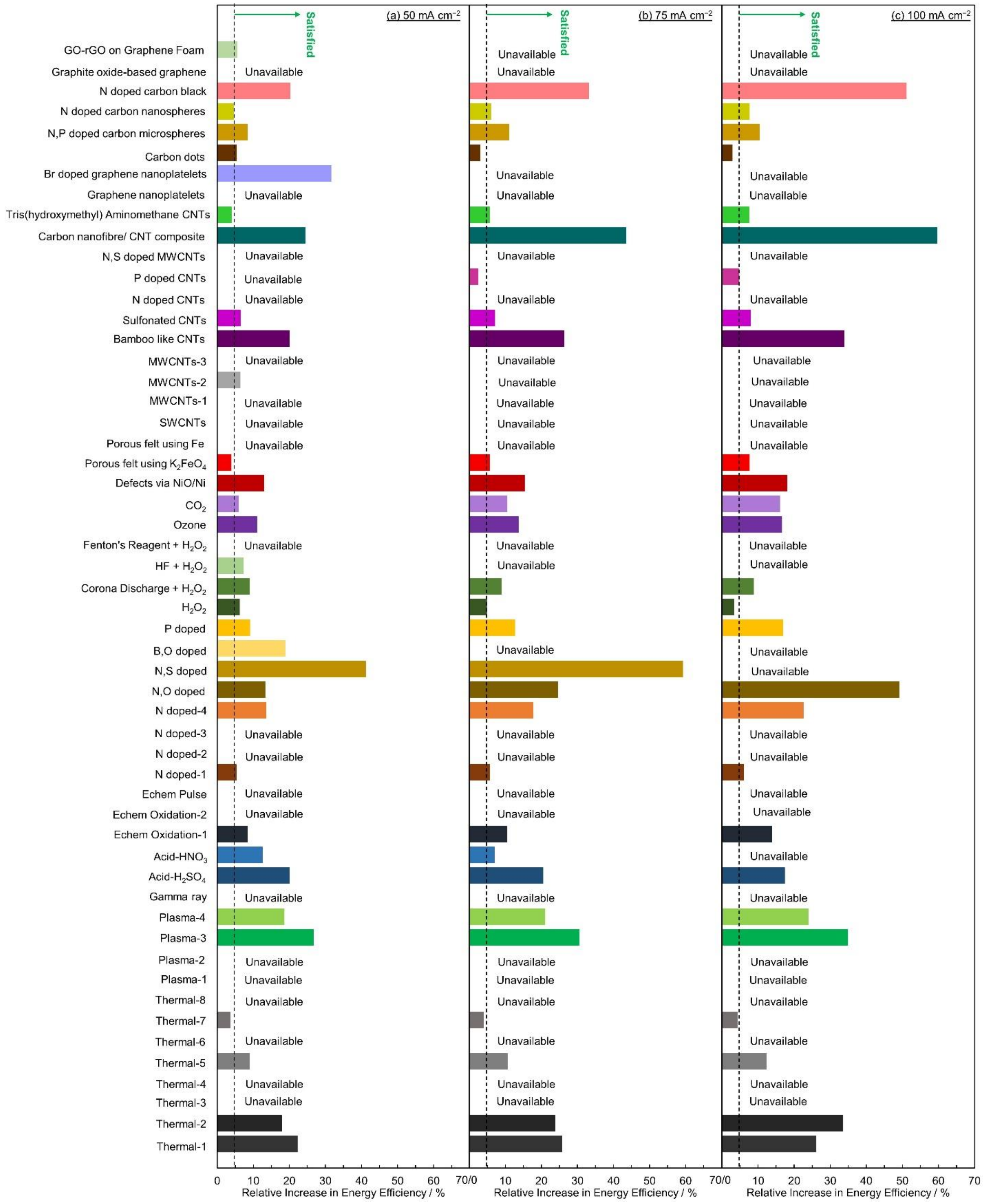

Figure S3. Relative increase in Energy Efficiency of laboratory scale VRFB with various carbon felt treatments at three different current densities. **(a)** 50, **(b)** 75, and **(c)** 100 mA cm^{-2}. If the relative increase in Energy Efficiency of

laboratory scale VRFB for at least one of the three current densities is greater than 5 %, second part of the performance criteria as discussed in the main text is satisfied. The green arrow highlights the region where relative increase in energy efficiency should lie for each current density to satisfy the criteria.

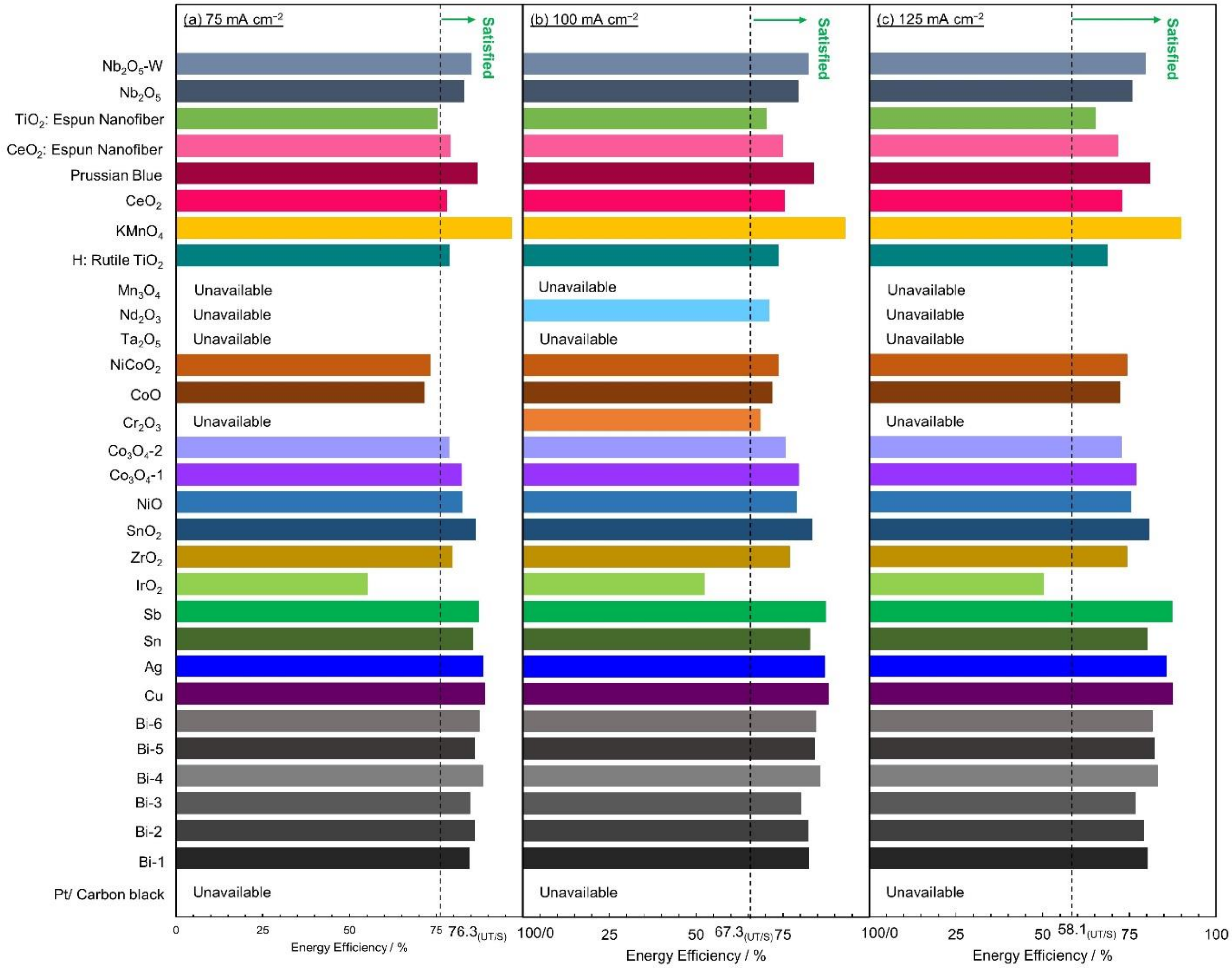

Figure S4. Energy Efficiency of laboratory scale VRFB with different metal and metal oxide based electrocatalysts at three different current densities. **(a)** 75, **(b)** 100, and **(c)** 125 mA cm^{-2}. The Energy Efficiency for a scaled up (200 kW/ 400 kWh) VRFB with untreated carbon felt (UT/S) as electrodes is shown by dotted line for each current density. If the Energy Efficiency of laboratory scale VRFB for at least one of the three current densities exceed Energy Efficiency of VRFB with UT/S, first part of the performance criteria as discussed in the main text is satisfied. The green arrow highlights the region where energy efficiency should lie for each current density to satisfy the criteria.

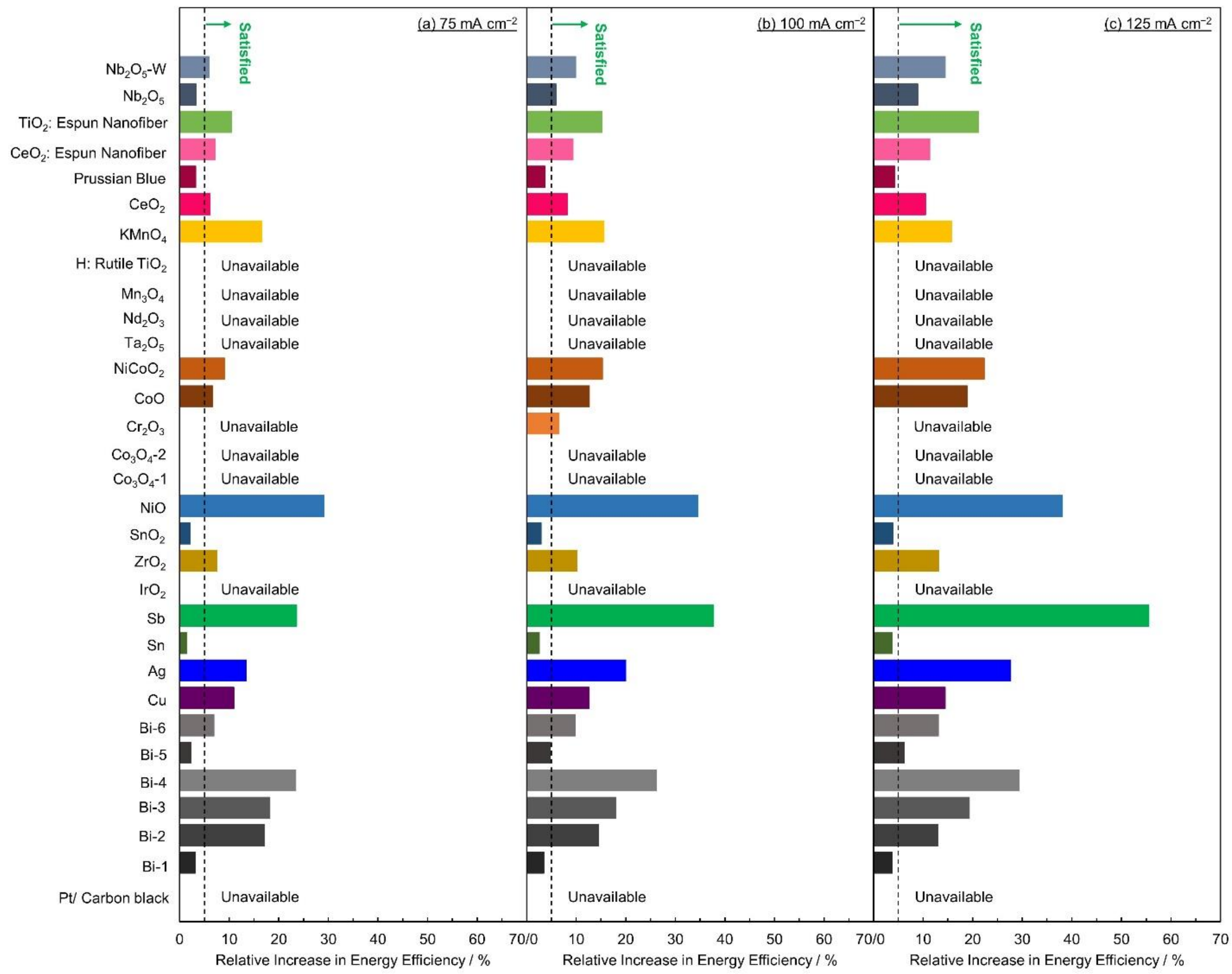

Figure S5. Relative increase in Energy Efficiency of laboratory scale VRFB with different metal and metal oxide based electrocatalysts at three different current densities. **(a)** 75, **(b)** 100, and **(c)** 120 mA cm^{-2}. If the relative increase in Energy Efficiency of laboratory scale VRFB for at least one of the three current densities is greater than 5 %, second part of the performance criteria as discussed in the main text is satisfied. The green arrow highlights the region where relative increase in energy efficiency should lie for each current density to satisfy the criteria.

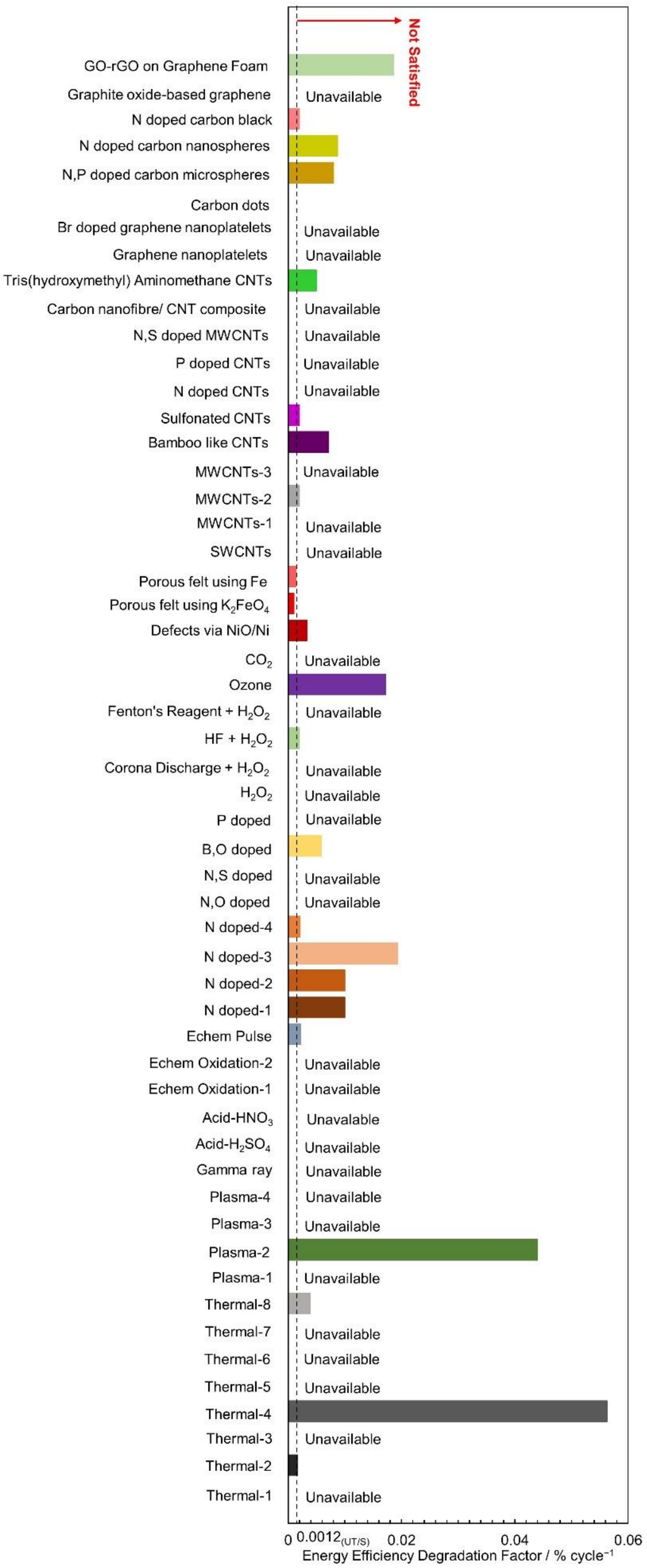

Figure S6. Energy Efficiency Degradation Factor for laboratory scale VRFB with various carbon felt treatments. If the Energy Efficiency Degradation Factor of laboratory scale VRFB is < 0.0012 % cycle^{-1}, stability criteria as discussed in the main text is satisfied. The red arrow highlights the region where Energy Efficiency Degradation Factor should *not* lie for to satisfy the criteria.

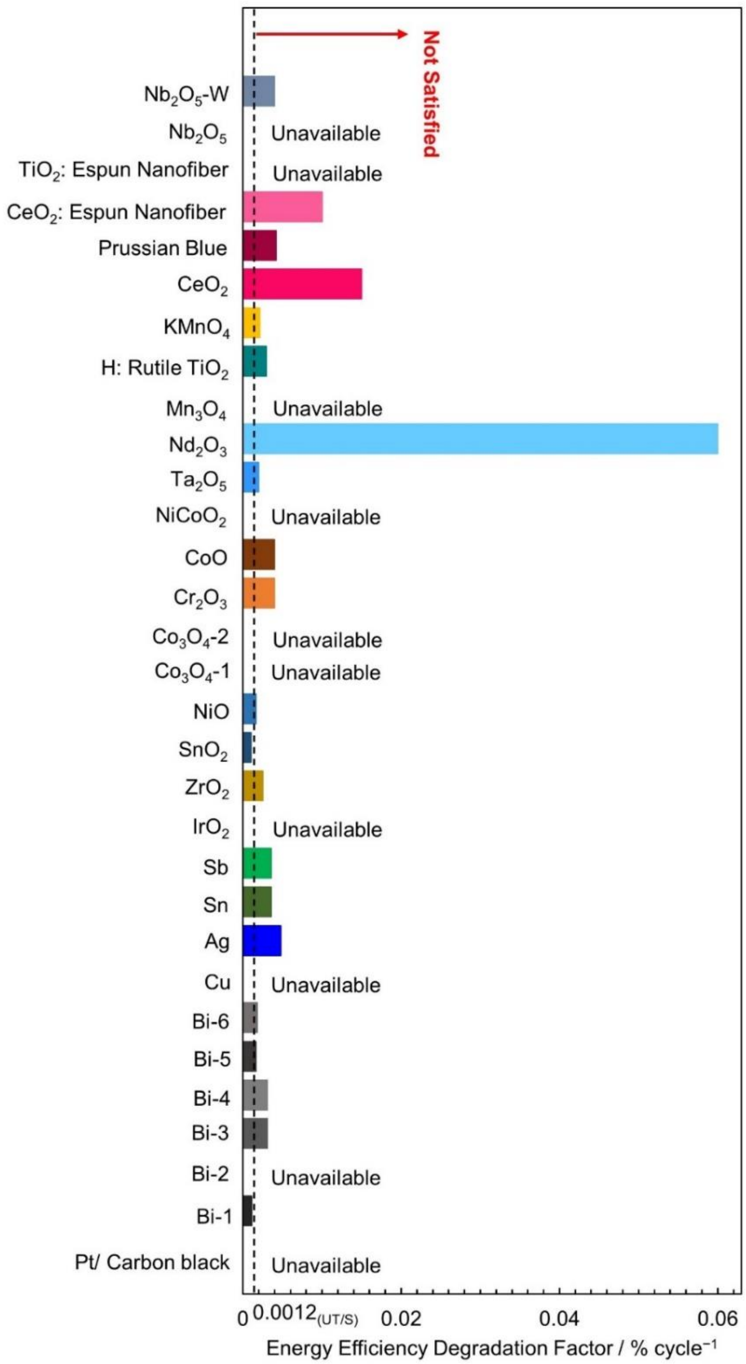

Figure S7. Energy Efficiency Degradation Factor for laboratory scale VRFB with various metal and metal-oxide electrocatalysts. If the Energy Efficiency Degradation Factor of laboratory scale VRFB is < 0.0012 % cycle^{-1}, stability criteria as discussed in the main text is satisfied. The red arrow highlights the region where Energy Efficiency Degradation Factor should *not* lie for to satisfy the criteria.

S2. Techno-economic Model for Evaluating Affordable Capital Cost

An in-house technoeconomic model is developed to evaluate the total overall capital expenditure (CAPEX) of the flow battery. The current density vs energy efficiency relationships developed for various treatments are used to obtain the number of stacks required to deliver a fixed capacity of 12 MWh (**Table S5**). Since the number of stacks required to deliver a fixed capacity changes with operating current density, as shown in **Table S5**, the CAPEX for VRFBs with treated and untreated carbon felts are different.

Table S5. Battery key metrics, design factors, and efficiencies considered in the techno-economic model.

Parameters	Value	Unit	Notes
Battery Key Metrics			
Discharge time	8	h	Assumed and kept fixed
Power	1500	kW	Assumed and kept fixed
Energy capacity	12	MWh	Discharge Time × Power
Capacity utilization factor	0.8		Assumed and kept fixed
Energy capacity assuming 80% utilization	15	MWh	(Energy capacity / Capacity utilization factor) × 100
Battery Design Factors			
No. of cells per stack	27		Assumed and kept fixed
No. of bipolar plates per stack	26		Assumed and kept fixed
Width of the stacks	0.4	m	Assumed and kept fixed
Length of cell	0.3	m	Assumed and kept fixed
Cell size	0.12	m^2	Width of stack × Length of cell
Length of auxiliary pipe	0.3	m	Assumed and kept fixed
No. of stacks in a row	12		Assumed and kept fixed
No. of current collectors every 10 cells	1		Assumed and kept fixed
Width of current collector	0.005	m	Assumed and kept fixed
Volume of current collector	0.0006	m^3	Cell size × Width of current collector
Cell voltage	1.26	V	Cell Voltage of VRFB
Battery Efficiencies			
Current density			Obtained from energy efficiency vs current density relationships and varied
Round trip efficiency	67.3	%	Base case of 67.3 % and varied
Total energy obtained	8.04	MWh	Energy capacity × Round trip efficiency × 0.01
Power density		W/ cell nominal	Cell voltage × Current density × Cell size × No. of cells associated with one current collector
No. of stacks			(Power × 10^3) / (Power density × No. of cells per stack)

The cost associated with balance of plant hardware, electrolyte, and utilities are unchanged with the change in number of stacks for an RFB with fixed energy capacity. Thus, the total CAPEX for the flow battery system with fixed energy capacity is the sum of the cost of battery stack and balance of plant hardware component evaluated in **Table S6**. The CAPEX for VRFBs with treated and untreated CFs (UT and UT/S) are evaluated to obtain the affordable capital cost (ACC_{UT} and $ACC_{UT/S}$), using the methodology discussed in **Figure 6** of main text.

Table S6. Contributions of different components to CAPEX. (a) Battery Stack and (b) Balance of Plant – Hardware. Here, "I" in references column represent cost estimates obtained from internal models at Shell.

(a) *Battery Stack*						
**S. No.**	**Component**	**Cost ($/Unit)**	**Unit**	**$/System**	**Notes**	**Ref.**
1	End Plates	810	m^2	Cost ($/Unit) × 2 × Cell size × No. of stacks	2 end (or isolation) plates per stack Assuming exchange rate of EUR to USD as 1.2, Assuming Germany location factor of 0.83	[73]
2	Isolation Plate	434	m^2	Cost ($/Unit) × 2 × Cell size × No. of stacks		[73]
3	Copper current collector	1006	m^2	(Cost ($/Unit) × Cell Size × No. of cells per stack × No. of stacks) / No. of cells associated with one current collector	1 current collector every 10th cell	[73]
4	Tie rods	13.65	piece	Cost ($/Unit) × 4 × No. of stacks	4 tie rods per stack	[74]
5	Gasket (stack)	563	m^2	Cost ($/Unit) × 2 × Cell size × No. of stacks	2 PVC gaskets per stack Assuming exchange rate of EUR to USD as 1.2, Assuming Germany location factor of 0.83	[73]
6	Carbon felt	77	m^2	Cost ($/Unit) × 2 × Cell size × No. of cells per stack × No. of stacks	2 carbon felts per cell	[75,76]

7	Cell frame	78	m^2	Cost ($/Unit) × Cell Size × No. of cells per stack × No. of stacks	1 frame per cell	[73,75,76]
8	Membrane (Nafion)	451	m^2	Cost ($/Unit) × Cell Size × No. of cells per stack × No. of stacks	1 membrane per cell	[75]
9	Bipolar plate	145	m^2	Cost ($/Unit) × Cell Size × No. of bipolar plates per stack × No. of stacks	Graphite bipolar plates	[75]
10	Container	7478.77	piece	(Cost ($/Unit) × No. of stacks) / 45	Assume 45 stacks/ container	[77]
**Battery stack cost per system**				**Sum of S. No. 1-10**		
**(b) *Balance of Plant – Hardware***						
**S. No.**	**Component**	**Cost ($/Unit)**	**Unit**	**$/System**	**Notes**	**Ref.**
1	Large Diameter pipe (Main line)	33.6	m	Cost ($/Unit) × X, where: X = (Width of the stacks × No. of stacks × 4) + (1.5+ (No. of stacks in a row × Width of the stacks)/2) + (2 × Length of cell) + (Length of cell × 1.5 × 2 × Y); Y = (No. of stacks – No. of stacks in row)/ No. of stacks in row	30 cm diameter, plastic pipe (HDPE or PVC). PVC Schedule 80 Pipe. Max. Working pressure of 1.6 MPa	[78]
2	Small Diameter pipe (Auxiliary line)	3.24	m	Cost ($/Unit) × Length of auxiliary pipe × No. of stacks × 4	6 cm diameter, plastic pipe (HDPE or PVC) PVC Schedule 80 Pipe. Max. Working pressure of 2.9 MPa.	[78]
3	Pump	46.67	kW	Cost ($/Unit) × Maximum Pump Power × 3	120 m head and 140 L/s flow rate, 150 kW pump is $7000 per pump so this gives the price per kW. 3 pumps because one for anolyte, one for catholyte and another extra	I
4	Tee-Reducer	148.3	piece	Cost ($/Unit) × No. of stacks × 4	4 Tee-reducers per stack (anolyte & catholyte). PVC schedule 40 fittings. Size no. 580: 20 cm x 20 cm x 7.5 cm	[78]
5	Elbows (Auxiliary line)	15.03	piece	Cost ($/Unit) × No. of stacks × 6	Elbows taken for stacks (anolyte, catholyte, and water lines for heat-exchange). PVC Schedule 80: Part No. 3806 (2.5" size)	[78]
6	Elbows (Main line)	300	piece	Cost ($/Unit) × (((No. of stacks/ No. of stacks in a row −1) × 4) + 14)	Large elbows are determined by how many rows of stacks there are. PVC Schedule 80. Part No. 3806 (8" price scaled)	[78]
7	Instrumentation: Pressure Transmitter	311.94	piece	Cost ($/Unit) × No. of stacks × 2	Pressure range from 0-10000 psi. WIKKA A-10 pressure transmitter (Size no. 500)	[78]
8	Instrumentation: Temperature Transmitter	304	piece	Cost ($/Unit) × No. of stacks	Signet 2350 temperature sensor	[78]
9	Instrumentation: Flow Sensor	380	piece	Cost ($/Unit) × No. of stacks × 2	6 m/s max flow rate. Rotor-X Paddlewheel flow sensor	[78]
10	Manometers (local pressure gauges)	100	piece	Cost ($/Unit) × No. of stacks	To be placed at the stacks	I
11	Frames	25	kW	Cost ($/Unit) × Power	Frames for stacks to be mounted on	I
12	Valves (Main Line)	3092	piece	Cost ($/Unit) × 4	Type 240 Pneumatically actuated butterfly valve. Part no. 54309	[78]
13	Valves (Auxiliary Line)	75	piece	Cost ($/Unit) × No. of stacks × 6	6 valves per stack: 4 for anolyte/catholyte streams and 2 for water line for the heat exchanger. Ball Valve: Order no. 5027 PVC/EPDM COLORO Size no. 2, socket valve, 252 Psi. Price adjusted for 6" size	[78]
14	Control Panel	20	kW	Cost ($/Unit) × Power		I
15	Heat Exchanger	84	kW	Cost ($/Unit) × Power		[76]
**Battery balance of plant – Hardware cost per system**				**Sum of S. No. 1-15**		

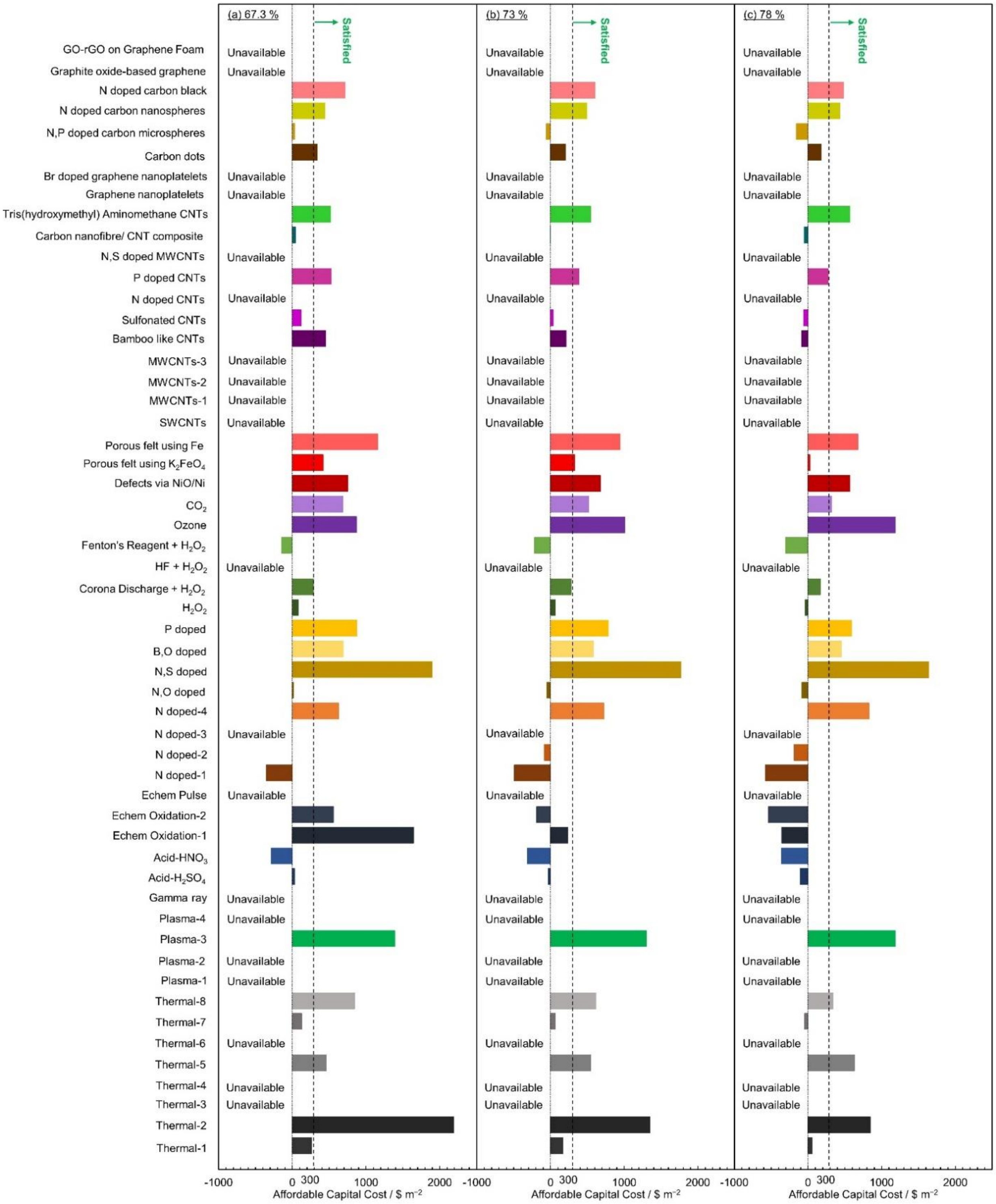

Figure S8. Affordable Capital Cost for laboratory scale VRFB with various carbon felt treatments at energy efficiencies of **(a)** 67.3, **(b)** 73, and **(c)** 78 %. The green arrow highlights the region where affordable capital cost should lie for each energy efficiency to satisfy the criteria.

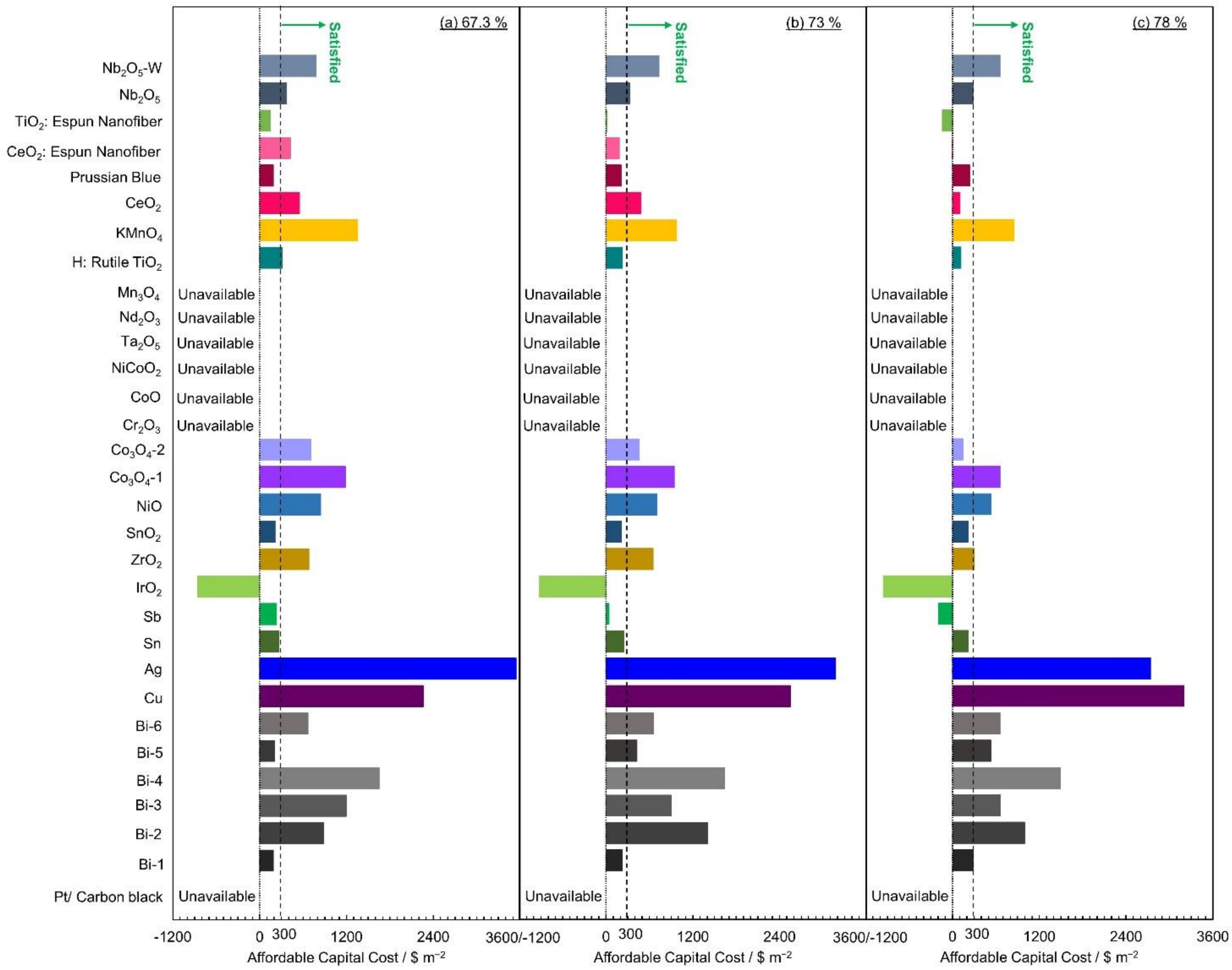

Figure S9. Affordable Capital Cost for laboratory scale VRFB with various metal and metal-oxide electrocatalysts at energy efficiencies of **(a)** 67.3, **(b)** 73, and **(c)** 78 %. The green arrow highlights the region where affordable capital cost should lie for each energy efficiency to satisfy the criteria.

S3. Cost Estimate of Carbon Felts and Treatments

The costs of carbon felts (CFs) are evaluated using the cost of polyacrylonitrile (PAN) precursor and adding the costs associated with textile and pressing. All calculations are done assuming total production of carbon fiber is 1500 ton year^{-1}.

1) <u>Carbon Felts</u>:

Cost of PAN precursor[79] ($C_{Precursor}$) = 5.9 \$ lb^{-1} ~13 \$ kg^{-1} (using 1 lb = 0.454 kg)
Ratio of textile processing cost to PAN precursor cost, taken from ref.[80] = $\frac{0.59}{0.41}$ = 1.44 (as of 2017)
Cost of textile processing per kg of PAN precursor ($C_{Textile\ Processing}$) = 1.44 × 13 = 18.70 \$ kg^{-1}
Ratio of carbonization cost to PAN precursor cost, taken from ref.[80] = $\frac{0.69}{0.41}$ = 2.22 (as of 2017)
Cost of carbonization per kg of PAN precursor ($C_{Carbonization}$) = 2.22 × 13 = 28.93 \$ kg^{-1}
Yield of CF from carbon fibers[80] ($Y_{CF\ from\ fibers}$) = 0.5
Total cost of making CFs, taken from ref.[80]

$$= \frac{(C_{Precursor} + C_{Textile\ Processing} + C_{Carbonization})}{Y_{CF\ from\ fibers}} = \frac{(13 + 18.70 + 28.93)}{0.5} = 121.2\ \$\ kg^{-1}$$

Assuming a yearly lumpsum inflation of 2 %, costs of making CFs as of 2022

$$= 121.2 \times (1 + 0.02)^5 = 133.8\ \$\ kg^{-1}$$

Using the (Weight/Area) ratio of 6 mm thick CFs[81] = 0.5 kg m^{-2}, we get the cost per m^2 of CF

$$= 0.5\ kg\ m^{-2} \times 133.8\ \$\ kg^{-1} = 66.9\ \$\ m^{-2}$$

2) ***Carbon Felt Treatments:***

a) Thermal:

The oxidation stage in carbon fiber preparation occurs between 200 – 300 °C for 1.5 – 2 hours.[79,82]
Assuming the lower temperature of 200 °C is used for carbon fiber treatment for 1.75 h:

Duty of carbon fiber treatment ($Duty_{fiber}$) = ($T_{fiber} - T_{room\ temp}$) × Time

$$= (200-25)\ °C \times 1.75\ h = 306.25\ °C\ h$$

Estimated costs of thermal treatment of carbon fibers by researchers at Oak Ridge National Lab,[79,83–85] as of 2013 = 1.78 \$ (lb of PAN precursor)$^{-1}$ ~ 3.92 \$ (kg of PAN precursor)$^{-1}$
Using a lumpsum inflation of 2 %, cost of thermal treatment of carbon fiber as of 2022 = $3.92 \times (1 + 0.02)^9$ = 4.68 \$ (kg of PAN precursor)$^{-1}$
Using $Y_{CF\ from\ fibers}$ and (Weight/Area) ratio of 6 mm CFs, we get cost of CFs ($Cost_{CF,ref}$)

$$= \frac{\left(\frac{4.68\ \$}{\text{kg of PAN precursor}} \times 0.5 \frac{kg}{m^2}\right)}{0.5} = 4.68\ \$\ m^{-2}$$

ID Thermal-2:
The CF is treated at 750 °C for 5 mins in ID *Thermal-2* treatment (**Table S3**). We scale the cost based on the change in energy required, estimated by the change in duty.
Duty associated with *Thermal-2* ($Duty_{Thermal-2}$) = ($T_{Thermal-2} - T_{room\ temp}$) × Time

$$= (750-25)\ °C \times (5/60)\ h = 60.42\ °C\ h$$

Cost of *Thermal-2* treatment = $\frac{Duty_{Thermal-2}}{Duty_{fiber}} \times Cost_{CF,ref,Thermal} = \frac{60.42}{306.25} \times 4.68 = 0.9$ \$ m^{-2}

ID Thermal-5:
The CF is treated at 500 °C for 5 h in ID *Thermal-5* treatment (**Table S3**). Using the process same as for ID *Thermal-2*, we get:
Cost of *Thermal-5* treatment = $\frac{Duty_{Thermal-5}}{Duty_{fiber}} \times Cost_{CF,ref,Thermal} = \frac{(500-25)\times 5}{306.25} \times 4.68 = 36.3$ \$ m^{-2}
The difference between the cost of untreated and thermally treated CF of same thickness from SGL Carbon is ~ 30-40 € m^{-2} (**Figure S10**). Using € to \$ conversion ratio as 1.18, this cost difference lies between ~ 35-47 \$ m^{-2}, which is close to the cost of *Thermal-5* treatment evaluated above.

Global price list

SIGRACELL® battery felts

Small-quantity pricing for end-customers

Sheets

Size: 210 x 297 mm
Minimum order value for sheets: 250,– €

Grade	€/sheet
KFD 2.5 EA	50
GFD 2.5 EA	50
GFD 4.6 EA	50
GFA 6 EA	50
GFD 2.5 EA thermally activated	85
GFD 4.6 EA thermally activated	85

For quantities > 60 sheets and for customized cut-to-size material please contact us for an individual quotation.

Roll materials

Minimum order value for roll materials: 500,– €

Grade	Roll width (mm)	€/m²
KFD 2.5 EA	1200	100
GFD 2.5 EA	1200	120
GFD 4.6 EA	1200	150
GFA 6.0 EA	1270	170
GFD 2.5 EA thermally activated	1200	150
GFD 4.6 EA thermally activated	1200	180

Prices for roll material are valid for an order quantity up to 15 m². For larger quantities please contact us for an individual quotation.

Figure S10. Screenshot of the quote from SGL Carbon for thermally treated carbon felts, obtained in July 2021. 2.6 and 4.6 mm (2.6 EA and 4.6 EA) are two thicknesses in which thermally treated carbon felts are sold by SGL Carbon.

b) <u>Plasma:</u>

Carbon fibers are treated in plasma ~20-24 mins (t_{fiber}, assuming an average of 22 mins for calculations).[85]

Estimated costs of plasma treatment of carbon fibers by researchers at Oak Ridge National Lab,[79,83–85] as of 2013 = 2.04 \$ (lb of PAN precursor)$^{-1}$ ~ 4.49 \$ (kg of PAN precursor)$^{-1}$

Using a lumpsum inflation of 2 %, cost of plasma treatment of carbon fiber as of 2022 = $4.49 \times (1+0.02)^9$ = 5.36 \$ (kg of PAN precursor)$^{-1}$

Using $Y_{CF\ from\ fibers}$ and (Weight/Area) ratio of 6 mm CFs, we get cost of CFs ($Cost_{CF,ref,Plasma}$)

$$= \frac{\left(5.36 \frac{\$}{\text{kg of PAN precursor}} \times 0.5 \frac{kg}{m^2}\right)}{0.5} = 5.36\ \$\ \text{m}^{-2}$$

ID Plasma-3:

The CFs is treated with plasma for 10 mins ($t_{Plasma-3}$) in ID *Plasma-3* treatment (**Table S3**). We scale the cost based on the change in time of plasma treatment.

Cost of *Plasma-2* treatment = $\frac{t_{Plasma-3}}{t_{fiber}} \times Cost_{CF,ref,Plasma} = \frac{10}{22} \times 5.36 = 2.4\ \$\ \text{m}^{-2}$

c) <u>Electrochemical Oxidation:</u>

The surface treatment for carbon fibers involves passing 500 C g^{-1} (= 139 mAh g^{-1}) of charge.[86]

Estimated costs of surface treatment of carbon fibers by researchers at Oak Ridge National Lab,[79,83–85] as of 2013 = 0.80 \$ (lb of PAN precursor)$^{-1}$ ~ 1.76 \$ (kg of PAN precursor)$^{-1}$.

Using a lumpsum inflation of 2 %, cost of surface treatment of carbon fiber as of 2022 (C_{total}) = $1.76 \times (1+0.02)^9$ = 2.10 \$ (kg of PAN precursor)$^{-1}$

Using $Y_{CF\ from\ fibers}$ and (Weight/Area) ratio of 6 mm CFs, we get cost of CFs ($Cost_{CF,ref,surface\ treatment}$)

$$= \frac{\left(2.10 \frac{\$}{\text{kg of PAN precursor}} \times 0.5 \frac{kg}{m^2}\right)}{0.5} = 2.10\ \$\ \text{m}^{-2}$$

ID Echem Oxidation-1:

The CFs is electrochemically oxidized in 1 M H_2SO_4 passing ~2050 C g^{-1} of charge in ID *Echem Oxidation-1* treatment (**Table S3**). We scale the cost based on the change in charge passed for carbon fibers surface treatment and *Echem Oxidation-1* treatment.

Cost of *Echem Oxidation-1* treatment = $\frac{2050\ C\ g^{-1}}{500\ C\ g^{-1}} \times Cost_{CF,ref,surface\ treatment}$

$= \frac{2050}{500} \times 2.10 = 8.6$ \$ m^{-2}

d) <u>CO_2 treatment:</u>

The cost of CO_2 treatment is estimated based on the estimated cost of *Thermal-5* treatment above, considering the difference in specific heat capacities and price of air and CO_2. The CFs are treated at 1000 °C for 0.5 h in ID *CO_2* (**Table S3**).

Duty associated with *CO_2* ($Duty_{CO_2}$) = ($T_{CO_2} - T_{room\ temp}$) × Time

= (1000−25) °C × 0.5 h = 487.5 °C h

Specific heat capacity of air (S_{air})[87] = 1.005 J g^{-1} K^{-1}

Cost of air ($Cost_{air}$)[88] = 0.111 \$ ft^{-3}

Specific heat capacity of CO_2 (S_{CO_2})[89]= 0.84591 J g^{-1} K^{-1}

Cost of CO_2 ($Cost_{CO_2}$)[88] = 0.181 \$ ft^{-3}

Cost of *CO_2* treatment = $\frac{Duty_{CO_2}}{Duty_{Thermal-5}} \times \frac{S_{CO_2}}{S_{air}} \times \frac{Cost_{CO_2}}{Cost_{air}} \times Cost_{Thermal-5}$

$= \frac{487.5}{(500-25)\times 5} \times \frac{0.84591}{1.005} \times \frac{0.181}{0.111} \times 35.6 = 10.03$ \$ m^{-2}

REFERENCES:

[1] B. Sun, M. Skyllas-Kazacos, *Electrochim. Acta* **1992**, *7*, 1253.

[2] P. C. Ghimire, R. Schweiss, G. G. Scherer, T. M. Lim, N. Wai, A. Bhattarai, Q. Yan, *Carbon N. Y.* **2019**, *155*, 176.

[3] K. J. Kim, Y.-J. Kim, J.-H. Kim, M.-S. Park, *Mater. Chem. Phys.* **2011**, *131*, 547.

[4] D. Dixon, D. J. Babu, J. Langner, M. Bruns, L. Pfaffmann, A. Bhaskar, J. J. Schneider, F. Scheiba, H. Ehrenberg, *J. Power Sources* **2016**, *332*, 240.

[5] D. Kil, H. June Lee, S. Park, S. Kim, H. Kim, *J. Electrochem. Soc.* **2017**, *164*, A3011.

[6] H. Kim, J. S. Yi, D. Lee, *ACS Appl. Energy Mater* **2021**, *4*, 425.

[7] K. J. Kim, H. S. Lee, J. Kim, M. S. Park, J. H. Kim, Y. J. Kim, M. Skyllas-Kazacos, *ChemSusChem* **2016**, *9*, 1329.

[8] Y. Liu, Y. Shen, L. Yu, L. Liu, F. Liang, X. Qiu, J. Xi, *Nano Energy* **2018**, *43*, 55.

[9] S. Bellani, L. Najafi, M. Prato, R. Oropesa-Nuñ Ez, B. Martín-García, L. Gagliani, E. Mantero, L. Marasco, G. Bianca, M. I. Zappia, C. Demirci, S. Olivotto, G. Mariucci, V. Pellegrini, M. Schiavetti, F. Bonaccorso, *Chem. Mater* **2021**, *33*, 4106.

[10] J. Z. Chen, W. Y. Liao, W. Y. Hsieh, C. C. Hsu, Y. S. Chen, *J. Power Sources* **2015**, *274*, 894.

[11] B. Sun, M. Skyllas-Kazacos, *Electrochim. Acta* **1992**, *37*, 2459.

[12] W. Zhang, J. Xi, Z. Li, H. Zhou, L. Liu, Z. Wu, X. Qiu, *Electrochim. Acta* **2013**, *89*, 429.

[13] X. Li, K. Huang, S. Liu, N. Tan, L. Chen, *Trans. Nonferrous Met. Soc. China (English Ed.* **2007**, *17*, 195.

[14] Z. He, Y. Jiang, H. Zhou, G. Cheng, W. Meng, L. Wang, L. Dai, *Int. J. Energy Res.* **2017**, *41*, 439.

[15] T. Wu, K. Huang, S. Liu, S. Zhuang, D. Fang, S. Li, D. Lu, A. Su, *J. Solid State Electrochem.* **2012**, *16*, 579.

[16] Z. He, L. Shi, J. Shen, Z. He, S. Liu, *Int. J. Energy Res.* **2015**, *39*, 709.

[17] C. Flox, M. Skoumal, J. Rubio-Garcia, T. Andreu, J. R. Morante, *Appl. Energy* **2013**, *109*, 344.

[18] S. Park, H. Kim, *J. Mater. Chem. A* **2015**, *3*, 12276.

[19] M. E. Lee, H. J. Jin, Y. S. Yun, *RSC Adv.* **2017**, *7*, 43227.

[20] A. B. Shah, Y. Wu, Y. L. Joo, *Electrochim. Acta* **2019**, *297*, 905.

[21] K. J. Kim, S. W. Lee, T. Yim, J. G. Kim, J. W. Choi, J. H. Kim, M. S. Park, Y. J. Kim, *Sci. Rep.* **2014**, *4*, 1.
[22] Z. He, Y. Jiang, W. Meng, F. Jiang, H. Zhou, Y. Li, J. Zhu, L. Wang, L. Dai, *Appl. Surf. Sci.* **2017**, *423*, 111.
[23] C. Gao, N. Wang, S. Peng, S. Liu, Y. Lei, X. Liang, S. Zeng, H. Zi, *Electrochim. Acta* **2013**, *88*, 193.
[24] Y. C. Chang, J. Y. Chen, D. M. Kabtamu, G. Y. Lin, N. Y. Hsu, Y. S. Chou, H. J. Wei, C. H. Wang, *J. Power Sources* **2017**, *364*, 1.
[25] J. J. Park, J. H. Park, O. O. Park, J. H. Yang, *Carbon N. Y.* **2016**, *110*, 17.
[26] Y. Lv, Y. Li, C. Han, J. Chen, Z. He, J. Zhu, L. Dai, W. Meng, L. Wang, *J. Colloid Interface Sci.* **2020**, *566*, 434.
[27] W. Li, J. Liu, C. Yan, *Electrochim. Acta* **2012**, *79*, 102.
[28] W. Li, J. Liu, C. Yan, *Carbon N. Y.* **2011**, *49*, 3463.
[29] G. NanotubesWei, C. Jia, J. Liu, C. Yan, *J. Power Sources* **2012**, *220*, 185.
[30] H. Yang, C. Fan, Q. Zhu, *J. Energy Chem.* **2018**, *27*, 451.
[31] Y.-C. Chang, Y.-C. Shih, J.-Y. Chen, G.-Y. Lin, N.-Y. Hsu, Y.-S. Chou, C.-H. Wang, *RSC Adv.* **2016**, *6*, 102068.
[32] Z. He, G. Cheng, Y. Jiang, L. Wang, L. Dai, *J. Electrochem. Soc.* **2018**, *5*, A932.
[33] S. Wang, X. Zhao, T. Cochell, A. Manthiram, *J. Phys. Chem. Lett* **2012**, *3*, 34.
[34] C. Noh, B. W. Kwon, Y. Chung, Y. Kwon, *J. Power Sources* **2018**, *406*, 26.
[35] C. Li, B. Xie, J. Chen, J. He, Z. He, *RSC Adv.* **2017**, *7*, 13184.
[36] M. Park, Y. Jung, J. Kim, H. il Lee, J. Cho, *Nano Lett.* **2013**, *13*, 4833.
[37] M. Shin, C. Noh, Y. Chung, D.-H. Kim, Y. Kwon, *Appl. Surf. Sci.* **2021**, *550*, 148977.
[38] A. Sankar, I. Michos, I. Dutta, J. Dong, A. P. Angelopoulos, *J. Power Sources* **2018**, *387*, 91.
[39] M. Park, I. Y. Jeon, J. Ryu, H. Jang, J. B. Back, J. Cho, *Nano Energy* **2016**, *26*, 233.
[40] Y. Zhou, L. Liu, Y. Shen, L. Wu, L. Yu, F. Liang, J. Xi, *Chem. Commun.* **2017**, *53*, 7565.
[41] Z. He, Y. Jiang, Y. Wei, C. Zhao, F. Jiang, L. Li, H. Zhou, W. Meng, L. Wang, L. Dai, *Electrochim. Acta* **2018**, *259*, 122.
[42] L. Wu, Y. Shen, L. Yu, J. Xi, X. Qiu, *Nano Energy* **2016**, *28*, 19.
[43] M. Park, J. Ryu, Y. Kim, J. Cho, *Energy Environ. Sci.* **2014**, *7*, 3727.
[44] Z. González, C. Botas, C. Blanco, R. Santamaría, M. Granda, P. Álvarez, R. Menéndez, *J. Power Sources* **2013**, *241*, 349.
[45] G. Hu, M. Jing, D. W. Wang, Z. Sun, C. Xu, W. Ren, H. M. Cheng, C. Yan, X. Fan, F. Li, *Energy Storage Mater.* **2018**, *13*, 66.
[46] T.-M. Tseng, R.-H. Huang, C.-Y. Huang, K.-L. Hseuh, F.-S. Shieu, *J. Electrochem. Soc.* **2013**, *160*, A690.
[47] Y. Liu, F. Liang, Y. Zhao, L. Yu, L. Liu, J. Xi, *J. Energy Chem.* **2018**, *27*, 1333.
[48] T. Liu, X. Li, H. Nie, C. Xu, H. Zhang, *J. Power Sources* **2015**, *286*, 73.
[49] Y. Lv, J. Zhang, Z. Lv, C. Wu, Y. Liu, H. Wang, S. Lu, Y. Xiang, *Electrochim. Acta* **2017**, *253*, 78.
[50] X. Yang, T. Liu, C. Xu, H. Zhang, X. Li, H. Zhang, *J. Energy Chem.* **2017**, *26*, 1.
[51] B. Li, M. Gu, Z. Nie, Y. Shao, Q. Luo, X. Wei, X. Li, J. Xiao, C. Wang, V. Sprenkle, W. Wang, *Nano Lett.* **2013**, *13*, 1330.
[52] L. Wei, T. S. Zhao, L. Zeng, X. L. Zhou, Y. K. Zeng, *Appl. Energy* **2016**, *180*, 386.
[53] Q. Zhang, T. Liu, H. Zhang, X. Li, *ACS Appl. Energy Mater.* **2021**, *4*, 3913.
[54] S. Mehboob, A. Mehmood, J.-Y. Lee, H.-J. Shin, J. Hwang, S. Abbas, H. Y. Ha, *J. Mater. Chem. A* **2017**, *5*, 17388.
[55] J. Shen, S. Liu, Z. He, L. Shi, *Electrochim. Acta* **2015**, *151*, 297.
[56] W. H. Wang, X. D. Wang, *Electrochim. Acta* **2007**, *52*, 6755.
[57] H. Zhou, Y. Shen, J. Xi, X. Qiu, L. Chen, *ACS Appl. Mater. Interfaces* **2016**, *8*, 15369.
[58] S. Mehboob, G. Ali, H. J. Shin, J. Hwang, S. Abbas, K. Y. Chung, H. Y. Ha, *Appl. Energy* **2018**, *229*, 910.

[59] N. Yun, J. Jin, O. O. Park, K. Bong, J. Hoon, *Electrochim. Acta* **2018**, *278*, 226.
[60] D. You, J. Lou, X. Li, Y. Zhou, X. Sun, X. Wang, *J. Power Sources* **2021**, *494*, 229775.
[61] Y. Xiang, W. A. Daoud, *Electrochim. Acta* **2018**, *290*, 176.
[62] Y. Xiang, W. A. Daoud, *J. Mater. Chem. A* **2019**, *7*, 5589.
[63] A. W. Bayeh, D. M. Kabtamu, Y.-C. Chang, G.-C. Chen, H.-Y. Chen, G.-Y. Lin, T.-R. Liu, T. H. Wondimu, K.-C. Wang, C.-H. Wang, *ACS Sustain. Chem. Eng.* **2018**, *6*, 3019.
[64] A. Fetyan, G. A. El-Nagar, I. Derr, P. Kubella, H. Dau, C. Roth, *Electrochim. Acta* **2018**, *268*, 59.
[65] K. J. Kim, M.-S. Park, J.-H. Kim, U. Hwang, N. J. Lee, G. Jeong, Y.-J. Kim, *Chem. Commun* **2012**, *48*, 5455.
[66] J. Vázquez-Galván, C. Flox, C. Fàbrega, E. Ventosa, A. Parra, T. Andreu, J. R. Morante, *ChemSusChem* **2017**, *10*, 2089.
[67] H. R. Jiang, W. Shyy, Y. X. Ren, R. H. Zhang, T. S. Zhao, *Appl. Energy* **2019**, *233–234*, 544.
[68] H. Zhou, J. Xi, Z. Li, Z. Zhang, L. Yu, L. Liu, X. Qiu, L. Chen, *RSC Adv.* **2014**, *4*, 61912.
[69] L. Xia, T. Long, W. Li, F. Zhong, M. Ding, Y. Long, Z. Xu, Y. Lei, Y. Guan, D. Yuan, Y. Zhang, C. Jia, L. Sun, Q. Sun, *Small* **2020**, *16*, 2003321.
[70] M. Jing, X. Zhang, X. Fan, L. Zhao, J. Liu, C. Yan, *Electrochim. Acta* **2016**, *215*, 57.
[71] Z. He, M. Li, Y. Li, J. Zhu, Y. Jiang, W. Meng, H. Zhou, L. Wang, L. Dai, *Electrochim. Acta* **2018**, *281*, 601.
[72] B. Li, M. Gu, Z. Nie, X. Wei, C. Wang, V. Sprenkle, W. Wang, *Nano Lett.* **2014**, *14*, 158.
[73] J. Noack, L. Wietschel, N. Roznyatovskaya, K. Pinkwart, J. Tübke, *Energies* **2016**, *9*, 627.
[74] A. Larsson, Evaluation of Flow Battery Technology: An Assessment of Technical and Economic Feasibility, Massachusetts Institute of Technology, **2009**.
[75] C. Minke, T. Turek, *J. Power Sources* **2018**, *376*, 66.
[76] V. Viswanathan, A. Crawford, D. Stephenson, S. Kim, W. Wang, B. Li, G. Coffey, E. Thomsen, G. Graff, P. Balducci, M. Kintner-Meyer, V. Sprenkle, *J. Power Sources* **2014**, *247*, 1040.
[77] “40ft Hard Top Buying | CARU Containers,” can be found under https://www.carucontainers.com, **n.d.**
[78] *Ryan Herco Flow Solutions: A SunSource Company Product Guide (29th Edition)*, **2019**.
[79] A. Wheatley, D. Warren, S. Das, *Advanced Composite Materials for Automotive Applications*, John Wiley & Sons Ltd., Chichester, UK, **2014**.
[80] C. Minke, U. Kunz, T. Turek, *J. Power Sources* **2017**, *342*, 116.
[81] “Battery Felts for Redox Flow Batteries | SGL Carbon,” can be found under https://www.sglcarbon.com/en/markets-solutions/material/sigracell-battery-felts/, accessed: 12, **2022**.
[82] A. S. Gill, D. Visotsky, L. Mears, M. Asme, J. D. Summers, *J. Manuf. Sci. Eng.* **2017**, *139*, 041011.
[83] D. Warren, *Low Cost Carbon Fiber Overview*, **2013**.
[84] D. Warren, *Carbon Fiber Precursors and Conversion*, **2013**.
[85] D. Warren, *Future Lower Cost Carbon Fiber for Autos: International Scale-up & What Is Needed*, **2007**.
[86] F. Nakao, H. Uno, *Surface Treatment Process for Carbon Fibers*, **1988**.
[87] “Specific Heat Capacities of Air,” can be found under https://www.ohio.edu/mechanical/thermo/property_tables/air/air_Cp_Cv.html, accessed: 12, **2022.**
[88] “Purity Plus Catalog | Cryogenic Gases,” can be found under https://cryogenicgas.com/mdocuments-library/, accessed: 12, **2022.**
[89] M. W. J. Chase, *NIST-JANAF Themochemical Tables*, **1998**.
